# Genetic Variants in Epidermal Growth Factor Receptor Pathway Genes and Risk of Esophageal Squamous Cell Carcinoma and Gastric Cancer in a Chinese Population

**DOI:** 10.1371/journal.pone.0068999

**Published:** 2013-07-18

**Authors:** Wen-Qing Li, Nan Hu, Zhaoming Wang, Kai Yu, Hua Su, Lemin Wang, Chaoyu Wang, Stephen J. Chanock, Laurie Burdett, Ti Ding, You-Lin Qiao, Jin-Hu Fan, Yuan Wang, Yi Xu, Carol Giffen, Xiaoqin Xiong, Gwen Murphy, Margaret A. Tucker, Sanford M. Dawsey, Neal D. Freedman, Christian C. Abnet, Alisa M. Goldstein, Philip R. Taylor

**Affiliations:** 1 Division of Cancer Epidemiology and Genetics, National Cancer Institute, National Institutes of Health, Bethesda, Maryland, United States of America; 2 Cancer Genomics Research Laboratory, NCI-Frederick, SAIC-Frederick Inc., Frederick, Maryland, United States of America; 3 Shanxi Cancer Hospital, Taiyuan, Shanxi, People’s Republic of China; 4 Department of Epidemiology, Cancer Institute (Hospital), Chinese Academy of Medical Sciences, Beijing, People’s Republic of China; 5 Information Management Services, Inc., Silver Spring, Maryland, United States of America; Centro di Riferimento Oncologico, IRCCS National Cancer Institute, Italy

## Abstract

The epidermal growth factor receptor (EGFR) signaling pathway regulates cell proliferation, differentiation, and survival, and is frequently dysregulated in esophageal and gastric cancers. Few studies have comprehensively examined the association between germline genetic variants in the EGFR pathway and risk of esophageal and gastric cancers. Based on a genome-wide association study in a Han Chinese population, we examined 3443 SNPs in 127 genes in the EGFR pathway for 1942 esophageal squamous cell carcinomas (ESCCs), 1758 gastric cancers (GCs), and 2111 controls. SNP-level analyses were conducted using logistic regression models. We applied the resampling-based adaptive rank truncated product approach to determine the gene- and pathway-level associations. The EGFR pathway was significantly associated with GC risk (*P* = 2.16×10^−3^). Gene-level analyses found 10 genes to be associated with GC, including *FYN*, *MAPK8*, *MAP2K4*, *GNAI3*, *MAP2K1*, *TLN1*, *PRLR*, *PLCG2*, *RPS6KB2*, and *PIK3R3* (*P*<0.05). For ESCC, we did not observe a significant pathway-level association (*P* = 0.72), but gene-level analyses suggested associations between *GNAI3*, *CHRNE*, *PAK4*, *WASL*, and *ITCH*, and ESCC (*P*<0.05). Our data suggest an association between specific genes in the EGFR signaling pathway and risk of GC and ESCC. Further studies are warranted to validate these associations and to investigate underlying mechanisms.

## Introduction

ERBBs or epidermal growth factor receptors (EGFRs) belong to the receptor tyrosine kinase (RTK) superfamily and are important signaling proteins in normal physiological conditions [1], [2]. For example, ligand-bound EGFRs are regulators of cell-cycle progression, proliferation, survival, invasion, and other cancer contributing processes [3], [4]. Not surprisingly, therefore, members of the EGFR family, particularly EGFR (also known as ERBB1 or HER1) and ERBB2 (HER2), have been implicated in the development of numerous human cancers and are pursued as therapeutic targets [3], [4], [5]. In regards to esophageal and gastric cancer, higher EGFR and ERBB2 levels have been correlated with poor esophageal and gastric cancer survival [4], [6], [7]. Therapies targeting the EGFR family were shown to improve esophageal and gastric cancer prognosis [4]. Several studies have also revealed somatic mutations of genes in the EGFR family in esophageal and gastric cancers [8], [9], [10], [11], [12]. In addition, a role for downstream signaling of the EGFR family has also been found, with molecules involved in the MAPK/ERK pathway activated in esophageal and gastric cancers [13], [14], [15].

Given the significance of this pathway, genetic variations in EGFR signaling proteins could correlate with predisposition to esophageal and gastric cancers. However, only a few studies have investigated the role of germline single nucleotide polymorphisms (SNPs) in these cancers. These few prior studies had only limited coverage of the genes in this pathway [16], [17], [18], [19], [20], [21], [22], [23]. Although SNPs in this pathway have not reached genome-wide significance in published genome-wide association studies (GWAS) [24], [25], [26], [27], [28], [29], [30], [31], [32], such a criteria may be overly conservative for detecting modest associations. Therefore, pathway analysis may help to identify important genetic contributions whose individual effect sizes may be too small to be detected using the GWAS significance criteria [33], [34]. Based on our GWAS data in ethnic Chinese subjects [24], we comprehensively evaluated associations between genetic variants in the EGFR pathway and the risk of esophageal squamous cell carcinoma (ESCC) and gastric cancer (GC) in 1942 ESCC cases, 1758 GC cases (1126 cases of gastric cardia cancer (GCA) and 632 of gastric noncardia cancer (GNCA)), and 2111 controls living in the Taihang Mountain region of China, an area with a high risk of ESCC and GC.

## Materials and Methods

### Ethics Statement

The Shanxi upper gastrointestinal (UGI) Cancer Genetics Project (Shanxi, registered at ClinicalTrials.gov as NCT00341276) obtained written informed consent from subjects to attend the Shanxi parent study and the overall GWAS (current study) and the whole procedures were approved by Shanxi Cancer Hospital and Institute Institutional Review Board. The Linxian Nutrition Intervention Trials (NITs, registered at ClinicalTrials.gov as NCT00342654) obtained written informed consent from subjects to attend the NIT parent study and the overall GWAS (current study) and the whole procedures were approved by Cancer Institute of the Chinese Academy of Medical Sciences Institutional Review Board. The NCI Special Studies Institutional Review Board approved both the Shanxi and NIT parent studies as well as the overall GWAS (current study).

### Study Population

The study participants were enrolled from two upper gastrointestinal (UGI) cancer projects conducted in the Taihang Mountain area in China: the Shanxi and NITs study. The Shanxi study was initiated in 1997 and had a case-control portion and a case-only portion. We enrolled newly-diagnosed, histologically-confirmed ESCC and GC cases, and, in the case-control portion of this study, age (±5 years)-, sex-, and neighborhood-matched controls were enrolled within 6 months of the identification of each case [35]. Blood samples were collected at enrollment. The NITs were initiated in Linxian in 1985 and tested the effect of multiple vitamin and mineral combinations taken daily for up to six years on the outcome of esophageal and gastric cancers [36]. We collected blood in 1999 and 2000 specifically to obtain DNA from NIT participants. During the follow-up through December 31, 2010, all newly-diagnosed, histologically confirmed ESCC and GC cases along with controls from an age- and gender-stratified randomly sampled subcohort, were included in the current genetic analysis. All examined esophageal cancers were ESCC, and all GCs were adenocarcinomas. GCAs were defined as those located in the proximal 3 cm of the stomach, whereas GNCAs were those in the remainder of the stomach.

### Gene and SNP Selection

We performed an extensive literature search of the EGFR pathway genes [1], [2], [3], [4], [5]. A gene was included in our analysis if it was referenced in at least one of the following databases: ErbB signaling pathway in KEGG (http://www.genome.jp/dbget-bin/www_bget?pathway:map04012, retrieved Dec 20, 2012), EGF signaling pathway in BioCarta (http://www.biocarta.com/pathfiles/h_egfPathway.asp, retrieved Dec 20, 2012), or ErbB receptor signaling, ErbB2/ErbB3 signaling, EGF receptor signaling, or ErbB4 signaling pathway in the NCI Pathway Interaction Database (http://pid.nci.nih.gov/browse_pathways.shtml, retrieved Dec 20, 2012). We identified a total of 131 EGFR pathway genes. No SNPs mapped to *AREGB*, *EIF4EBP1*, *PAK3*, and *SHC1* in our dataset, leaving 127 genes (Table S1) for analysis. A total of 3443 SNPs located within these genes and their flanking areas (20 kb upstream and 10 kb downstream), with a minor allele frequency of >1% (in cases and controls combined) were included in our analysis, and the full list of these SNPs were shown in Table S2.

### Genotyping and quality control

Genome-wide scanning was performed using the Illumina 660W array, which has been detailed in our published GWAS on UGI cancer [24]. After that report, we scanned additional subjects on the same platform at the same facility. The initial and additional subject scan data underwent similar processing and quality control filtering metrics. We excluded SNPs with a missing rate >5%, subjects with a completion rate of all SNPs <94%, subjects with abnormal mean heterozygosity values (>30% or <25%), gender discordant subjects, or unexpected duplicate pairs. The GWAS data on UGI cancer in the study populations have been deposited on the database of Genotypes and Phenotypes (dbGaP, http://www.ncbi.nlm.nih.gov/projects/gap/, study accession number: phs000361.v1.p1).

### Statistical analysis

We investigated the association between genes in the EGFR signaling pathway and risk of ESCC and GC. To conduct gene-level analysis, we first carried out SNP-level analysis. We calculated the odds ratios (ORs) and 95% confidence intervals (CIs) for risk of ESCC or GC associated with having one minor allele, using unconditional logistic regression in an additive model for each SNP, adjusting for age, gender, and study (Shanxi or NIT). We did not consider population stratification because there was no evidence for significant problems with population substructure [24]. We used a dominant model for SNPs when the expected number of subjects carrying the minor allele was less than five.

Gene-level associations were then calculated using the adaptive rank truncated product (ARTP) approach, which applied rank-truncated test statistics and a permutation-based sampling procedure (1,000,000 resamplings) [34]. Association signals over a set of SNPs within a gene were combined while accounting for SNP linkage disequilibrium (LD) structures and multiple comparisons. We also evaluated the association of the overall EGFR pathway with ESCC and GC, which globally combined the associations between each outcome and genes within the pathway. We used the ARTP method with 1,000,000 resamplings to obtain a single summary pathway-level *P*-value for each cancer type.

In secondary analyses, we additionally adjusted for cigarette smoking (ever or never), alcohol intake (ever or seldom/never), and family history of UGI cancer (yes or no). Since results of these SNP-level secondary analyses showed essentially similar results as those from the primary models, we present only the primary analyses in the paper.

We tested the association between SNPs and ESCC and GC by subgroups of sex, smoking, alcohol intake, and family history of UGI cancer. The *P* for interactions between SNPs and these variables were examined using likehood ratio tests.

Statistical significance for gene- and pathway-based analyses was defined as *P*<0.05. Since none of the SNPs reached the Bonferroni-corrected significance level (1.45×10^−5^, 0.05/3443 SNPs), statistical significance for SNP-level analyses was defined as *P*<0.001. Statistical analyses were performed using R language. We evaluated the linkage disequilibrium (LD) between SNPs across specific gene regions with Haploview version 4.1.

## Results

A total of 1942 cases of ESCC, 1758 cases of GC (1126 GCA and 632 GNCA cases), and 2111 controls were included from the Shanxi and NIT studies (Table S3). Overall, the mean age was 56.0 years in controls, 56.0 in ESCCs, and 56.3 in GCs.

We conducted gene-level analysis among the 127 genes, and identified five genes, including *GNAI3*, *CHRNE*, *PAK4*, *WASL*, and *ITCH*, that were significantly associated with ESCC risk (*P*<0.05) (Table 1). Ten genes were significantly associated with risk of GC, including *FYN*, *MAPK8*, *MAP2K4*, *GNAI3*, *MAP2K1*, *TLN1*, *PRLR*, *PLCG2*, *RPS6KB2*, and *PIK3R3* (*P*<0.05) (Table 2). Among the GC-associated genes, *GNAI3*, *MAP2K1*, *FYN*, and *MAPK8* were associated with GCA, and *MAPK8*, *TLN1*, *RPS6KB2*, *MAP2K4*, and *PIK3R3* were associated with GNCA (*P*<0.05). We also identified several additional genes associated only with GCA (*TGFA*, *RASA1*, *JAK2*, *HSP90AA1*, *DLG4*, and *CHRNE*) or GNCA (*NEDD4*, *PTK2*, *HBEGF*, *CHRNA1*), but not with total GC. Genes with the strongest associations were *GNAI3* for ESCC (*P* = 8.17×10^−3^), *FYN* for total GC (*P* = 2.63×10^−3^), *GNAI3* for GCA (*P* = 4.50×10^−3^), and *MAPK8* for GNCA (*P* = 3.79×10^−3^), but none exceeded the Bonferroni-adjusted threshold (*P* = 3.94×10^−4^, 0.05/127 genes) (Table S1). Among examined genes, *GNAI3* and *CHNRE* were associated with both ESCC and GCA. The most significant SNP in *GNAI3* was the same for ESCC and GCA **(**rs1434285), but the most significant SNPs in *CHRNE* were different for ESCC (rs8081611) and GCA (rs3760490), and these two SNPs were not in high LD (r^2^ = 0.007).

**Table 1:** Epidermal growth factor receptor pathway genes significantly associated with risk of esophageal squamous cell carcinoma*.

Gene	Chr. (cytoband)	Gene-level *P*	No. of SNP	Most significant SNP analysis			
				dbSNP id (major,minor allele)	MAF: cases,controls	OR	*P*
*GNAI3*	1p13	8.17×10^−3^	7	rs1434285 (G, T)	0.303, 0.272	1.17	2.05×10^−3^
*CHRNE*	17p13	0.018	5	rs8081611 (T, C)	0.036, 0.050	0.73	3.99×10^−3^
*PAK4*	19q13	0.029	11	rs7257109 (G, A)	0.452, 0.422	1.14	3.44×10^−3^
*WASL*	7q31	0.032	7	rs2109725 (T, C)	0.441, 0.470	0.88	6.15×10^−3^
*ITCH*	20q11	0.048	2	rs6120650 (G, A)	0.337, 0.360	0.90	0.026

Abbreviations: MAF, minor allele frequency; OR, odds ratio; SNP, single nucleotide polymorphism.

* Genes with *P*-value <0.05 are listed and ordered by *P*-values. Gene-level *P-*values were calculated using the adaptive rank truncated product approach. The *P*-values and ORs for the SNPs were calculated from unconditional logistic regression models using genotype trend tests adjusted for age (10-year categories), sex and study.

**Table 2:** Epidermal growth factor receptor pathway genes significantly associated with risk of gastric adenocarcinoma overall and by anatomic sites*.

Gene	Chr. (cytoband)	Gene-level *P*	No. ofSNP	Most significant SNP analysis			
				dbSNP id (major,minor allele)	MAF: cases,controls	OR	*P*
**Total**							
* FYN*	6q21	2.63×10^−3^	38	rs9387033 (T, C)	0.240, 0.278	0.81	1.08×10^−4^
* MAPK8*	10q11	3.39×10^−3^	5	rs10508902 (A, G)	0.024, 0.014	1.74	1.53×10^−3^
* MAP2K4*	17p12	3.60×10^−3^	15	rs9788973 (C, T)	0.432, 0.391	1.18	3.46×10^−4^
* GNAI3*	1p13	6.86×10^−3^	7	rs1434285 (G, T)	0.305, 0.273	1.17	1.71×10^−3^
* MAP2K1*	15q22	0.017	6	rs12050732 (A, C)	0.393, 0.424	0.88	4.30×10^−3^
* TLN1*	9p13	0.019	6	rs2295795 (G, A)	0.270, 0.299	0.86	4.40×10^−3^
* PRLR*	5p13	0.019	45	rs7720677 (T, C)	0.430, 0.468	0.85	6.27×10^−4^
* PLCG2*	16q23	0.029	93	rs7187863 (A, G)	0.040, 0.056	0.68	6.13×10^−4^
* RPS6KB2*	11q13	0.042	4	rs1638588 (C, A)	0.348, 0.322	1.12	0.017
* PIK3R3*	1p34	0.046	9	rs9429095 (G, T)	0.242, 0.269	0.87	0.011
**Cardia cancer**							
* GNAI3*	1p13	4.50×10^−3^	7	rs1434285 (G, T)	0.311, 0.273	1.21	1.11×10^−3^
* TGFA* #	2p13	0.014	35	rs549386 (A, G)	0.276, 0.314	0.82	5.10×10^−4^
* RASA1* #	5q14	0.016	10	rs3752862 (A, G)	0.392, 0.353	1.18	3.24×10^−3^
* MAP2K1*	15q22	0.021	6	rs12050732 (A, C)	0.387, 0.424	0.86	5.23×10^−3^
* FYN*	6q21	0.029	38	rs9387033 (T, C)	0.240, 0.278	0.82	1.39×10^−3^
* JAK2* #	9p24	0.031	24	rs7850675 (A, C)	0.072, 0.097	0.74	2.51×10^−3^
* HSP90AA1* #	14q32	0.039	7	rs7160651 (G, A)	0.204, 0.177	1.19	9.22×10^−3^
* DLG4* #	17p13	0.040	9	rs314253 (G, A)	0.485, 0.481	1.15	6.94×10^−3^
* MAPK8*	10q11	0.042	5	rs10508902 (A, G)	0.023, 0.014	1.61	0.016
* CHRNE* #	17p13	0.045	5	rs3760490 (G, A)	0.087, 0.105	0.79	9.79×10^−3^
**Noncardia cancer**							
* MAPK8*	10q11	3.79×10^−3^	5	rs10508902 (A, G)	0.027, 0.014	1.97	2.49×10^−3^
* TLN1*	9p13	4.95×10^−3^	6	rs2295795 (G, A)	0.251, 0.299	0.78	1.12×10^−3^
* RPS6KB2*	11q13	0.011	4	rs1638588 (C, A)	0.366, 0.322	1.16	4.38×10^−3^
* MAP2K4*	17p12	0.012	15	rs9788973 (C, T)	0.441, 0.391	1.24	1.21×10^−3^
* NEDD4* #	15q21	0.018	31	rs8034917 (C, A)	0.339, 0.391	0.80	1.01×10^−3^
* PTK2* #	8q24	0.022	32	rs12545416 (T, C)	0.065, 0.043	1.55	2.72×10^−3^
* HBEGF* #	5q31	0.030	9	rs6879217 (C, T)	0.421, 0.379	1.20	6.29×10^−3^
* PIK3R3*	1p34	0.038	9	rs9429095 (G, T)	0.230, 0.269	0.82	8.83×10^−3^
* CHRNA1* #	2q31	0.041	8	rs12997022 (C, T)	0.090, 0.066	1.32	0.028

Abbreviations: MAF, minor allele frequency; OR, odds ratio; SNP, single nucleotide polymorphism.

* Genes with *P*-value <0.05 are listed and ordered by *P*-values. Gene-level P-values were calculated using adaptive rank truncated product approach. The *P*-values and ORs for the SNPs were calculated from unconditional logistic regression models using genotype trend tests adjusted for age (10-year categories), sex and study.

# These genes were significant only for cardia or noncardia cancer, but not for gastric cancer overall.

The pathway-level analysis revealed a statistically significant association of the overall EGFR pathway with GC risk (*P = *2.16×10^−3^), but not with ESCC risk (*P* = 0.72). However, the association was not significant for either GCA (*P = *0.12) or GNCA (*P* = 0.097).

The SNP-level associations are shown in Table 3 **.** Although none of the SNPs exceeded the significance level after correcting for multiple comparisons, at a reduced threshold of 0.001, rs1884361 (*NRG3*) was associated with ESCC risk, and rs9387033 (*FYN*), rs9788973 (*MAP2K4*), rs7187863 (*PLCG2*), and rs7720677 (*PRLR*) were associated with GC risk. We also identified a correlation for rs549386 (*TGFA*) with GCA, as well as correlation for rs16947307 and rs9923225 (both in *WWOX*) with GNCA. In the subgroup analyses, we did not observe significant interactions between SNPs and other characteristics at the threshold of 0.001 (data not shown).

**Table 3:** Top SNPs associated with risk of esophageal squamous cell carcinoma (ESCC) and gastric cancer (GC)^a^.

NCBI dbSNP identifier (major, minor allele)	Chr. (cytoband)	Gene	MAF: cases,controls	OR (95% CI)	*P*
**ESCC**					
rs1884361 (A, G)	10q23	*NRG3*	0.359, 0.321	1.17 (1.07–1.29)	6.84×10^−4^
rs9922434 (A, G)	16q23	*WWOX*	0.100, 0.080	1.30 (1.11–1.51)	1.09×10^−3^
rs9926713 (C, T)	16q23	*WWOX*	0.087, 0.107	0.78 (0.68–0.91)	1.68×10^−3^
**Total GC**					
rs9387033 (T, C)	6q21	*FYN*	0.240, 0.278	0.81 (0.73–0.90)	1.08×10^−4^
rs9788973 (C, T)	17p12	*MAP2K4*	0.432, 0.391	1.18 (1.08–1.29)	3.46×10^−4^
rs7187863 (A, G)	16q23	*PLCG2*	0.040, 0.056	0.68 (0.54–0.85)	6.13×10^−4^
rs7720677 (T, C)	5p13	*PRLR*	0.430, 0.468	0.85 (0.78–0.93)	6.27×10^−4^
rs1110898 (T, C)	16q23	*WWOX*	0.235, 0.203	1.20 (1.07–1.33)	1.31×10^−3^
rs10508902 (A, G)	10q11	*MAPK8*	0.024, 0.014	1.74 (1.24–2.45)	1.53×10^−3^
rs1434285 (G, T)	1p13	*GNAI3*	0.305, 0.273	1.17 (1.06–1.29)	1.71×10^−3^
**Cardia cancer**					
rs549386 (A, G)^b^	2p13	*TGFA*	0.276, 0.314	0.82 (0.73–0.91)	5.10×10^−4^
rs1434285 (G, T)	1p13	*GNAI3*	0.311, 0.273	1.21 (1.08–1.35)	1.11×10^−3^
rs9387033 (T, C)	6q21	*FYN*	0.240, 0.278	0.82 (0.73–0.93)	1.39×10^−3^
rs9788973 (C, T)	17p12	*MAP2K4*	0.426, 0.391	1.16 (1.04–1.28)	6.00×10^−3^
rs7187863 (A, G)	16q23	*PLCG2*	0.040, 0.056	0.69 (0.53–0.89)	4.37×10^−3^
rs7720677 (T, C)	5p13	*PRLR*	0.430, 0.468	0.85 (0.77–0.95)	3.05×10^−3^
rs1110898 (T, C)	16q23	*WWOX*	0.230, 0.203	1.17 (1.03–1.33)	0.013
rs10508902 (A, G)	10q11	*MAPK8*	0.023, 0.014	1.61 (1.09–2.37)	0.016
**Noncardia cancer**					
rs16947307 (C, T)^b^	16q23	*WWOX*	0.179, 0.224	0.75 (0.64–0.88)	5.13×10^−4^
rs9923225 (C, A)^b^	16q23	*WWOX*	0.267, 0.319	0.79 (0.68–0.90)	7.89×10^−4^
rs8034917 (C, A)^b^	15q21	*NEDD4*	0.334, 0.391	0.80 (0.70–0.91)	1.01×10^−3^
rs9788973 (C, T)	17p12	*MAP2K4*	0.441, 0.391	1.24 (1.09–1.41)	1.21×10^−3^
rs10508902 (A, G)	10q11	*MAPK8*	0.027, 0.014	1.97 (1.27–3.06)	2.49×10^−3^
rs1110898 (T, C)	16q23	*WWOX*	0.243, 0.203	1.25 (1.08–1.45)	3.54×10^−3^
rs9387033 (T, C)	6q21	*FYN*	0.239, 0.278	0.81 (0.70–0.94)	5.03×10^−3^
rs7187863 (A, G)	16q23	*PLCG2*	0.039, 0.056	0.64 (0.46–0.88)	6.76×10^−3^
rs7720677 (T, C)	5p13	*PRLR*	0.429, 0.468	0.85 (0.75–0.97)	0.015

Abbreviations: CI, confidence interval; ESCC, esophageal squamous cell carcinoma; GC, gastric cancer; MAF, minor allele frequency; OR, odds ratio; SNP, single nucleotide polymorphism.

a All SNPs with *P*-value <0.002 for esophageal squamous cell carcinoma (ESCC), gastric cancer (GC) overall or by anatomic sites are listed. The top SNPs for total GC (*P*-value <0.002) which have *P*-value <0.05 for cardia or noncardia cancer are also listed. Results were derived from logistic regression models using genotype trend tests adjusted for age (10-year categories), sex and study.

b These SNPs were significant only for gastric cardia or noncardia cancer, but not for total gastric cancer.

## Discussion

Somatic mutations and altered regulation of EGFR pathway genes have been widely implicated in the development and prognosis of esophageal and gastric cancers [6], [7], [8], [9], [10], [11], [12], [13], [14], [15]. In contrast, it is less clear whether germline genetic variants in the EGFR pathway are associated with these cancers. Recent GWASs have identified numerous risk loci associated with ESCC or GC, but thus far, there has been no evidence for an association with genetic variants in EGFR pathway. Pathway-based approaches have been developed to utilize genome-wide data more efficiently, and they hold the potential to yield novel findings [33], [34]. We comprehensively evaluated genes in the EGFR pathway and risk of ESCC and GC using the ARTP approach. Although none of the genes met the Bonferroni-correction for multiple comparisons, at a threshold of 0.05, we observed that several genes, as well as the overall EGFR pathway, were associated with risk of GC. The results also suggested associations between multiple EGFR-related genes and ESCC risk.

We identified five genes significantly associated with ESCC risk. Among them, *GNAI3* and *CHRNE* were significant in both ESCC and GCA, but not in GNCA. *GNAI3* in 1p13.3 encoding Guanine nucleotide-binding protein G(k) subunit alpha, was the most significant gene for ESCC and also correlated with risk of GC, particularly GCA. Guanine nucleotide-binding proteins (G proteins) are involved as modulators or transducers in various transmembrane signaling pathways. One previous study suggested an association between rs11184738 (*PRMT6*, located in 1p13.3) and ESCC risk in a GWAS scan but not in the validation stage [28]. *GNAI3* is located 2.3 Mbps downstream of *PRMT6*, and the top SNP in *GNAI3* (rs1434285) was not in high LD with rs11184738 in our GWAS dataset (r^2^<0.01). *CHRNE* in 17p13.2 encoding acetylcholine receptor subunit epsilon precursor, was correlated with risk of both ESCC and GCA, but not with GC overall. One GWAS reported that rs17761864 (*SMG6*, located in 17p13.3) was associated with risk of ESCC [32], but *SMG6* is located more than 2.5 Mbps downstream from *CHRNE*.

Ten genes were significantly associated with GC risk in our study. *FYN* in 6q21 was the most significant gene in GC overall, but was associated only with GCA and not with GNCA. FYN protein belongs to the membrane-associated Src tyrosine kinase family and has a pivotal role in cell adhesion, proliferation and apoptosis [37]. *MAPK8* in 10q11 was the most significant gene for GNCA and was also associated with GCA. MAPK8 is a member of the mitogen-activated protein kinases and is involved in cell proliferation, differentiation, apoptosis and transcription. Recent pathway-based research indicated that *MAPK8* was associated with rectal cancer and pancreatic cancer [38], [39].

Since the standard single-locus methods may miss SNPs with moderate effect size, we used a resampling-based ARTP method, which combines association signals across individual SNPs within a gene, to calculate gene-level associations. In addition to the above-highlighted genes, our results suggested that some other genes were also associated with risk of GC or ESCC, even though individual SNPs in these genes were not reported in previous GWAS studies. We also found significant genes in the gene-level analysis for which the individual SNPs were not significant in the pre-defined threshold for SNP-level analysis, underscoring the necessity of a more integrated understanding of the genetic contributions than the SNP-level perspective only.

Our results are biologically plausible. The EGFR family has been found to be upregulated and is the target of somatic mutations in UGI cancers, and a clinical trial indicated improved cancer prognosis for therapies targeting the EGFR family [4], [6], [7]. Prior studies have also reported the role of downstream signaling of EGFR family genes in UGI cancers. One recent report indicated that the MAPK pathway was commonly stimulated in esophagogastric cancer following activation of RTKs [13]. A second study showed that oncogenic CagA promoted GC risk by activating ERK signaling pathways [15].

Previous GWASs indicated genetic variants in *PLCE1* as common susceptibility loci for ESCC and GCA but not for GNCA [24], [27]. In our analyses, we found two genes significant for ESCC and GCA but not for GNCA, further suggesting that a common genetic mechanism might contribute to the development of ESCC and GCA.

In our study, we used prior biological knowledge to systematically investigate associations between genes in the EGFR pathway and risk of ESCC and GC in a high-risk population in north central China. To our knowledge, this is the first study to comprehensively investigate the role of genetic variation in EGFR pathway genes and risk of UGI cancers. The relatively large sample size allowed us to assess the associations for ESCC, GC overall and by anatomic sites with a reasonable power. We also acknowledge, however, the limitations of our study. First, we had no information on *Helicobacter pylori* (*H. pylori*) infection [40], which could be a concern particularly for the analysis of GNCA. However, a recent survey among NIT plasma samples showed a prevalence of *H. pylori* seropositivity of 96.6% among GNCA, 95.8% among GCA, and 93.9% among controls (unpublished data), using a multiplex assay with *H. pylori* positivity defined as three or more antigens being positive [41]. Although the multiplex method tends to be more sensitive than traditional ELISAs, this serological examination revealed a very high *H. pylori* infection rate in this area even among controls, suggesting that our results were less likely to be greatly distorted by the lack of information on *H. pylori* infection. Second, further replications in independent populations are required to determine if the associations we observed between EGFR pathway genes and the risk of ESCC and GC are real. Third, the pre-defined EGFR pathway that we tested may not represent all functionally-related EGFR genes due to limitations in current knowledge. Fourth, further generalizability to other populations requires caution since our study was conducted only among high-risk Han Chinese.

In conclusion, our study identified significant associations between the germline genetic variations of the overall EGFR signaling pathway and several individual genes and the risk of GC, as well as individual genes and the risk of ESCC, suggesting a possible role for EGFR pathway genes in the development of UGI cancers. Further studies are warranted to confirm the associations in independent populations and to explore the underlying biological mechanisms.

## Supporting Information

Table S1 **The associations between all EGFR pathway genes and risk of esophageal squamous cell carcinoma and gastric adenocarcinoma.**
(DOCX)

Table S2 **The NCBI dbSNP identifiers, located genes, and chromosomes of SNPs included in the study.**
(DOCX)

Table S3 **Characteristics of the study participants.**
(DOCX)

## References

[1] Hynes NE, Lane HA (2005) ERBB receptors and cancer: the complexity of targeted inhibitors. Nat Rev Cancer 5: 341–354. 15864276 10.1038/nrc1609

[2] Citri A, Yarden Y (2006) EGF-ERBB signalling: towards the systems level. Nat Rev Mol Cell Biol 7: 505–516. 16829981 10.1038/nrm1962

[3] Krause DS, Van Etten RA (2005) Tyrosine kinases as targets for cancer therapy. N Engl J Med 353: 172–187. 16014887 10.1056/NEJMra044389

[4] Okines A, Cunningham D, Chau I (2011) Targeting the human EGFR family in esophagogastric cancer. Nat Rev Clin Oncol 8: 492–503. 21468131 10.1038/nrclinonc.2011.45

[5] Baselga J, Swain SM (2009) Novel anticancer targets: revisiting ERBB2 and discovering ERBB3. Nat Rev Cancer 9: 463–475. 19536107 10.1038/nrc2656

[6] Dulak AM, Schumacher SE, van Lieshout J, Imamura Y, Fox C, et al (2012) Gastrointestinal adenocarcinomas of the esophagus, stomach, and colon exhibit distinct patterns of genome instability and oncogenesis. Cancer Res 72: 4383–4393. 22751462 10.1158/0008-5472.CAN-11-3893 PMC3432726

[7] Deng N, Goh LK, Wang H, Das K, Tao J, et al (2012) A comprehensive survey of genomic alterations in gastric cancer reveals systematic patterns of molecular exclusivity and co-occurrence among distinct therapeutic targets. Gut 61: 673–684. 22315472 10.1136/gutjnl-2011-301839 PMC3322587

[8] Corso G, Velho S, Paredes J, Pedrazzani C, Martins D, et al (2011) Oncogenic mutations in gastric cancer with microsatellite instability. Eur J Cancer 47: 443–451. 20937558 10.1016/j.ejca.2010.09.008

[9] Kwak EL, Jankowski J, Thayer SP, Lauwers GY, Brannigan BW, et al (2006) Epidermal growth factor receptor kinase domain mutations in esophageal and pancreatic adenocarcinomas. Clin Cancer Res 12: 4283–4287. 16857803 10.1158/1078-0432.CCR-06-0189 PMC3807136

[10] Lee JW, Soung YH, Seo SH, Kim SY, Park CH, et al (2006) Somatic mutations of ERBB2 kinase domain in gastric, colorectal, and breast carcinomas. Clin Cancer Res 12: 57–61. 16397024 10.1158/1078-0432.CCR-05-0976

[11] Soung YH, Lee JW, Kim SY, Wang YP, Jo KH, et al (2006) Somatic mutations of the ERBB4 kinase domain in human cancers. Int J Cancer 118: 1426–1429. 16187281 10.1002/ijc.21507

[12] Zang ZJ, Cutcutache I, Poon SL, Zhang SL, McPherson JR, et al (2012) Exome sequencing of gastric adenocarcinoma identifies recurrent somatic mutations in cell adhesion and chromatin remodeling genes. Nat Genet 44: 570–574. 22484628 10.1038/ng.2246

[13] Paterson AL, Shannon NB, Lao-Sirieix P, Ong CA, Peters CJ, et al.. (2012) A systematic approach to therapeutic target selection in oesophago-gastric cancer. Gut. [Epub ahead of print]. 10.1136/gutjnl-2012-302039 22773546

[14] Ma S, Bao JY, Kwan PS, Chan YP, Tong CM, et al.. (2012) Identification of PTK6, via RNA sequencing analysis, as a suppressor of esophageal squamous cell carcinoma. Gastroenterology 143: 675–686 e671–612. 10.1053/j.gastro.2012.06.007 22705009

[15] Yang JJ, Cho LY, Ma SH, Ko KP, Shin A, et al (2011) Oncogenic CagA promotes gastric cancer risk via activating ERK signaling pathways: a nested case-control study. PLoS One 6: e21155. 21698158 10.1371/journal.pone.0021155 PMC3116873

[16] Menke V, Pot RG, Moons LM, van Zoest KP, Hansen B, et al (2012) Functional single-nucleotide polymorphism of epidermal growth factor is associated with the development of Barrett’s esophagus and esophageal adenocarcinoma. J Hum Genet 57: 26–32. 22129558 10.1038/jhg.2011.124

[17] Ohmiya N, Taguchi A, Mabuchi N, Itoh A, Hirooka Y, et al (2006) MDM2 promoter polymorphism is associated with both an increased susceptibility to gastric carcinoma and poor prognosis. J Clin Oncol 24: 4434–4440. 16983111 10.1200/JCO.2005.04.1459

[18] Hong Y, Miao X, Zhang X, Ding F, Luo A, et al (2005) The role of P53 and MDM2 polymorphisms in the risk of esophageal squamous cell carcinoma. Cancer Res 65: 9582–9587. 16230424 10.1158/0008-5472.CAN-05-1460

[19] Cescon DW, Bradbury PA, Asomaning K, Hopkins J, Zhai R, et al (2009) p53 Arg72Pro and MDM2 T309G polymorphisms, histology, and esophageal cancer prognosis. Clin Cancer Res 15: 3103–3109. 19383811 10.1158/1078-0432.CCR-08-3120 PMC3081782

[20] Ma J, Zhang J, Ning T, Chen Z, Xu C (2012) Association of genetic polymorphisms in MDM2, PTEN and P53 with risk of esophageal squamous cell carcinoma. J Hum Genet 57: 261–264. 22336889 10.1038/jhg.2012.15

[21] Hildebrandt MA, Yang H, Hung MC, Izzo JG, Huang M, et al (2009) Genetic variations in the PI3K/PTEN/AKT/mTOR pathway are associated with clinical outcomes in esophageal cancer patients treated with chemoradiotherapy. J Clin Oncol 27: 857–871. 19164214 10.1200/JCO.2008.17.6297 PMC2738430

[22] Lanuti M, Liu G, Goodwin JM, Zhai R, Fuchs BC, et al (2008) A functional epidermal growth factor (EGF) polymorphism, EGF serum levels, and esophageal adenocarcinoma risk and outcome. Clin Cancer Res 14: 3216–3222. 18483390 10.1158/1078-0432.CCR-07-4932 PMC2572712

[23] Dong LM, Potter JD, White E, Ulrich CM, Cardon LR, et al (2008) Genetic susceptibility to cancer: the role of polymorphisms in candidate genes. JAMA 299: 2423–2436. 18505952 10.1001/jama.299.20.2423 PMC2772197

[24] Abnet CC, Freedman ND, Hu N, Wang Z, Yu K, et al (2010) A shared susceptibility locus in PLCE1 at 10q23 for gastric adenocarcinoma and esophageal squamous cell carcinoma. Nat Genet 42: 764–767. 20729852 10.1038/ng.649 PMC2947317

[25] Sakamoto H, Yoshimura K, Saeki N, Katai H, Shimoda T, et al (2008) Genetic variation in PSCA is associated with susceptibility to diffuse-type gastric cancer. Nat Genet 40: 730–740. 18488030 10.1038/ng.152

[26] Shi Y, Hu Z, Wu C, Dai J, Li H, et al (2011) A genome-wide association study identifies new susceptibility loci for non-cardia gastric cancer at 3q13.31 and 5p13.1. Nat Genet 43: 1215–1218. 22037551 10.1038/ng.978

[27] Wang LD, Zhou FY, Li XM, Sun LD, Song X, et al (2010) Genome-wide association study of esophageal squamous cell carcinoma in Chinese subjects identifies susceptibility loci at PLCE1 and C20orf54. Nat Genet 42: 759–763. 20729853 10.1038/ng.648

[28] Wu C, Hu Z, He Z, Jia W, Wang F, et al (2011) Genome-wide association study identifies three new susceptibility loci for esophageal squamous-cell carcinoma in Chinese populations. Nat Genet 43: 679–684. 21642993 10.1038/ng.849

[29] Abnet CC, Wang Z, Song X, Hu N, Zhou FY, et al (2012) Genotypic variants at 2q33 and risk of esophageal squamous cell carcinoma in China: a meta-analysis of genome-wide association studies. Hum Mol Genet 21: 2132–2141. 22323360 10.1093/hmg/dds029 PMC3315211

[30] Tanaka F, Yamamoto K, Suzuki S, Inoue H, Tsurumaru M, et al (2010) Strong interaction between the effects of alcohol consumption and smoking on oesophageal squamous cell carcinoma among individuals with ADH1B and/or ALDH2 risk alleles. Gut 59: 1457–1464. 20833657 10.1136/gut.2009.205724

[31] Cui R, Kamatani Y, Takahashi A, Usami M, Hosono N, et al (2009) Functional variants in ADH1B and ALDH2 coupled with alcohol and smoking synergistically enhance esophageal cancer risk. Gastroenterology 137: 1768–1775. 19698717 10.1053/j.gastro.2009.07.070

[32] Wu C, Kraft P, Zhai K, Chang J, Wang Z, et al (2012) Genome-wide association analyses of esophageal squamous cell carcinoma in Chinese identify multiple susceptibility loci and gene-environment interactions. Nat Genet 44: 1090–1097. 22960999 10.1038/ng.2411

[33] Wang K, Li M, Hakonarson H (2010) Analysing biological pathways in genome-wide association studies. Nat Rev Genet 11: 843–854. 21085203 10.1038/nrg2884

[34] Yu K, Li Q, Bergen AW, Pfeiffer RM, Rosenberg PS, et al (2009) Pathway analysis by adaptive combination of P-values. Genet Epidemiol 33: 700–709. 19333968 10.1002/gepi.20422 PMC2790032

[35] Gao Y, Hu N, Han XY, Ding T, Giffen C, et al (2011) Risk factors for esophageal and gastric cancers in Shanxi Province, China: a case-control study. Cancer Epidemiol 35: e91–99. 21846596 10.1016/j.canep.2011.06.006 PMC3215853

[36] Qiao YL, Dawsey SM, Kamangar F, Fan JH, Abnet CC, et al (2009) Total and Cancer Mortality After Supplementation With Vitamins and Minerals: Follow-up of the Linxian General Population Nutrition Intervention Trial. J Natl Cancer Inst 101: 507–518. 19318634 10.1093/jnci/djp037 PMC2664089

[37] Eguchi R, Kubo S, Takeda H, Ohta T, Tabata C, et al (2012) Deficiency of Fyn protein is prerequisite for apoptosis induced by Src family kinase inhibitors in human mesothelioma cells. Carcinogenesis 33: 969–975. 22354875 10.1093/carcin/bgs109

[38] Slattery ML, Lundgreen A, Wolff RK (2012) MAP kinase genes and colon and rectal cancer. Carcinogenesis 33: 2398–2408. 23027623 10.1093/carcin/bgs305 PMC3510742

[39] Li D, Duell EJ, Yu K, Risch HA, Olson SH, et al (2012) Pathway analysis of genome-wide association study data highlights pancreatic development genes as susceptibility factors for pancreatic cancer. Carcinogenesis 33: 1384–1390. 22523087 10.1093/carcin/bgs151 PMC3405651

[40] Ma JL, Zhang L, Brown LM, Li JY, Shen L, et al (2012) Fifteen-year effects of Helicobacter pylori,garlic, and vitamin treatments on gastric cancer incidence and mortality. J Natl Cancer Inst 104: 488–492. 22271764 10.1093/jnci/djs003 PMC3309129

[41] Michel A, Waterboer T, Kist M, Pawlita M (2009) Helicobacter pylori multiplex serology. Helicobacter 14: 525–535. 19889070 10.1111/j.1523-5378.2009.00723.x

