# Supplementary material for: Genetic Variants in Epidermal Growth Factor Receptor Pathway Genes and Risk of Esophageal Squamous Cell Carcinoma and Gastric Cancer in a Chinese Population
Source: PLoS One. 2013 Jul 18;8(7):e68999. doi: 10.1371/journal.pone.0068999 (PMC3715462; doi:10.1371/journal.pone.0068999)
Supplement: Table S1 — The associations between all EGFR pathway genes and risk of esophageal squamous cell carcinoma and gastric adenocarcinoma. (DOCX) [file pone.0068999.s001.docx]

**Table S1. The associations between all EGFR pathway genes and risk of esophageal squamous cell carcinoma (ESCC) and gastric adenocarcinoma**

| Gene | Location | No. of  SNPs | *P* value  for ESCC | *P* value for gastric adenocarcinoma | | |
| --- | --- | --- | --- | --- | --- | --- |
|  |  |  |  | Total | Cardia | Noncardia |
| *ABL1* | 9q34.12 | 26 | 0.213 | 0.320 | 0.467 | 0.171 |
| *ABL2* | [1q25.2](http://www.ncbi.nlm.nih.gov/entrez/query.fcgi?db=gene&cmd=Retrieve&dopt=full_report&list_uids=27) | 14 | 0.576 | 0.659 | 0.956 | 0.175 |
| *ADAM17* | 2p25.1 | 5 | 0.454 | 0.329 | 0.764 | 0.214 |
| *AKT1* | [14q32.33](http://www.ensembl.org/Homo_sapiens/geneview?gene=ENSG00000142208) | 4 | 0.843 | 0.504 | 0.285 | 0.094 |
| *AKT2* | [19q13.2](http://www.ensembl.org/Homo_sapiens/geneview?gene=ENSG00000105221) | 5 | 0.307 | 0.484 | 0.940 | 0.063 |
| *AKT3* | 1q44 | 26 | 0.557 | 0.094 | 0.276 | 0.215 |
| *ARAF* | [Xp11.23](http://www.ensembl.org/Homo_sapiens/geneview?gene=ENSG00000078061) | 4 | 0.590 | 0.526 | 0.188 | 0.825 |
| *AREG* | 4q13.3 | 2 | 0.434 | 0.990 | 0.870 | 0.891 |
| *BAD* | 11q13.1 | 4 | 0.434 | 0.225 | 0.888 | 0.067 |
| *BRAF* | 7q34 | 9 | 0.118 | 0.527 | 0.419 | 0.852 |
| *BTC* | 4q13.3 | 14 | 0.165 | 0.138 | 0.076 | 0.701 |
| *CAMK2A* | 5q32 | 31 | 0.135 | 0.912 | 0.900 | 0.895 |
| *CAMK2B* | 7p13 | 19 | 0.911 | 0.951 | 0.709 | 0.944 |
| *CAMK2D* | 4q26 | 54 | 0.195 | 0.748 | 0.616 | 0.599 |
| *CAMK2G* | 10q22.2 | 6 | 0.885 | 0.440 | 0.538 | 0.800 |
| *CBFA2T3* | 16q24.3 | 15 | 0.856 | 0.148 | 0.093 | 0.404 |
| *CBL* | 11q23.3 | 9 | 0.805 | 0.303 | 0.157 | 0.891 |
| *CBLB* | 3q13.11 | 40 | 0.613 | 0.813 | 0.491 | 0.079 |
| *CBLC* | [19q13.32](http://www.ensembl.org/Homo_sapiens/geneview?gene=ENSG00000142273) | 2 | 0.866 | 0.442 | 0.560 | 0.540 |
| *CDKN1A* | 6p21.2 | 10 | 0.501 | 0.182 | 0.477 | 0.228 |
| *CDKN1B* | [12p13.1](http://www.ensembl.org/Homo_sapiens/geneview?gene=ENSG00000111276) | 9 | 0.811 | 0.066 | 0.224 | 0.212 |
| *CHRNA1* | 2q31.1 | 8 | 0.063 | 0.380 | 0.232 | 0.041 |
| *CHRNE* | [17p13.2](http://www.ensembl.org/Homo_sapiens/geneview?gene=ENSG00000108556) | 5 | 0.018 | 0.099 | 0.045 | 0.404 |
| *CRK* | [17p13.3](http://www.ensembl.org/Homo_sapiens/geneview?gene=ENSG00000167193) | 6 | 0.456 | 0.735 | 0.764 | 0.217 |
| *CRKL* | 22q11.21 | 6 | 0.286 | 0.522 | 0.621 | 0.524 |
| *DLG4* | [17p13.1](http://www.ensembl.org/Homo_sapiens/geneview?gene=ENSG00000132535) | 9 | 0.974 | 0.147 | 0.040 | 0.470 |
| *DOCK7* | 1p31.3 | 10 | 0.062 | 0.060 | 0.069 | 0.116 |
| *EGF* | 4q25 | 21 | 0.206 | 0.291 | 0.435 | 0.203 |
| *EGFR* | 7p11.2 | 62 | 0.929 | 0.759 | 0.219 | 0.988 |
| *ELK1* | [Xp11.23](http://www.ensembl.org/Homo_sapiens/geneview?gene=ENSG00000126767) | 1 | 0.586 | 0.693 | 0.587 | 0.988 |
| *ERBB2* | [17q12](http://www.ensembl.org/Homo_sapiens/geneview?gene=ENSG00000141736) | 4 | 0.455 | 0.759 | 0.383 | 0.719 |
| *ERBB3* | [12q13.2](http://www.ensembl.org/Homo_sapiens/geneview?gene=ENSG00000065361) | 2 | 0.831 | 0.985 | 0.924 | 0.835 |
| *ERBB4* | 2q34 | 263 | 0.926 | 0.719 | 0.611 | 0.411 |
| *EREG* | 4q13.3 | 11 | 0.226 | 0.466 | 0.609 | 0.743 |
| *FOS* | [14q24.3](http://www.ensembl.org/Homo_sapiens/geneview?gene=ENSG00000170345) | 6 | 0.694 | 0.925 | 0.478 | 0.800 |
| *FRAP1* | 1p36.2 | 10 | 0.524 | 0.179 | 0.719 | 0.101 |
| *FYN* | 6q21 | 38 | 0.874 | 2.63×10^-3^ | 0.029 | 0.088 |
| *GAB1* | 4q31.21 | 15 | 0.365 | 0.322 | 0.330 | 0.861 |
| *GNAI1* | 7q21.11 | 23 | 0.324 | 0.963 | 0.667 | 0.689 |
| *GNAI3* | 1p13.3 | 7 | 8.17×10^-3^ | 6.86×10^-3^ | 4.50×10^-3^ | 0.329 |
| *GRB2* | [17q25.1](http://www.ensembl.org/Homo_sapiens/geneview?gene=ENSG00000177885) | 11 | 0.077 | 0.108 | 0.057 | 0.193 |
| *GRIN2B* | [12p13.1](http://www.ensembl.org/Homo_sapiens/geneview?gene=ENSG00000150086) | 146 | 0.880 | 0.961 | 0.796 | 0.838 |
| *GSK3B* | 3q13.33 | 14 | 0.902 | 0.142 | 0.146 | 0.667 |
| *GSN* | 9q33.2 | 10 | 0.685 | 0.806 | 0.760 | 0.770 |
| *HBEGF* | 5q31.3 | 9 | 0.664 | 0.168 | 0.826 | 0.030 |
| *HRAS* | 11p15.5 | 2 | 0.901 | 0.821 | 0.280 | 0.281 |
| *HSP90AA1* | [14q32.31](http://www.ensembl.org/Homo_sapiens/geneview?gene=ENSG00000080824) | 7 | 0.781 | 0.053 | 0.039 | 0.648 |
| *ITCH* | [20q11.22](http://www.ensembl.org/Homo_sapiens/geneview?gene=ENSG00000078747) | 2 | 0.048 | 0.603 | 0.541 | 0.479 |
| *JAK2* | 9p24.1 | 24 | 0.383 | 0.120 | 0.031 | 0.683 |
| *JUN* | 1p32.1 | 4 | 0.737 | 0.830 | 0.723 | 0.955 |
| *KRAS* | [12p12.1](http://www.ensembl.org/Homo_sapiens/geneview?gene=ENSG00000133703) | 16 | 0.580 | 0.405 | 0.376 | 0.774 |
| *LRIG1* | 3P14.1 | 36 | 0.225 | 0.629 | 0.078 | 0.384 |
| *MAP2K1* | [15q22.31](http://www.ensembl.org/Homo_sapiens/geneview?gene=ENSG00000169032) | 6 | 0.888 | 0.017 | 0.021 | 0.376 |
| *MAP2K2* | [19p13.3](http://www.ensembl.org/Homo_sapiens/geneview?gene=ENSG00000126934) | 12 | 0.327 | 0.654 | 0.797 | 0.726 |
| *MAP2K4* | [17p12](http://www.ensembl.org/Homo_sapiens/geneview?gene=ENSG00000065559) | 15 | 0.217 | 3.60×10^-3^ | 0.055 | 0.012 |
| *MAP2K7* | [19p13.2](http://www.ensembl.org/Homo_sapiens/geneview?gene=ENSG00000076984) | 4 | 0.948 | 0.949 | 0.875 | 0.818 |
| *MAP3K7IP2* | 6q25.1 | 12 | 0.180 | 0.462 | 0.395 | 0.809 |
| *MAPK1* | [22q11.22](http://www.ensembl.org/Homo_sapiens/geneview?gene=ENSG00000100030) | 9 | 0.369 | 0.508 | 0.367 | 0.910 |
| *MAPK10* | 4q21.3 | 61 | 0.077 | 0.122 | 0.114 | 0.143 |
| *MAPK3* | [16p11.2](http://www.ensembl.org/Homo_sapiens/geneview?gene=ENSG00000102882) | 3 | 0.986 | 0.870 | 0.965 | 0.871 |
| *MAPK8* | 10q11.22 | 5 | 0.320 | 3.39×10^-3^ | 0.042 | 3.79×10^-3^ |
| *MAPK9* | 5q35.3 | 17 | 0.382 | 0.722 | 0.728 | 0.883 |
| *MDM2* | [12q15](http://www.ensembl.org/Homo_sapiens/geneview?gene=ENSG00000135679) | 3 | 0.477 | 0.235 | 0.708 | 0.069 |
| *MYC* | 8q24.1 | 4 | 0.477 | 0.262 | 0.065 | 0.687 |
| *NCK1* | 3q22.3 | 4 | 0.560 | 0.608 | 0.580 | 0.872 |
| *NCK2* | 2q12.2 | 30 | 0.546 | 0.985 | 0.999 | 0.421 |
| *NCOR1* | [17p11.2](http://www.ensembl.org/Homo_sapiens/geneview?gene=ENSG00000141027) | 11 | 0.690 | 0.813 | 0.568 | 0.292 |
| *NEDD4* | [15q21.3](http://www.ensembl.org/Homo_sapiens/geneview?gene=ENSG00000069869) | 31 | 0.678 | 0.074 | 0.855 | 0.018 |
| *NF2* | [22q12.2](http://www.ensembl.org/Homo_sapiens/geneview?gene=ENSG00000186575) | 16 | 0.523 | 0.533 | 0.668 | 0.783 |
| *NFATC4* | [14q12](http://www.ensembl.org/Homo_sapiens/geneview?gene=ENSG00000100968) | 7 | 0.779 | 0.783 | 0.999 | 0.083 |
| *NRAS* | 1p13.2 | 4 | 0.310 | 0.962 | 0.862 | 0.994 |
| *NRG1* | 8p12 | 220 | 0.348 | 0.988 | 0.999 | 0.296 |
| *NRG2* | 5q31.2 | 32 | 0.449 | 0.725 | 0.565 | 0.843 |
| *NRG3* | 10q23.1 | 240 | 0.090 | 0.908 | 0.923 | 0.461 |
| *NRG4* | [15q24.2](http://www.ensembl.org/Homo_sapiens/geneview?gene=ENSG00000169752) | 6 | 0.464 | 0.401 | 0.642 | 0.382 |
| *PAK1* | 11q14.1 | 11 | 0.629 | 0.086 | 0.129 | 0.534 |
| *PAK2* | 3q29 | 10 | 0.518 | 0.051 | 0.125 | 0.172 |
| *PAK4* | [19q13.2](http://www.ensembl.org/Homo_sapiens/geneview?gene=ENSG00000130669) | 11 | 0.029 | 0.585 | 0.460 | 0.612 |
| *PAK6* | [15q15.1](http://www.ensembl.org/Homo_sapiens/geneview?gene=ENSG00000137843) | 15 | 0.794 | 0.497 | 0.811 | 0.403 |
| *PAK7* | [20p12.2](http://www.ensembl.org/Homo_sapiens/geneview?gene=ENSG00000101349) | 110 | 0.928 | 0.999 | 0.883 | 0.981 |
| *PIK3CA* | 3q26.32 | 9 | 0.625 | 0.559 | 0.209 | 0.557 |
| *PIK3CB* | 3q22.3 | 3 | 0.448 | 0.880 | 0.981 | 0.729 |
| *PIK3CD* | 1p36.2 | 9 | 0.054 | 0.721 | 0.611 | 0.893 |
| *PIK3CG* | 7q22.3 | 15 | 0.622 | 0.562 | 0.691 | 0.504 |
| *PIK3R1* | 5q13.1 | 27 | 0.517 | 0.516 | 0.251 | 0.307 |
| *PIK3R2* | [19p13.11](http://www.ensembl.org/Homo_sapiens/geneview?gene=ENSG00000105647) | 7 | 0.567 | 0.874 | 0.581 | 0.542 |
| *PIK3R3* | 1p34.1 | 9 | 0.678 | 0.046 | 0.373 | 0.038 |
| *PIK3R5* | [17p13.1](http://www.ensembl.org/Homo_sapiens/geneview?gene=ENSG00000141506) | 10 | 0.072 | 0.255 | 0.080 | 0.935 |
| *PIP5K1C* | [19p13.3](http://www.ensembl.org/Homo_sapiens/geneview?gene=ENSG00000186111) | 9 | 0.936 | 0.784 | 0.690 | 0.891 |
| *PLCG1* | [20q12](http://www.ensembl.org/Homo_sapiens/geneview?gene=ENSG00000124181) | 5 | 0.164 | 0.116 | 0.101 | 0.403 |
| *PLCG2* | [16q23.3](http://www.ensembl.org/Homo_sapiens/geneview?gene=ENSG00000197943) | 93 | 0.130 | 0.029 | 0.157 | 0.205 |
| *PPP3CB* | 10q22.2 | 3 | 0.894 | 0.672 | 0.726 | 0.500 |
| *PRKACA* | [19p13.12](http://www.ensembl.org/Homo_sapiens/geneview?gene=ENSG00000072062) | 3 | 0.668 | 0.130 | 0.224 | 0.261 |
| *PRKCA* | [17q24.2](http://www.ensembl.org/Homo_sapiens/geneview?gene=ENSG00000154229) | 117 | 0.872 | 0.473 | 0.703 | 0.717 |
| *PRKCB1* | [16p12.2](http://www.ensembl.org/Homo_sapiens/geneview?gene=ENSG00000166501) | 78 | 0.281 | 0.693 | 0.448 | 0.143 |
| *PRKCD* | 3p21.1 | 13 | 0.430 | 0.313 | 0.755 | 0.121 |
| *PRKCE* | 2p21 | 222 | 0.678 | 0.571 | 0.642 | 0.459 |
| *PRKCG* | [19q13.42](http://www.ensembl.org/Homo_sapiens/geneview?gene=ENSG00000126583) | 3 | 0.658 | 0.875 | 0.938 | 0.788 |
| *PRL* | 6p22.3 | 12 | 0.388 | 0.347 | 0.419 | 0.326 |
| *PRLR* | 5p13.2 | 45 | 0.073 | 0.019 | 0.084 | 0.317 |
| *PTEN* | 10q23.31 | 9 | 0.422 | 0.463 | 0.495 | 0.179 |
| *PTK2* | 8q24.3 | 32 | 0.113 | 0.054 | 0.261 | 0.022 |
| *PTPN1* | [20q13.13](http://www.ensembl.org/Homo_sapiens/geneview?gene=ENSG00000196396) | 17 | 0.720 | 0.330 | 0.504 | 0.437 |
| *PTPN11* | [12q24.13](http://www.ensembl.org/Homo_sapiens/geneview?gene=ENSG00000179295) | 4 | 0.843 | 0.966 | 0.666 | 0.322 |
| *PTPN6* | [12p13.31](http://www.ensembl.org/Homo_sapiens/geneview?gene=ENSG00000111679) | 3 | 0.368 | 0.186 | 0.378 | 0.303 |
| *RAF1* | 3p25.2 | 17 | 0.241 | 0.131 | 0.149 | 0.550 |
| *RASA1* | 5q14.3 | 10 | 0.056 | 0.060 | 0.016 | 0.071 |
| *RNF41* | [12q13.3](http://www.ensembl.org/Homo_sapiens/geneview?gene=ENSG00000181852) | 2 | 0.493 | 0.146 | 0.312 | 0.141 |
| *RPS6KB1* | [17q23.1](http://www.ensembl.org/Homo_sapiens/geneview?gene=ENSG00000108443) | 7 | 0.637 | 0.063 | 0.073 | 0.267 |
| *RPS6KB2* | 11q13.2 | 4 | 0.609 | 0.042 | 0.352 | 0.011 |
| *SHC2* | [19p13.3](http://www.ensembl.org/Homo_sapiens/geneview?gene=ENSG00000129946) | 11 | 0.149 | 0.073 | 0.189 | 0.252 |
| *SHC3* | 9q22.1 | 23 | 0.746 | 0.558 | 0.249 | 0.332 |
| *SHC4* | [15q21.1](http://www.ensembl.org/Homo_sapiens/geneview?gene=ENSG00000185634) | 48 | 0.250 | 0.590 | 0.378 | 0.425 |
| *SOS1* | 2p22.1 | 11 | 0.775 | 0.946 | 0.648 | 0.818 |
| *SOS2* | [14q21.3](http://www.ensembl.org/Homo_sapiens/geneview?gene=ENSG00000100485) | 10 | 0.533 | 0.947 | 0.410 | 0.888 |
| *SRC* | [20q11.23](http://www.ensembl.org/Homo_sapiens/geneview?gene=ENSG00000197122) | 9 | 0.797 | 0.143 | 0.279 | 0.403 |
| *STAT1* | 2p32.2 | 20 | 0.773 | 0.906 | 0.891 | 0.766 |
| *STAT3* | [17q21.2](http://www.ensembl.org/Homo_sapiens/geneview?gene=ENSG00000168610) | 8 | 0.649 | 0.462 | 0.519 | 0.204 |
| *STAT5A* | [17q21.2](http://www.ensembl.org/Homo_sapiens/geneview?gene=ENSG00000126561) | 3 | 0.762 | 0.137 | 0.239 | 0.243 |
| *STAT5B* | [17q11.2](http://www.ncbi.nlm.nih.gov/entrez/query.fcgi?db=gene&cmd=Retrieve&dopt=full_report&list_uids=6777) | 4 | 0.310 | 0.068 | 0.107 | 0.231 |
| *TGFA* | 2p13.3 | 35 | 0.522 | 0.050 | 0.014 | 0.443 |
| *TLN1* | 9p13.3 | 6 | 0.353 | 0.019 | 0.339 | 4.95×10^-3^ |
| *USP8* | [15q21.2](http://www.ensembl.org/Homo_sapiens/geneview?gene=ENSG00000138592) | 7 | 0.752 | 0.986 | 0.837 | 0.236 |
| *WASL* | 7q31.3 | 7 | 0.032 | 0.844 | 0.919 | 0.740 |
| *WWOX* | [16q23.1](http://www.ensembl.org/Homo_sapiens/geneview?gene=ENSG00000186153) | 416 | 0.271 | 0.317 | 0.749 | 0.142 |
| *WWP1* | 8q21.3 | 5 | 0.809 | 0.302 | 0.717 | 0.239 |
| *YAP1* | 11q22.1 | 18 | 0.387 | 0.854 | 0.972 | 0.864 |
