# Supplementary material for: Genetic Variants in Epidermal Growth Factor Receptor Pathway Genes and Risk of Esophageal Squamous Cell Carcinoma and Gastric Cancer in a Chinese Population
Source: PLoS One. 2013 Jul 18;8(7):e68999. doi: 10.1371/journal.pone.0068999 (PMC3715462; doi:10.1371/journal.pone.0068999)
Supplement: Table S2 — The NCBI dbSNP identifiers, located genes, and chromosomes of SNPs included in the study. (DOCX) [file pone.0068999.s002.docx]

**Table S2. The NCBI dbSNP identifiers, located genes, and chromosomes of SNPs included in the study^a^**

| Locus | Gene | CH. |  | Locus | Gene | CH. |  | Locus | Gene | CH. |
| --- | --- | --- | --- | --- | --- | --- | --- | --- | --- | --- |
| rs16853647 | *ABL2* | 1 |  | rs12514742 | *PRLR* | 5 |  | rs1509406 | *NEDD4* | 15 |
| rs1128952 | *ABL2* | 1 |  | rs4703508 | *PRLR* | 5 |  | rs6493827 | *NEDD4* | 15 |
| rs12143004 | *ABL2* | 1 |  | rs12521272 | *PRLR* | 5 |  | rs4774837 | *NEDD4* | 15 |
| rs3210839 | *ABL2* | 1 |  | rs12188786 | *PRLR* | 5 |  | rs9920283 | *NEDD4* | 15 |
| rs1325194 | *ABL2* | 1 |  | rs4703509 | *PRLR* | 5 |  | rs8029247 | *NEDD4* | 15 |
| rs3818433 | *ABL2* | 1 |  | rs7718468 | *PRLR* | 5 |  | rs11632974 | *NEDD4* | 15 |
| rs2274229 | *ABL2* | 1 |  | rs2047740 | *PRLR* | 5 |  | rs1509408 | *NEDD4* | 15 |
| rs9724754 | *ABL2* | 1 |  | rs11959588 | *PRLR* | 5 |  | rs2036741 | *NEDD4* | 15 |
| rs9728480 | *ABL2* | 1 |  | rs4235652 | *PRLR* | 5 |  | rs8025686 | *NEDD4* | 15 |
| rs2636278 | *ABL2* | 1 |  | rs1609500 | *PRLR* | 5 |  | rs16976674 | *NEDD4* | 15 |
| rs6672395 | *ABL2* | 1 |  | rs1604422 | *PRLR* | 5 |  | rs12592252 | *NRG4* | 15 |
| rs2791944 | *ABL2* | 1 |  | rs7731153 | *PRLR* | 5 |  | rs10444808 | *NRG4* | 15 |
| rs2171958 | *ABL2* | 1 |  | rs10064495 | *PRLR* | 5 |  | rs1499063 | *NRG4* | 15 |
| rs2050923 | *ABL2* | 1 |  | rs7729876 | *PRLR* | 5 |  | rs4886753 | *NRG4* | 15 |
| rs2994329 | *AKT3* | 1 |  | rs7735260 | *PRLR* | 5 |  | rs10851884 | *NRG4* | 15 |
| rs884808 | *AKT3* | 1 |  | rs9292578 | *PRLR* | 5 |  | rs4886755 | *NRG4* | 15 |
| rs9428576 | *AKT3* | 1 |  | rs3822482 | *PRLR* | 5 |  | rs11632939 | *PAK6* | 15 |
| rs1058304 | *AKT3* | 1 |  | rs13361664 | *PRLR* | 5 |  | rs1017843 | *PAK6* | 15 |
| rs1538773 | *AKT3* | 1 |  | rs7705676 | *PRLR* | 5 |  | rs2306480 | *PAK6* | 15 |
| rs7523198 | *AKT3* | 1 |  | rs7727306 | *PRLR* | 5 |  | rs11638855 | *PAK6* | 15 |
| rs6656918 | *AKT3* | 1 |  | rs7734446 | *PRLR* | 5 |  | rs2277562 | *PAK6* | 15 |
| rs1121276 | *AKT3* | 1 |  | rs12189146 | *PRLR* | 5 |  | rs11638662 | *PAK6* | 15 |
| rs3766673 | *AKT3* | 1 |  | rs7720677 | *PRLR* | 5 |  | rs2068001 | *PAK6* | 15 |
| rs2125231 | *AKT3* | 1 |  | rs7720773 | *PRLR* | 5 |  | rs731497 | *PAK6* | 15 |
| rs2290753 | *AKT3* | 1 |  | rs1567348 | *RASA1* | 5 |  | rs1075507 | *PAK6* | 15 |
| rs12691548 | *AKT3* | 1 |  | rs6893772 | *RASA1* | 5 |  | rs936216 | *PAK6* | 15 |
| rs7517732 | *AKT3* | 1 |  | rs12659717 | *RASA1* | 5 |  | rs2412504 | *PAK6* | 15 |
| rs2034915 | *AKT3* | 1 |  | rs6861271 | *RASA1* | 5 |  | rs6492939 | *PAK6* | 15 |
| rs2125230 | *AKT3* | 1 |  | rs13362486 | *RASA1* | 5 |  | rs12905386 | *PAK6* | 15 |
| rs10803155 | *AKT3* | 1 |  | rs10058449 | *RASA1* | 5 |  | rs2242119 | *PAK6* | 15 |
| rs12031994 | *AKT3* | 1 |  | rs3752862 | *RASA1* | 5 |  | rs4924445 | *PAK6* | 15 |
| rs10927067 | *AKT3* | 1 |  | rs10057748 | *RASA1* | 5 |  | rs1062124 | *SHC4* | 15 |
| rs2345994 | *AKT3* | 1 |  | rs10067098 | *RASA1* | 5 |  | rs1426199 | *SHC4* | 15 |
| rs897960 | *AKT3* | 1 |  | rs3827607 | *RASA1* | 5 |  | rs16961695 | *SHC4* | 15 |
| rs4132509 | *AKT3* | 1 |  | rs9368950 | *CDKN1A* | 6 |  | rs4775777 | *SHC4* | 15 |
| rs10157763 | *AKT3* | 1 |  | rs9462209 | *CDKN1A* | 6 |  | rs12906456 | *SHC4* | 15 |
| rs4614244 | *AKT3* | 1 |  | rs10807170 | *CDKN1A* | 6 |  | rs16961733 | *SHC4* | 15 |
| rs4430311 | *AKT3* | 1 |  | rs4713998 | *CDKN1A* | 6 |  | rs7183535 | *SHC4* | 15 |
| rs12753750 | *AKT3* | 1 |  | rs4713999 | *CDKN1A* | 6 |  | rs10519193 | *SHC4* | 15 |
| rs2953329 | *AKT3* | 1 |  | rs4714000 | *CDKN1A* | 6 |  | rs9806753 | *SHC4* | 15 |
| rs1167998 | *DOCK7* | 1 |  | rs3829963 | *CDKN1A* | 6 |  | rs10519197 | *SHC4* | 15 |
| rs10493325 | *DOCK7* | 1 |  | rs876581 | *CDKN1A* | 6 |  | rs4774527 | *SHC4* | 15 |
| rs10493326 | *DOCK7* | 1 |  | rs12207548 | *CDKN1A* | 6 |  | rs7162426 | *SHC4* | 15 |
| rs10889333 | *DOCK7* | 1 |  | rs6457940 | *CDKN1A* | 6 |  | rs16961813 | *SHC4* | 15 |
| rs10889337 | *DOCK7* | 1 |  | rs1726131 | *FYN* | 6 |  | rs16961815 | *SHC4* | 15 |
| rs13375691 | *DOCK7* | 1 |  | rs6916861 | *FYN* | 6 |  | rs12907348 | *SHC4* | 15 |
| rs12048208 | *DOCK7* | 1 |  | rs6919400 | *FYN* | 6 |  | rs4775785 | *SHC4* | 15 |
| rs2031373 | *DOCK7* | 1 |  | rs2282855 | *FYN* | 6 |  | rs8023445 | *SHC4* | 15 |
| rs10889353 | *DOCK7* | 1 |  | rs9374274 | *FYN* | 6 |  | rs934740 | *SHC4* | 15 |
| rs880694 | *DOCK7* | 1 |  | rs7746279 | *FYN* | 6 |  | rs934741 | *SHC4* | 15 |
| rs6540964 | *FRAP1* | 1 |  | rs706862 | *FYN* | 6 |  | rs7177968 | *SHC4* | 15 |
| rs2536 | *FRAP1* | 1 |  | rs1998038 | *FYN* | 6 |  | rs12593599 | *SHC4* | 15 |
| rs2275525 | *FRAP1* | 1 |  | rs11153311 | *FYN* | 6 |  | rs4381535 | *SHC4* | 15 |
| rs1417131 | *FRAP1* | 1 |  | rs809193 | *FYN* | 6 |  | rs7169672 | *SHC4* | 15 |
| rs1057079 | *FRAP1* | 1 |  | rs3752545 | *FYN* | 6 |  | rs1007662 | *SHC4* | 15 |
| rs1770345 | *FRAP1* | 1 |  | rs1022648 | *FYN* | 6 |  | rs2009404 | *SHC4* | 15 |
| rs2076655 | *FRAP1* | 1 |  | rs809192 | *FYN* | 6 |  | rs4774529 | *SHC4* | 15 |
| rs11121704 | *FRAP1* | 1 |  | rs706885 | *FYN* | 6 |  | rs10519199 | *SHC4* | 15 |
| rs12142905 | *FRAP1* | 1 |  | rs706895 | *FYN* | 6 |  | rs11070664 | *SHC4* | 15 |
| rs1074078 | *FRAP1* | 1 |  | rs697642 | *FYN* | 6 |  | rs12439459 | *SHC4* | 15 |
| rs501163 | *GNAI3* | 1 |  | rs6925303 | *FYN* | 6 |  | rs10519201 | *SHC4* | 15 |
| rs1434285 | *GNAI3* | 1 |  | rs6910116 | *FYN* | 6 |  | rs2413922 | *SHC4* | 15 |
| rs12044778 | *GNAI3* | 1 |  | rs9387033 | *FYN* | 6 |  | rs12440434 | *SHC4* | 15 |
| rs518076 | *GNAI3* | 1 |  | rs1465061 | *FYN* | 6 |  | rs4259993 | *SHC4* | 15 |
| rs7355073 | *GNAI3* | 1 |  | rs6914091 | *FYN* | 6 |  | rs4491452 | *SHC4* | 15 |
| rs2301230 | *GNAI3* | 1 |  | rs11964650 | *FYN* | 6 |  | rs10851473 | *SHC4* | 15 |
| rs505303 | *GNAI3* | 1 |  | rs11963612 | *FYN* | 6 |  | rs12323963 | *SHC4* | 15 |
| rs2760494 | *JUN* | 1 |  | rs11153318 | *FYN* | 6 |  | rs11070667 | *SHC4* | 15 |
| rs11688 | *JUN* | 1 |  | rs9384805 | *FYN* | 6 |  | rs12898878 | *SHC4* | 15 |
| rs11207306 | *JUN* | 1 |  | rs2148710 | *FYN* | 6 |  | rs11070668 | *SHC4* | 15 |
| rs2999138 | *JUN* | 1 |  | rs1327200 | *FYN* | 6 |  | rs12915940 | *SHC4* | 15 |
| rs926938 | *NRAS* | 1 |  | rs7761230 | *FYN* | 6 |  | rs4775789 | *SHC4* | 15 |
| rs8453 | *NRAS* | 1 |  | rs17072912 | *FYN* | 6 |  | rs11070670 | *SHC4* | 15 |
| rs10489525 | *NRAS* | 1 |  | rs2182644 | *FYN* | 6 |  | rs11636875 | *SHC4* | 15 |
| rs2144428 | *NRAS* | 1 |  | rs9372313 | *FYN* | 6 |  | rs16961946 | *SHC4* | 15 |
| rs9430506 | *PIK3CD* | 1 |  | rs1327202 | *FYN* | 6 |  | rs8031813 | *SHC4* | 15 |
| rs4129341 | *PIK3CD* | 1 |  | rs7749147 | *FYN* | 6 |  | rs12906122 | *SHC4* | 15 |
| rs6540985 | *PIK3CD* | 1 |  | rs12661059 | *FYN* | 6 |  | rs11631087 | *SHC4* | 15 |
| rs4240896 | *PIK3CD* | 1 |  | rs9320379 | *FYN* | 6 |  | rs7173509 | *SHC4* | 15 |
| rs12568084 | *PIK3CD* | 1 |  | rs2024832 | *FYN* | 6 |  | rs6493442 | *USP8* | 15 |
| rs4240910 | *PIK3CD* | 1 |  | rs9372316 | *FYN* | 6 |  | rs874941 | *USP8* | 15 |
| rs12075554 | *PIK3CD* | 1 |  | rs9384814 | *FYN* | 6 |  | rs7170543 | *USP8* | 15 |
| rs6541017 | *PIK3CD* | 1 |  | rs613276 | *MAP3K7IP2* | 6 |  | rs4318151 | *USP8* | 15 |
| rs12569008 | *PIK3CD* | 1 |  | rs583074 | *MAP3K7IP2* | 6 |  | rs3743044 | *USP8* | 15 |
| rs1622208 | *PIK3R3* | 1 |  | rs661838 | *MAP3K7IP2* | 6 |  | rs16963744 | *USP8* | 15 |
| rs1768809 | *PIK3R3* | 1 |  | rs482388 | *MAP3K7IP2* | 6 |  | rs17645290 | *USP8* | 15 |
| rs1768808 | *PIK3R3* | 1 |  | rs559052 | *MAP3K7IP2* | 6 |  | rs2272439 | *CBFA2T3* | 16 |
| rs1707322 | *PIK3R3* | 1 |  | rs483877 | *MAP3K7IP2* | 6 |  | rs2911451 | *CBFA2T3* | 16 |
| rs785512 | *PIK3R3* | 1 |  | rs504985 | *MAP3K7IP2* | 6 |  | rs522145 | *CBFA2T3* | 16 |
| rs785520 | *PIK3R3* | 1 |  | rs556335 | *MAP3K7IP2* | 6 |  | rs488251 | *CBFA2T3* | 16 |
| rs785484 | *PIK3R3* | 1 |  | rs573684 | *MAP3K7IP2* | 6 |  | rs477639 | *CBFA2T3* | 16 |
| rs9429095 | *PIK3R3* | 1 |  | rs6911411 | *MAP3K7IP2* | 6 |  | rs3809591 | *CBFA2T3* | 16 |
| rs785475 | *PIK3R3* | 1 |  | rs6570963 | *MAP3K7IP2* | 6 |  | rs538778 | *CBFA2T3* | 16 |
| rs10495562 | *ADAM17* | 2 |  | rs9498335 | *MAP3K7IP2* | 6 |  | rs9922120 | *CBFA2T3* | 16 |
| rs11891922 | *ADAM17* | 2 |  | rs12524841 | *PRL* | 6 |  | rs4782497 | *CBFA2T3* | 16 |
| rs17590882 | *ADAM17* | 2 |  | rs2744119 | *PRL* | 6 |  | rs4782496 | *CBFA2T3* | 16 |
| rs10179642 | *ADAM17* | 2 |  | rs849874 | *PRL* | 6 |  | rs13329773 | *CBFA2T3* | 16 |
| rs6432017 | *ADAM17* | 2 |  | rs1205961 | *PRL* | 6 |  | rs9932459 | *CBFA2T3* | 16 |
| rs6708544 | *CHRNA1* | 2 |  | rs849885 | *PRL* | 6 |  | rs4782494 | *CBFA2T3* | 16 |
| rs12997022 | *CHRNA1* | 2 |  | rs7739889 | *PRL* | 6 |  | rs4129789 | *CBFA2T3* | 16 |
| rs2646165 | *CHRNA1* | 2 |  | rs12210179 | *PRL* | 6 |  | rs9932354 | *CBFA2T3* | 16 |
| rs2600685 | *CHRNA1* | 2 |  | rs849876 | *PRL* | 6 |  | rs8061772 | *MAPK3* | 16 |
| rs3755485 | *CHRNA1* | 2 |  | rs1341239 | *PRL* | 6 |  | rs11865086 | *MAPK3* | 16 |
| rs3755486 | *CHRNA1* | 2 |  | rs2473122 | *PRL* | 6 |  | rs2005219 | *MAPK3* | 16 |
| rs2244340 | *CHRNA1* | 2 |  | rs11970435 | *PRL* | 6 |  | rs8063120 | *PLCG2* | 16 |
| rs935865 | *CHRNA1* | 2 |  | rs715226 | *PRL* | 6 |  | rs9937704 | *PLCG2* | 16 |
| rs1595066 | *ERBB4* | 2 |  | rs1267638 | *BRAF* | 7 |  | rs10445097 | *PLCG2* | 16 |
| rs10932374 | *ERBB4* | 2 |  | rs10487888 | *BRAF* | 7 |  | rs4328435 | *PLCG2* | 16 |
| rs1836724 | *ERBB4* | 2 |  | rs1476868 | *BRAF* | 7 |  | rs6564915 | *PLCG2* | 16 |
| rs3748960 | *ERBB4* | 2 |  | rs1267612 | *BRAF* | 7 |  | rs4254322 | *PLCG2* | 16 |
| rs3791682 | *ERBB4* | 2 |  | rs1267622 | *BRAF* | 7 |  | rs4398100 | *PLCG2* | 16 |
| rs10048757 | *ERBB4* | 2 |  | rs1267619 | *BRAF* | 7 |  | rs4580153 | *PLCG2* | 16 |
| rs16846013 | *ERBB4* | 2 |  | rs7792984 | *BRAF* | 7 |  | rs4580154 | *PLCG2* | 16 |
| rs3791691 | *ERBB4* | 2 |  | rs10269699 | *BRAF* | 7 |  | rs4243211 | *PLCG2* | 16 |
| rs3791692 | *ERBB4* | 2 |  | rs4726020 | *BRAF* | 7 |  | rs12598194 | *PLCG2* | 16 |
| rs1816532 | *ERBB4* | 2 |  | rs2072439 | *CAMK2B* | 7 |  | rs12448334 | *PLCG2* | 16 |
| rs3791696 | *ERBB4* | 2 |  | rs2009705 | *CAMK2B* | 7 |  | rs4889384 | *PLCG2* | 16 |
| rs3828243 | *ERBB4* | 2 |  | rs1127065 | *CAMK2B* | 7 |  | rs12599264 | *PLCG2* | 16 |
| rs16846100 | *ERBB4* | 2 |  | rs2075067 | *CAMK2B* | 7 |  | rs4889393 | *PLCG2* | 16 |
| rs13030304 | *ERBB4* | 2 |  | rs3757836 | *CAMK2B* | 7 |  | rs9937223 | *PLCG2* | 16 |
| rs3791703 | *ERBB4* | 2 |  | rs10441113 | *CAMK2B* | 7 |  | rs4456499 | *PLCG2* | 16 |
| rs16846133 | *ERBB4* | 2 |  | rs4526269 | *CAMK2B* | 7 |  | rs6420427 | *PLCG2* | 16 |
| rs16846140 | *ERBB4* | 2 |  | rs7796267 | *CAMK2B* | 7 |  | rs4405545 | *PLCG2* | 16 |
| rs1836717 | *ERBB4* | 2 |  | rs10230538 | *CAMK2B* | 7 |  | rs7499275 | *PLCG2* | 16 |
| rs16846161 | *ERBB4* | 2 |  | rs4724298 | *CAMK2B* | 7 |  | rs4325546 | *PLCG2* | 16 |
| rs6727581 | *ERBB4* | 2 |  | rs6968826 | *CAMK2B* | 7 |  | rs8043593 | *PLCG2* | 16 |
| rs6742399 | *ERBB4* | 2 |  | rs7804804 | *CAMK2B* | 7 |  | rs4889411 | *PLCG2* | 16 |
| rs2033647 | *ERBB4* | 2 |  | rs4410809 | *CAMK2B* | 7 |  | rs4888179 | *PLCG2* | 16 |
| rs10165449 | *ERBB4* | 2 |  | rs9918696 | *CAMK2B* | 7 |  | rs7202205 | *PLCG2* | 16 |
| rs13395652 | *ERBB4* | 2 |  | rs6962696 | *CAMK2B* | 7 |  | rs4072683 | *PLCG2* | 16 |
| rs16825005 | *ERBB4* | 2 |  | rs6974322 | *CAMK2B* | 7 |  | rs7185362 | *PLCG2* | 16 |
| rs16825008 | *ERBB4* | 2 |  | rs11764828 | *CAMK2B* | 7 |  | rs4888181 | *PLCG2* | 16 |
| rs9808028 | *ERBB4* | 2 |  | rs12535537 | *CAMK2B* | 7 |  | rs12596639 | *PLCG2* | 16 |
| rs1836715 | *ERBB4* | 2 |  | rs12535503 | *CAMK2B* | 7 |  | rs11643875 | *PLCG2* | 16 |
| rs7605095 | *ERBB4* | 2 |  | rs4947963 | *EGFR* | 7 |  | rs4243218 | *PLCG2* | 16 |
| rs4673618 | *ERBB4* | 2 |  | rs12718939 | *EGFR* | 7 |  | rs4889422 | *PLCG2* | 16 |
| rs16846200 | *ERBB4* | 2 |  | rs10488137 | *EGFR* | 7 |  | rs7342694 | *PLCG2* | 16 |
| rs16846210 | *ERBB4* | 2 |  | rs1015793 | *EGFR* | 7 |  | rs11644646 | *PLCG2* | 16 |
| rs4597456 | *ERBB4* | 2 |  | rs17172430 | *EGFR* | 7 |  | rs4997772 | *PLCG2* | 16 |
| rs2033645 | *ERBB4* | 2 |  | rs11773818 | *EGFR* | 7 |  | rs9932716 | *PLCG2* | 16 |
| rs11695594 | *ERBB4* | 2 |  | rs723527 | *EGFR* | 7 |  | rs3935743 | *PLCG2* | 16 |
| rs2371273 | *ERBB4* | 2 |  | rs1344307 | *EGFR* | 7 |  | rs7197601 | *PLCG2* | 16 |
| rs4302167 | *ERBB4* | 2 |  | rs10488140 | *EGFR* | 7 |  | rs11644436 | *PLCG2* | 16 |
| rs13383927 | *ERBB4* | 2 |  | rs17586344 | *EGFR* | 7 |  | rs12446070 | *PLCG2* | 16 |
| rs13030346 | *ERBB4* | 2 |  | rs4140770 | *EGFR* | 7 |  | rs8063355 | *PLCG2* | 16 |
| rs4439896 | *ERBB4* | 2 |  | rs984654 | *EGFR* | 7 |  | rs4133125 | *PLCG2* | 16 |
| rs4321317 | *ERBB4* | 2 |  | rs7780270 | *EGFR* | 7 |  | rs4133124 | *PLCG2* | 16 |
| rs13021324 | *ERBB4* | 2 |  | rs10244108 | *EGFR* | 7 |  | rs8055576 | *PLCG2* | 16 |
| rs9941626 | *ERBB4* | 2 |  | rs12535536 | *EGFR* | 7 |  | rs4889425 | *PLCG2* | 16 |
| rs12052288 | *ERBB4* | 2 |  | rs759169 | *EGFR* | 7 |  | rs4889426 | *PLCG2* | 16 |
| rs12053361 | *ERBB4* | 2 |  | rs11238349 | *EGFR* | 7 |  | rs4889428 | *PLCG2* | 16 |
| rs6746949 | *ERBB4* | 2 |  | rs4947490 | *EGFR* | 7 |  | rs4889432 | *PLCG2* | 16 |
| rs4278873 | *ERBB4* | 2 |  | rs11977660 | *EGFR* | 7 |  | rs4889436 | *PLCG2* | 16 |
| rs4321319 | *ERBB4* | 2 |  | rs4947491 | *EGFR* | 7 |  | rs4243221 | *PLCG2* | 16 |
| rs12621088 | *ERBB4* | 2 |  | rs2110290 | *EGFR* | 7 |  | rs4369658 | *PLCG2* | 16 |
| rs4358067 | *ERBB4* | 2 |  | rs2058502 | *EGFR* | 7 |  | rs4306504 | *PLCG2* | 16 |
| rs10932379 | *ERBB4* | 2 |  | rs759159 | *EGFR* | 7 |  | rs12448055 | *PLCG2* | 16 |
| rs6435632 | *ERBB4* | 2 |  | rs6593206 | *EGFR* | 7 |  | rs3922849 | *PLCG2* | 16 |
| rs6740117 | *ERBB4* | 2 |  | rs4947978 | *EGFR* | 7 |  | rs7203619 | *PLCG2* | 16 |
| rs12329252 | *ERBB4* | 2 |  | rs13244925 | *EGFR* | 7 |  | rs9938623 | *PLCG2* | 16 |
| rs10932380 | *ERBB4* | 2 |  | rs10488142 | *EGFR* | 7 |  | rs7201045 | *PLCG2* | 16 |
| rs17406681 | *ERBB4* | 2 |  | rs17172446 | *EGFR* | 7 |  | rs8063604 | *PLCG2* | 16 |
| rs7594456 | *ERBB4* | 2 |  | rs12666347 | *EGFR* | 7 |  | rs12446596 | *PLCG2* | 16 |
| rs6747637 | *ERBB4* | 2 |  | rs6964705 | *EGFR* | 7 |  | rs6564940 | *PLCG2* | 16 |
| rs9288433 | *ERBB4* | 2 |  | rs7801956 | *EGFR* | 7 |  | rs7187863 | *PLCG2* | 16 |
| rs10932383 | *ERBB4* | 2 |  | rs2075110 | *EGFR* | 7 |  | rs7499440 | *PLCG2* | 16 |
| rs10932384 | *ERBB4* | 2 |  | rs11506105 | *EGFR* | 7 |  | rs7500286 | *PLCG2* | 16 |
| rs4622647 | *ERBB4* | 2 |  | rs4947986 | *EGFR* | 7 |  | rs12598402 | *PLCG2* | 16 |
| rs11895509 | *ERBB4* | 2 |  | rs17172451 | *EGFR* | 7 |  | rs8043619 | *PLCG2* | 16 |
| rs953956 | *ERBB4* | 2 |  | rs3752651 | *EGFR* | 7 |  | rs3934954 | *PLCG2* | 16 |
| rs12475861 | *ERBB4* | 2 |  | rs1468727 | *EGFR* | 7 |  | rs4073828 | *PLCG2* | 16 |
| rs13421680 | *ERBB4* | 2 |  | rs11976696 | *EGFR* | 7 |  | rs3936112 | *PLCG2* | 16 |
| rs10497945 | *ERBB4* | 2 |  | rs845551 | *EGFR* | 7 |  | rs4369659 | *PLCG2* | 16 |
| rs6735769 | *ERBB4* | 2 |  | rs2241054 | *EGFR* | 7 |  | rs12918369 | *PLCG2* | 16 |
| rs12622730 | *ERBB4* | 2 |  | rs9692301 | *EGFR* | 7 |  | rs4889444 | *PLCG2* | 16 |
| rs10497947 | *ERBB4* | 2 |  | rs845552 | *EGFR* | 7 |  | rs10514519 | *PLCG2* | 16 |
| rs1009142 | *ERBB4* | 2 |  | rs845558 | *EGFR* | 7 |  | rs11862662 | *PLCG2* | 16 |
| rs10176315 | *ERBB4* | 2 |  | rs13222385 | *EGFR* | 7 |  | rs16956040 | *PLCG2* | 16 |
| rs12619171 | *ERBB4* | 2 |  | rs845561 | *EGFR* | 7 |  | rs4611452 | *PLCG2* | 16 |
| rs4672619 | *ERBB4* | 2 |  | rs2075106 | *EGFR* | 7 |  | rs4508413 | *PLCG2* | 16 |
| rs10203022 | *ERBB4* | 2 |  | rs6593210 | *EGFR* | 7 |  | rs12716928 | *PLCG2* | 16 |
| rs7556879 | *ERBB4* | 2 |  | rs845562 | *EGFR* | 7 |  | rs12921780 | *PLCG2* | 16 |
| rs7559679 | *ERBB4* | 2 |  | rs1554718 | *EGFR* | 7 |  | rs8056564 | *PLCG2* | 16 |
| rs9288436 | *ERBB4* | 2 |  | rs6970262 | *EGFR* | 7 |  | rs8055043 | *PLCG2* | 16 |
| rs1851176 | *ERBB4* | 2 |  | rs1140475 | *EGFR* | 7 |  | rs4284633 | *PLCG2* | 16 |
| rs10497948 | *ERBB4* | 2 |  | rs2293348 | *EGFR* | 7 |  | rs4312298 | *PLCG2* | 16 |
| rs7601664 | *ERBB4* | 2 |  | rs2740764 | *EGFR* | 7 |  | rs4603554 | *PLCG2* | 16 |
| rs10180037 | *ERBB4* | 2 |  | rs2293347 | *EGFR* | 7 |  | rs8063813 | *PLCG2* | 16 |
| rs1851185 | *ERBB4* | 2 |  | rs884904 | *EGFR* | 7 |  | rs8047356 | *PLCG2* | 16 |
| rs7567715 | *ERBB4* | 2 |  | rs940810 | *EGFR* | 7 |  | rs4888197 | *PLCG2* | 16 |
| rs6725181 | *ERBB4* | 2 |  | rs7334 | *EGFR* | 7 |  | rs9928191 | *PLCG2* | 16 |
| rs12476100 | *ERBB4* | 2 |  | rs940807 | *EGFR* | 7 |  | rs17793122 | *PLCG2* | 16 |
| rs1851169 | *ERBB4* | 2 |  | rs940806 | *EGFR* | 7 |  | rs4286103 | *PLCG2* | 16 |
| rs16846710 | *ERBB4* | 2 |  | rs1107616 | *EGFR* | 7 |  | rs4243226 | *PLCG2* | 16 |
| rs10207288 | *ERBB4* | 2 |  | rs11761161 | *EGFR* | 7 |  | rs9938764 | *PLCG2* | 16 |
| rs2272024 | *ERBB4* | 2 |  | rs7776830 | *EGFR* | 7 |  | rs9938835 | *PLCG2* | 16 |
| rs3817429 | *ERBB4* | 2 |  | rs1018933 | *GNAI1* | 7 |  | rs4888201 | *PLCG2* | 16 |
| rs10173674 | *ERBB4* | 2 |  | rs10236183 | *GNAI1* | 7 |  | rs9927848 | *PRKCB1* | 16 |
| rs2371276 | *ERBB4* | 2 |  | rs2428469 | *GNAI1* | 7 |  | rs12708653 | *PRKCB1* | 16 |
| rs7425448 | *ERBB4* | 2 |  | rs12706644 | *GNAI1* | 7 |  | rs7200798 | *PRKCB1* | 16 |
| rs10205553 | *ERBB4* | 2 |  | rs2523189 | *GNAI1* | 7 |  | rs2188355 | *PRKCB1* | 16 |
| rs6435660 | *ERBB4* | 2 |  | rs10274707 | *GNAI1* | 7 |  | rs8044722 | *PRKCB1* | 16 |
| rs6745717 | *ERBB4* | 2 |  | rs2714449 | *GNAI1* | 7 |  | rs12931116 | *PRKCB1* | 16 |
| rs12162287 | *ERBB4* | 2 |  | rs17806203 | *GNAI1* | 7 |  | rs11645672 | *PRKCB1* | 16 |
| rs13409739 | *ERBB4* | 2 |  | rs1468242 | *GNAI1* | 7 |  | rs1978078 | *PRKCB1* | 16 |
| rs6729362 | *ERBB4* | 2 |  | rs11980616 | *GNAI1* | 7 |  | rs7404928 | *PRKCB1* | 16 |
| rs7590438 | *ERBB4* | 2 |  | rs916905 | *GNAI1* | 7 |  | rs8063132 | *PRKCB1* | 16 |
| rs7591137 | *ERBB4* | 2 |  | rs10230368 | *GNAI1* | 7 |  | rs9924860 | *PRKCB1* | 16 |
| rs6435665 | *ERBB4* | 2 |  | rs6956455 | *GNAI1* | 7 |  | rs16972959 | *PRKCB1* | 16 |
| rs4130782 | *ERBB4* | 2 |  | rs4512317 | *GNAI1* | 7 |  | rs3785396 | *PRKCB1* | 16 |
| rs13026255 | *ERBB4* | 2 |  | rs10272499 | *GNAI1* | 7 |  | rs732798 | *PRKCB1* | 16 |
| rs13015253 | *ERBB4* | 2 |  | rs17245731 | *GNAI1* | 7 |  | rs17753569 | *PRKCB1* | 16 |
| rs12469039 | *ERBB4* | 2 |  | rs3801357 | *GNAI1* | 7 |  | rs9922316 | *PRKCB1* | 16 |
| rs6722322 | *ERBB4* | 2 |  | rs10238438 | *GNAI1* | 7 |  | rs11644907 | *PRKCB1* | 16 |
| rs6706010 | *ERBB4* | 2 |  | rs2886611 | *GNAI1* | 7 |  | rs8051531 | *PRKCB1* | 16 |
| rs7605314 | *ERBB4* | 2 |  | rs6973427 | *GNAI1* | 7 |  | rs1989647 | *PRKCB1* | 16 |
| rs7419259 | *ERBB4* | 2 |  | rs6973616 | *GNAI1* | 7 |  | rs196002 | *PRKCB1* | 16 |
| rs10932398 | *ERBB4* | 2 |  | rs2886612 | *GNAI1* | 7 |  | rs1873423 | *PRKCB1* | 16 |
| rs10932400 | *ERBB4* | 2 |  | rs2886609 | *GNAI1* | 7 |  | rs11074594 | *PRKCB1* | 16 |
| rs10432443 | *ERBB4* | 2 |  | rs193740 | *PIK3CG* | 7 |  | rs196008 | *PRKCB1* | 16 |
| rs13008370 | *ERBB4* | 2 |  | rs1724262 | *PIK3CG* | 7 |  | rs8048821 | *PRKCB1* | 16 |
| rs7423708 | *ERBB4* | 2 |  | rs1636808 | *PIK3CG* | 7 |  | rs2340988 | *PRKCB1* | 16 |
| rs7588431 | *ERBB4* | 2 |  | rs849375 | *PIK3CG* | 7 |  | rs11860367 | *PRKCB1* | 16 |
| rs6435671 | *ERBB4* | 2 |  | rs849376 | *PIK3CG* | 7 |  | rs7192816 | *PRKCB1* | 16 |
| rs6435672 | *ERBB4* | 2 |  | rs17153527 | *PIK3CG* | 7 |  | rs11859534 | *PRKCB1* | 16 |
| rs4673633 | *ERBB4* | 2 |  | rs849380 | *PIK3CG* | 7 |  | rs11074597 | *PRKCB1* | 16 |
| rs4672626 | *ERBB4* | 2 |  | rs757902 | *PIK3CG* | 7 |  | rs4788423 | *PRKCB1* | 16 |
| rs7583346 | *ERBB4* | 2 |  | rs757903 | *PIK3CG* | 7 |  | rs8055243 | *PRKCB1* | 16 |
| rs2118891 | *ERBB4* | 2 |  | rs6956373 | *PIK3CG* | 7 |  | rs17811655 | *PRKCB1* | 16 |
| rs6757087 | *ERBB4* | 2 |  | rs4727666 | *PIK3CG* | 7 |  | rs4788426 | *PRKCB1* | 16 |
| rs1371203 | *ERBB4* | 2 |  | rs1526083 | *PIK3CG* | 7 |  | rs7191360 | *PRKCB1* | 16 |
| rs1371200 | *ERBB4* | 2 |  | rs4730205 | *PIK3CG* | 7 |  | rs8054767 | *PRKCB1* | 16 |
| rs1439242 | *ERBB4* | 2 |  | rs10215499 | *PIK3CG* | 7 |  | rs11865731 | *PRKCB1* | 16 |
| rs16846917 | *ERBB4* | 2 |  | rs849412 | *PIK3CG* | 7 |  | rs17755585 | *PRKCB1* | 16 |
| rs714393 | *ERBB4* | 2 |  | rs4731117 | *WASL* | 7 |  | rs9925126 | *PRKCB1* | 16 |
| rs11892696 | *ERBB4* | 2 |  | rs2109725 | *WASL* | 7 |  | rs7202459 | *PRKCB1* | 16 |
| rs13407198 | *ERBB4* | 2 |  | rs17146253 | *WASL* | 7 |  | rs9302421 | *PRKCB1* | 16 |
| rs1159709 | *ERBB4* | 2 |  | rs10225428 | *WASL* | 7 |  | rs3785383 | *PRKCB1* | 16 |
| rs16847102 | *ERBB4* | 2 |  | rs1911145 | *WASL* | 7 |  | rs4787664 | *PRKCB1* | 16 |
| rs16847122 | *ERBB4* | 2 |  | rs3779258 | *WASL* | 7 |  | rs11074601 | *PRKCB1* | 16 |
| rs16847152 | *ERBB4* | 2 |  | rs2267875 | *WASL* | 7 |  | rs4787676 | *PRKCB1* | 16 |
| rs1439252 | *ERBB4* | 2 |  | rs4645943 | *MYC* | 8 |  | rs1976194 | *PRKCB1* | 16 |
| rs13390226 | *ERBB4* | 2 |  | rs4645956 | *MYC* | 8 |  | rs7196303 | *PRKCB1* | 16 |
| rs12986524 | *ERBB4* | 2 |  | rs7462301 | *MYC* | 8 |  | rs10852259 | *PRKCB1* | 16 |
| rs2068401 | *ERBB4* | 2 |  | rs12680047 | *MYC* | 8 |  | rs7193148 | *PRKCB1* | 16 |
| rs6727114 | *ERBB4* | 2 |  | rs4268090 | *NRG1* | 8 |  | rs7195728 | *PRKCB1* | 16 |
| rs9653337 | *ERBB4* | 2 |  | rs4452759 | *NRG1* | 8 |  | rs9929918 | *PRKCB1* | 16 |
| rs13391359 | *ERBB4* | 2 |  | rs4733263 | *NRG1* | 8 |  | rs880824 | *PRKCB1* | 16 |
| rs6435681 | *ERBB4* | 2 |  | rs4281084 | *NRG1* | 8 |  | rs405322 | *PRKCB1* | 16 |
| rs7603039 | *ERBB4* | 2 |  | rs7819063 | *NRG1* | 8 |  | rs392715 | *PRKCB1* | 16 |
| rs16847207 | *ERBB4* | 2 |  | rs13277456 | *NRG1* | 8 |  | rs11646540 | *PRKCB1* | 16 |
| rs7598440 | *ERBB4* | 2 |  | rs10113797 | *NRG1* | 8 |  | rs9937112 | *PRKCB1* | 16 |
| rs839530 | *ERBB4* | 2 |  | rs7463426 | *NRG1* | 8 |  | rs2283540 | *PRKCB1* | 16 |
| rs839525 | *ERBB4* | 2 |  | rs12677942 | *NRG1* | 8 |  | rs198182 | *PRKCB1* | 16 |
| rs839517 | *ERBB4* | 2 |  | rs4403370 | *NRG1* | 8 |  | rs1013316 | *PRKCB1* | 16 |
| rs839511 | *ERBB4* | 2 |  | rs11776643 | *NRG1* | 8 |  | rs198186 | *PRKCB1* | 16 |
| rs12623434 | *ERBB4* | 2 |  | rs13260061 | *NRG1* | 8 |  | rs169143 | *PRKCB1* | 16 |
| rs12616932 | *ERBB4* | 2 |  | rs7003184 | *NRG1* | 8 |  | rs198190 | *PRKCB1* | 16 |
| rs987320 | *ERBB4* | 2 |  | rs4733094 | *NRG1* | 8 |  | rs2238490 | *PRKCB1* | 16 |
| rs707284 | *ERBB4* | 2 |  | rs1503486 | *NRG1* | 8 |  | rs432854 | *PRKCB1* | 16 |
| rs2102996 | *ERBB4* | 2 |  | rs13270788 | *NRG1* | 8 |  | rs380932 | *PRKCB1* | 16 |
| rs12473131 | *ERBB4* | 2 |  | rs16878273 | *NRG1* | 8 |  | rs403018 | *PRKCB1* | 16 |
| rs9288445 | *ERBB4* | 2 |  | rs7830159 | *NRG1* | 8 |  | rs381901 | *PRKCB1* | 16 |
| rs1155683 | *ERBB4* | 2 |  | rs2202262 | *NRG1* | 8 |  | rs2238493 | *PRKCB1* | 16 |
| rs12987596 | *ERBB4* | 2 |  | rs2062818 | *NRG1* | 8 |  | rs1126289 | *PRKCB1* | 16 |
| rs4673646 | *ERBB4* | 2 |  | rs1354334 | *NRG1* | 8 |  | rs198207 | *PRKCB1* | 16 |
| rs954310 | *ERBB4* | 2 |  | rs1827537 | *NRG1* | 8 |  | rs198143 | *PRKCB1* | 16 |
| rs10174063 | *ERBB4* | 2 |  | rs10503892 | *NRG1* | 8 |  | rs198145 | *PRKCB1* | 16 |
| rs12618175 | *ERBB4* | 2 |  | rs1566778 | *NRG1* | 8 |  | rs562870 | *PRKCB1* | 16 |
| rs7423124 | *ERBB4* | 2 |  | rs776404 | *NRG1* | 8 |  | rs1548384 | *PRKCB1* | 16 |
| rs1879637 | *ERBB4* | 2 |  | rs2062057 | *NRG1* | 8 |  | rs2283549 | *PRKCB1* | 16 |
| rs960824 | *ERBB4* | 2 |  | rs776385 | *NRG1* | 8 |  | rs12445719 | *PRKCB1* | 16 |
| rs12464843 | *ERBB4* | 2 |  | rs2062055 | *NRG1* | 8 |  | rs198178 | *PRKCB1* | 16 |
| rs1473636 | *ERBB4* | 2 |  | rs2683771 | *NRG1* | 8 |  | rs3785378 | *PRKCB1* | 16 |
| rs1402716 | *ERBB4* | 2 |  | rs776382 | *NRG1* | 8 |  | rs198156 | *PRKCB1* | 16 |
| rs1521537 | *ERBB4* | 2 |  | rs17607869 | *NRG1* | 8 |  | rs4887934 | *WWOX* | 16 |
| rs7574462 | *ERBB4* | 2 |  | rs1481628 | *NRG1* | 8 |  | rs16947096 | *WWOX* | 16 |
| rs7607942 | *ERBB4* | 2 |  | rs1481624 | *NRG1* | 8 |  | rs7184271 | *WWOX* | 16 |
| rs1546717 | *ERBB4* | 2 |  | rs398021 | *NRG1* | 8 |  | rs1862692 | *WWOX* | 16 |
| rs1464443 | *ERBB4* | 2 |  | rs327342 | *NRG1* | 8 |  | rs8045450 | *WWOX* | 16 |
| rs6712126 | *ERBB4* | 2 |  | rs16878500 | *NRG1* | 8 |  | rs2287972 | *WWOX* | 16 |
| rs939645 | *ERBB4* | 2 |  | rs383632 | *NRG1* | 8 |  | rs16947129 | *WWOX* | 16 |
| rs4672636 | *ERBB4* | 2 |  | rs1023911 | *NRG1* | 8 |  | rs2042356 | *WWOX* | 16 |
| rs10197270 | *ERBB4* | 2 |  | rs1462891 | *NRG1* | 8 |  | rs1076514 | *WWOX* | 16 |
| rs9678219 | *ERBB4* | 2 |  | rs327371 | *NRG1* | 8 |  | rs8057015 | *WWOX* | 16 |
| rs17344051 | *ERBB4* | 2 |  | rs17683983 | *NRG1* | 8 |  | rs9319519 | *WWOX* | 16 |
| rs10497960 | *ERBB4* | 2 |  | rs327329 | *NRG1* | 8 |  | rs11644207 | *WWOX* | 16 |
| rs2371438 | *ERBB4* | 2 |  | rs1462875 | *NRG1* | 8 |  | rs7203218 | *WWOX* | 16 |
| rs10189975 | *ERBB4* | 2 |  | rs10954818 | *NRG1* | 8 |  | rs1074964 | *WWOX* | 16 |
| rs17416172 | *ERBB4* | 2 |  | rs13275646 | *NRG1* | 8 |  | rs9933156 | *WWOX* | 16 |
| rs6435689 | *ERBB4* | 2 |  | rs7827456 | *NRG1* | 8 |  | rs8062483 | *WWOX* | 16 |
| rs11887531 | *ERBB4* | 2 |  | rs10954821 | *NRG1* | 8 |  | rs3764299 | *WWOX* | 16 |
| rs11680307 | *ERBB4* | 2 |  | rs10954822 | *NRG1* | 8 |  | rs11648121 | *WWOX* | 16 |
| rs4673651 | *ERBB4* | 2 |  | rs956203 | *NRG1* | 8 |  | rs8047454 | *WWOX* | 16 |
| rs6435692 | *ERBB4* | 2 |  | rs1381866 | *NRG1* | 8 |  | rs8048266 | *WWOX* | 16 |
| rs1521658 | *ERBB4* | 2 |  | rs1462904 | *NRG1* | 8 |  | rs1079573 | *WWOX* | 16 |
| rs1357142 | *ERBB4* | 2 |  | rs16878764 | *NRG1* | 8 |  | rs16947192 | *WWOX* | 16 |
| rs6715314 | *ERBB4* | 2 |  | rs1381874 | *NRG1* | 8 |  | rs12716853 | *WWOX* | 16 |
| rs16847823 | *ERBB4* | 2 |  | rs13249578 | *NRG1* | 8 |  | rs7190335 | *WWOX* | 16 |
| rs1402766 | *ERBB4* | 2 |  | rs10503899 | *NRG1* | 8 |  | rs1922618 | *WWOX* | 16 |
| rs12621602 | *ERBB4* | 2 |  | rs11776959 | *NRG1* | 8 |  | rs4887941 | *WWOX* | 16 |
| rs1357141 | *ERBB4* | 2 |  | rs1542517 | *NRG1* | 8 |  | rs11150047 | *WWOX* | 16 |
| rs10177869 | *ERBB4* | 2 |  | rs9297186 | *NRG1* | 8 |  | rs2113160 | *WWOX* | 16 |
| rs1357139 | *ERBB4* | 2 |  | rs12680514 | *NRG1* | 8 |  | rs17572291 | *WWOX* | 16 |
| rs6755027 | *ERBB4* | 2 |  | rs1481756 | *NRG1* | 8 |  | rs11859448 | *WWOX* | 16 |
| rs6435699 | *ERBB4* | 2 |  | rs17620153 | *NRG1* | 8 |  | rs17572451 | *WWOX* | 16 |
| rs6707285 | *ERBB4* | 2 |  | rs1481737 | *NRG1* | 8 |  | rs12600238 | *WWOX* | 16 |
| rs4145960 | *ERBB4* | 2 |  | rs1481736 | *NRG1* | 8 |  | rs885734 | *WWOX* | 16 |
| rs1402769 | *ERBB4* | 2 |  | rs10087434 | *NRG1* | 8 |  | rs4887942 | *WWOX* | 16 |
| rs2049180 | *ERBB4* | 2 |  | rs2346771 | *NRG1* | 8 |  | rs12599158 | *WWOX* | 16 |
| rs10932428 | *ERBB4* | 2 |  | rs981817 | *NRG1* | 8 |  | rs4888761 | *WWOX* | 16 |
| rs1915748 | *ERBB4* | 2 |  | rs1481762 | *NRG1* | 8 |  | rs889422 | *WWOX* | 16 |
| rs939224 | *ERBB4* | 2 |  | rs1481763 | *NRG1* | 8 |  | rs889418 | *WWOX* | 16 |
| rs1357136 | *ERBB4* | 2 |  | rs6468085 | *NRG1* | 8 |  | rs7190387 | *WWOX* | 16 |
| rs10207020 | *ERBB4* | 2 |  | rs4733303 | *NRG1* | 8 |  | rs7500549 | *WWOX* | 16 |
| rs1949652 | *ERBB4* | 2 |  | rs1481742 | *NRG1* | 8 |  | rs9922503 | *WWOX* | 16 |
| rs1948875 | *ERBB4* | 2 |  | rs10093107 | *NRG1* | 8 |  | rs17652533 | *WWOX* | 16 |
| rs1521662 | *ERBB4* | 2 |  | rs4733305 | *NRG1* | 8 |  | rs3764293 | *WWOX* | 16 |
| rs6745249 | *ERBB4* | 2 |  | rs16878953 | *NRG1* | 8 |  | rs7188086 | *WWOX* | 16 |
| rs6727447 | *ERBB4* | 2 |  | rs4733306 | *NRG1* | 8 |  | rs10221047 | *WWOX* | 16 |
| rs4673657 | *ERBB4* | 2 |  | rs7001060 | *NRG1* | 8 |  | rs12918190 | *WWOX* | 16 |
| rs10205716 | *ERBB4* | 2 |  | rs10097263 | *NRG1* | 8 |  | rs16947307 | *WWOX* | 16 |
| rs1857796 | *ERBB4* | 2 |  | rs6468090 | *NRG1* | 8 |  | rs11649150 | *WWOX* | 16 |
| rs7562228 | *ERBB4* | 2 |  | rs2200047 | *NRG1* | 8 |  | rs8048681 | *WWOX* | 16 |
| rs1910866 | *ERBB4* | 2 |  | rs12549243 | *NRG1* | 8 |  | rs7197099 | *WWOX* | 16 |
| rs905883 | *ERBB4* | 2 |  | rs1383966 | *NRG1* | 8 |  | rs1079192 | *WWOX* | 16 |
| rs7601515 | *ERBB4* | 2 |  | rs11782156 | *NRG1* | 8 |  | rs7193919 | *WWOX* | 16 |
| rs1505366 | *ERBB4* | 2 |  | rs1351510 | *NRG1* | 8 |  | rs7196681 | *WWOX* | 16 |
| rs7562445 | *ERBB4* | 2 |  | rs989465 | *NRG1* | 8 |  | rs4508428 | *WWOX* | 16 |
| rs1505374 | *ERBB4* | 2 |  | rs10096233 | *NRG1* | 8 |  | rs6564520 | *WWOX* | 16 |
| rs1394782 | *ERBB4* | 2 |  | rs1481728 | *NRG1* | 8 |  | rs9926888 | *WWOX* | 16 |
| rs1394784 | *ERBB4* | 2 |  | rs6992010 | *NRG1* | 8 |  | rs11150053 | *WWOX* | 16 |
| rs12476049 | *ERBB4* | 2 |  | rs4733313 | *NRG1* | 8 |  | rs4888773 | *WWOX* | 16 |
| rs1394786 | *ERBB4* | 2 |  | rs1531746 | *NRG1* | 8 |  | rs4887950 | *WWOX* | 16 |
| rs1394788 | *ERBB4* | 2 |  | rs900102 | *NRG1* | 8 |  | rs4427816 | *WWOX* | 16 |
| rs1505359 | *ERBB4* | 2 |  | rs17624670 | *NRG1* | 8 |  | rs4267317 | *WWOX* | 16 |
| rs2371573 | *ERBB4* | 2 |  | rs1481758 | *NRG1* | 8 |  | rs7199997 | *WWOX* | 16 |
| rs10192514 | *ERBB4* | 2 |  | rs7822564 | *NRG1* | 8 |  | rs12596093 | *WWOX* | 16 |
| rs2888089 | *ERBB4* | 2 |  | rs16879088 | *NRG1* | 8 |  | rs9922494 | *WWOX* | 16 |
| rs13018908 | *ERBB4* | 2 |  | rs1487146 | *NRG1* | 8 |  | rs736918 | *WWOX* | 16 |
| rs17347530 | *ERBB4* | 2 |  | rs16879099 | *NRG1* | 8 |  | rs2077576 | *WWOX* | 16 |
| rs17347606 | *ERBB4* | 2 |  | rs4733117 | *NRG1* | 8 |  | rs4074541 | *WWOX* | 16 |
| rs1394799 | *ERBB4* | 2 |  | rs16879107 | *NRG1* | 8 |  | rs13337194 | *WWOX* | 16 |
| rs6734836 | *ERBB4* | 2 |  | rs10503902 | *NRG1* | 8 |  | rs7199623 | *WWOX* | 16 |
| rs4511675 | *ERBB4* | 2 |  | rs901561 | *NRG1* | 8 |  | rs9635572 | *WWOX* | 16 |
| rs10186681 | *ERBB4* | 2 |  | rs7818821 | *NRG1* | 8 |  | rs11866209 | *WWOX* | 16 |
| rs1394781 | *ERBB4* | 2 |  | rs6468095 | *NRG1* | 8 |  | rs9922483 | *WWOX* | 16 |
| rs13383860 | *ERBB4* | 2 |  | rs10503904 | *NRG1* | 8 |  | rs7192196 | *WWOX* | 16 |
| rs4627509 | *ERBB4* | 2 |  | rs716144 | *NRG1* | 8 |  | rs10871349 | *WWOX* | 16 |
| rs12105481 | *ERBB4* | 2 |  | rs10503905 | *NRG1* | 8 |  | rs3924959 | *WWOX* | 16 |
| rs1394796 | *ERBB4* | 2 |  | rs4317533 | *NRG1* | 8 |  | rs8052110 | *WWOX* | 16 |
| rs4673664 | *ERBB4* | 2 |  | rs10503907 | *NRG1* | 8 |  | rs9937072 | *WWOX* | 16 |
| rs6757140 | *ERBB4* | 2 |  | rs6999977 | *NRG1* | 8 |  | rs4493047 | *WWOX* | 16 |
| rs13400881 | *ERBB4* | 2 |  | rs1545961 | *NRG1* | 8 |  | rs4624193 | *WWOX* | 16 |
| rs13417523 | *ERBB4* | 2 |  | rs1386440 | *NRG1* | 8 |  | rs8064138 | *WWOX* | 16 |
| rs13383863 | *ERBB4* | 2 |  | rs1948098 | *NRG1* | 8 |  | rs7201630 | *WWOX* | 16 |
| rs13393577 | *ERBB4* | 2 |  | rs7822917 | *NRG1* | 8 |  | rs4328452 | *WWOX* | 16 |
| rs1505376 | *ERBB4* | 2 |  | rs16879142 | *NRG1* | 8 |  | rs12373085 | *WWOX* | 16 |
| rs17259208 | *ERBB4* | 2 |  | rs1487155 | *NRG1* | 8 |  | rs4243156 | *WWOX* | 16 |
| rs17325821 | *ERBB4* | 2 |  | rs1685101 | *NRG1* | 8 |  | rs7186569 | *WWOX* | 16 |
| rs6735807 | *ERBB4* | 2 |  | rs1685103 | *NRG1* | 8 |  | rs7184686 | *WWOX* | 16 |
| rs1482378 | *ERBB4* | 2 |  | rs970998 | *NRG1* | 8 |  | rs9928827 | *WWOX* | 16 |
| rs9283525 | *ERBB4* | 2 |  | rs1565031 | *NRG1* | 8 |  | rs8062393 | *WWOX* | 16 |
| rs7571561 | *ERBB4* | 2 |  | rs10091429 | *NRG1* | 8 |  | rs4502225 | *WWOX* | 16 |
| rs1026882 | *ERBB4* | 2 |  | rs12676641 | *NRG1* | 8 |  | rs6564538 | *WWOX* | 16 |
| rs1351593 | *ERBB4* | 2 |  | rs1714420 | *NRG1* | 8 |  | rs9938424 | *WWOX* | 16 |
| rs6435711 | *ERBB4* | 2 |  | rs6992907 | *NRG1* | 8 |  | rs7198400 | *WWOX* | 16 |
| rs7599312 | *ERBB4* | 2 |  | rs2347504 | *NRG1* | 8 |  | rs6564559 | *WWOX* | 16 |
| rs17262541 | *ERBB4* | 2 |  | rs10503913 | *NRG1* | 8 |  | rs3764342 | *WWOX* | 16 |
| rs4556998 | *NCK2* | 2 |  | rs2347505 | *NRG1* | 8 |  | rs11863844 | *WWOX* | 16 |
| rs11899929 | *NCK2* | 2 |  | rs2347506 | *NRG1* | 8 |  | rs7189823 | *WWOX* | 16 |
| rs997522 | *NCK2* | 2 |  | rs2347507 | *NRG1* | 8 |  | rs7204203 | *WWOX* | 16 |
| rs12987484 | *NCK2* | 2 |  | rs2347509 | *NRG1* | 8 |  | rs8054130 | *WWOX* | 16 |
| rs10496402 | *NCK2* | 2 |  | rs4373483 | *NRG1* | 8 |  | rs1397931 | *WWOX* | 16 |
| rs1114882 | *NCK2* | 2 |  | rs939077 | *NRG1* | 8 |  | rs2667657 | *WWOX* | 16 |
| rs4851843 | *NCK2* | 2 |  | rs12676409 | *NRG1* | 8 |  | rs2941934 | *WWOX* | 16 |
| rs4851848 | *NCK2* | 2 |  | rs16879327 | *NRG1* | 8 |  | rs8062920 | *WWOX* | 16 |
| rs4851857 | *NCK2* | 2 |  | rs12550153 | *NRG1* | 8 |  | rs2667543 | *WWOX* | 16 |
| rs12465089 | *NCK2* | 2 |  | rs3847131 | *NRG1* | 8 |  | rs2738679 | *WWOX* | 16 |
| rs12470376 | *NCK2* | 2 |  | rs9918832 | *NRG1* | 8 |  | rs2667552 | *WWOX* | 16 |
| rs1897169 | *NCK2* | 2 |  | rs9297191 | *NRG1* | 8 |  | rs11643648 | *WWOX* | 16 |
| rs7571132 | *NCK2* | 2 |  | rs10503915 | *NRG1* | 8 |  | rs2738696 | *WWOX* | 16 |
| rs6732799 | *NCK2* | 2 |  | rs16879366 | *NRG1* | 8 |  | rs1540757 | *WWOX* | 16 |
| rs7588033 | *NCK2* | 2 |  | rs11782671 | *NRG1* | 8 |  | rs2738697 | *WWOX* | 16 |
| rs9646959 | *NCK2* | 2 |  | rs2347501 | *NRG1* | 8 |  | rs16947728 | *WWOX* | 16 |
| rs2047699 | *NCK2* | 2 |  | rs4584111 | *NRG1* | 8 |  | rs12716855 | *WWOX* | 16 |
| rs12464067 | *NCK2* | 2 |  | rs4458837 | *NRG1* | 8 |  | rs4888810 | *WWOX* | 16 |
| rs12995333 | *NCK2* | 2 |  | rs10103930 | *NRG1* | 8 |  | rs9931978 | *WWOX* | 16 |
| rs4851870 | *NCK2* | 2 |  | rs6996957 | *NRG1* | 8 |  | rs3419 | *WWOX* | 16 |
| rs1465639 | *NCK2* | 2 |  | rs2347497 | *NRG1* | 8 |  | rs17639042 | *WWOX* | 16 |
| rs6741172 | *NCK2* | 2 |  | rs17716295 | *NRG1* | 8 |  | rs1397927 | *WWOX* | 16 |
| rs2163350 | *NCK2* | 2 |  | rs6985581 | *NRG1* | 8 |  | rs8055733 | *WWOX* | 16 |
| rs2163349 | *NCK2* | 2 |  | rs10954845 | *NRG1* | 8 |  | rs4257220 | *WWOX* | 16 |
| rs6543342 | *NCK2* | 2 |  | rs16879442 | *NRG1* | 8 |  | rs2667609 | *WWOX* | 16 |
| rs10180659 | *NCK2* | 2 |  | rs10096770 | *NRG1* | 8 |  | rs16947885 | *WWOX* | 16 |
| rs3769504 | *NCK2* | 2 |  | rs7844597 | *NRG1* | 8 |  | rs4319778 | *WWOX* | 16 |
| rs3754806 | *NCK2* | 2 |  | rs12676463 | *NRG1* | 8 |  | rs9928512 | *WWOX* | 16 |
| rs879900 | *NCK2* | 2 |  | rs17645111 | *NRG1* | 8 |  | rs11645676 | *WWOX* | 16 |
| rs6740343 | *NCK2* | 2 |  | rs4733342 | *NRG1* | 8 |  | rs8051498 | *WWOX* | 16 |
| rs938662 | *PRKCE* | 2 |  | rs4733122 | *NRG1* | 8 |  | rs2859642 | *WWOX* | 16 |
| rs2344662 | *PRKCE* | 2 |  | rs10503918 | *NRG1* | 8 |  | rs2738498 | *WWOX* | 16 |
| rs614281 | *PRKCE* | 2 |  | rs17645417 | *NRG1* | 8 |  | rs2667627 | *WWOX* | 16 |
| rs6722418 | *PRKCE* | 2 |  | rs7013361 | *NRG1* | 8 |  | rs1105314 | *WWOX* | 16 |
| rs546639 | *PRKCE* | 2 |  | rs6651144 | *NRG1* | 8 |  | rs17777700 | *WWOX* | 16 |
| rs608139 | *PRKCE* | 2 |  | rs10107065 | *NRG1* | 8 |  | rs2667646 | *WWOX* | 16 |
| rs666214 | *PRKCE* | 2 |  | rs12334435 | *NRG1* | 8 |  | rs2667650 | *WWOX* | 16 |
| rs17033965 | *PRKCE* | 2 |  | rs4489283 | *NRG1* | 8 |  | rs10514438 | *WWOX* | 16 |
| rs3731949 | *PRKCE* | 2 |  | rs7826312 | *NRG1* | 8 |  | rs2458029 | *WWOX* | 16 |
| rs528349 | *PRKCE* | 2 |  | rs7000590 | *NRG1* | 8 |  | rs7203676 | *WWOX* | 16 |
| rs585156 | *PRKCE* | 2 |  | rs7005606 | *NRG1* | 8 |  | rs11640157 | *WWOX* | 16 |
| rs556650 | *PRKCE* | 2 |  | rs2439312 | *NRG1* | 8 |  | rs9928690 | *WWOX* | 16 |
| rs10207776 | *PRKCE* | 2 |  | rs11776203 | *NRG1* | 8 |  | rs11642089 | *WWOX* | 16 |
| rs2204204 | *PRKCE* | 2 |  | rs10503920 | *NRG1* | 8 |  | rs3853362 | *WWOX* | 16 |
| rs12185636 | *PRKCE* | 2 |  | rs4733362 | *NRG1* | 8 |  | rs2346007 | *WWOX* | 16 |
| rs506134 | *PRKCE* | 2 |  | rs2439300 | *NRG1* | 8 |  | rs2943768 | *WWOX* | 16 |
| rs10490342 | *PRKCE* | 2 |  | rs17720837 | *NRG1* | 8 |  | rs7197903 | *WWOX* | 16 |
| rs542413 | *PRKCE* | 2 |  | rs17664708 | *NRG1* | 8 |  | rs9928840 | *WWOX* | 16 |
| rs10490341 | *PRKCE* | 2 |  | rs2466067 | *NRG1* | 8 |  | rs11862167 | *WWOX* | 16 |
| rs628877 | *PRKCE* | 2 |  | rs2466066 | *NRG1* | 8 |  | rs7184757 | *WWOX* | 16 |
| rs629772 | *PRKCE* | 2 |  | rs10954859 | *NRG1* | 8 |  | rs1828518 | *WWOX* | 16 |
| rs656823 | *PRKCE* | 2 |  | rs4602844 | *NRG1* | 8 |  | rs2667542 | *WWOX* | 16 |
| rs11674329 | *PRKCE* | 2 |  | rs3924999 | *NRG1* | 8 |  | rs1126185 | *WWOX* | 16 |
| rs483404 | *PRKCE* | 2 |  | rs10954863 | *NRG1* | 8 |  | rs2738555 | *WWOX* | 16 |
| rs6746467 | *PRKCE* | 2 |  | rs2439281 | *NRG1* | 8 |  | rs3866638 | *WWOX* | 16 |
| rs642200 | *PRKCE* | 2 |  | rs2466059 | *NRG1* | 8 |  | rs4887970 | *WWOX* | 16 |
| rs1464573 | *PRKCE* | 2 |  | rs12547858 | *NRG1* | 8 |  | rs2738571 | *WWOX* | 16 |
| rs1522987 | *PRKCE* | 2 |  | rs2439273 | *NRG1* | 8 |  | rs2548861 | *WWOX* | 16 |
| rs2176348 | *PRKCE* | 2 |  | rs10095694 | *NRG1* | 8 |  | rs2738576 | *WWOX* | 16 |
| rs6725257 | *PRKCE* | 2 |  | rs4531002 | *NRG1* | 8 |  | rs7200698 | *WWOX* | 16 |
| rs7577664 | *PRKCE* | 2 |  | rs11989919 | *NRG1* | 8 |  | rs1079323 | *WWOX* | 16 |
| rs13033787 | *PRKCE* | 2 |  | rs2466058 | *NRG1* | 8 |  | rs7196183 | *WWOX* | 16 |
| rs4557033 | *PRKCE* | 2 |  | rs2976521 | *NRG1* | 8 |  | rs1107455 | *WWOX* | 16 |
| rs7558633 | *PRKCE* | 2 |  | rs13259346 | *NRG1* | 8 |  | rs2550626 | *WWOX* | 16 |
| rs952331 | *PRKCE* | 2 |  | rs2466051 | *NRG1* | 8 |  | rs7190546 | *WWOX* | 16 |
| rs2176347 | *PRKCE* | 2 |  | rs2466048 | *NRG1* | 8 |  | rs2161620 | *WWOX* | 16 |
| rs1878384 | *PRKCE* | 2 |  | rs2466046 | *NRG1* | 8 |  | rs10514440 | *WWOX* | 16 |
| rs12995580 | *PRKCE* | 2 |  | rs2439321 | *NRG1* | 8 |  | rs2548843 | *WWOX* | 16 |
| rs17034091 | *PRKCE* | 2 |  | rs2919390 | *NRG1* | 8 |  | rs2113120 | *WWOX* | 16 |
| rs6716268 | *PRKCE* | 2 |  | rs2954045 | *NRG1* | 8 |  | rs1125671 | *WWOX* | 16 |
| rs11125030 | *PRKCE* | 2 |  | rs12546380 | *NRG1* | 8 |  | rs1109876 | *WWOX* | 16 |
| rs11890554 | *PRKCE* | 2 |  | rs6468122 | *NRG1* | 8 |  | rs1364295 | *WWOX* | 16 |
| rs4953245 | *PRKCE* | 2 |  | rs10503927 | *NRG1* | 8 |  | rs2548836 | *WWOX* | 16 |
| rs11125031 | *PRKCE* | 2 |  | rs6988339 | *NRG1* | 8 |  | rs16948286 | *WWOX* | 16 |
| rs1533476 | *PRKCE* | 2 |  | rs2975498 | *NRG1* | 8 |  | rs2081174 | *WWOX* | 16 |
| rs940052 | *PRKCE* | 2 |  | rs2919384 | *NRG1* | 8 |  | rs8054120 | *WWOX* | 16 |
| rs6716622 | *PRKCE* | 2 |  | rs2919381 | *NRG1* | 8 |  | rs2194340 | *WWOX* | 16 |
| rs884399 | *PRKCE* | 2 |  | rs16879809 | *NRG1* | 8 |  | rs17795223 | *WWOX* | 16 |
| rs1468042 | *PRKCE* | 2 |  | rs2976525 | *NRG1* | 8 |  | rs2550655 | *WWOX* | 16 |
| rs11684301 | *PRKCE* | 2 |  | rs16879814 | *NRG1* | 8 |  | rs9929802 | *WWOX* | 16 |
| rs7582320 | *PRKCE* | 2 |  | rs2919377 | *NRG1* | 8 |  | rs2738627 | *WWOX* | 16 |
| rs4953249 | *PRKCE* | 2 |  | rs2976527 | *NRG1* | 8 |  | rs12926298 | *WWOX* | 16 |
| rs4953251 | *PRKCE* | 2 |  | rs2919374 | *NRG1* | 8 |  | rs3751881 | *WWOX* | 16 |
| rs13404973 | *PRKCE* | 2 |  | rs17669781 | *NRG1* | 8 |  | rs3751882 | *WWOX* | 16 |
| rs4446102 | *PRKCE* | 2 |  | rs16879886 | *NRG1* | 8 |  | rs7185340 | *WWOX* | 16 |
| rs6758953 | *PRKCE* | 2 |  | rs2919389 | *NRG1* | 8 |  | rs6564576 | *WWOX* | 16 |
| rs13016569 | *PRKCE* | 2 |  | rs12682440 | *NRG1* | 8 |  | rs4888826 | *WWOX* | 16 |
| rs4952773 | *PRKCE* | 2 |  | rs10103750 | *NRG1* | 8 |  | rs4887974 | *WWOX* | 16 |
| rs4953255 | *PRKCE* | 2 |  | rs7007436 | *NRG1* | 8 |  | rs1110556 | *WWOX* | 16 |
| rs4264601 | *PRKCE* | 2 |  | rs16879922 | *NRG1* | 8 |  | rs4888831 | *WWOX* | 16 |
| rs10191412 | *PRKCE* | 2 |  | rs6982890 | *NRG1* | 8 |  | rs1110560 | *WWOX* | 16 |
| rs4074080 | *PRKCE* | 2 |  | rs7005288 | *NRG1* | 8 |  | rs9925796 | *WWOX* | 16 |
| rs7558342 | *PRKCE* | 2 |  | rs6992642 | *NRG1* | 8 |  | rs4887979 | *WWOX* | 16 |
| rs11898074 | *PRKCE* | 2 |  | rs3735781 | *NRG1* | 8 |  | rs7199110 | *WWOX* | 16 |
| rs11898209 | *PRKCE* | 2 |  | rs3735782 | *NRG1* | 8 |  | rs1110554 | *WWOX* | 16 |
| rs4952774 | *PRKCE* | 2 |  | rs11780123 | *NRG1* | 8 |  | rs7201295 | *WWOX* | 16 |
| rs4952775 | *PRKCE* | 2 |  | rs10108278 | *PTK2* | 8 |  | rs7199334 | *WWOX* | 16 |
| rs6720975 | *PRKCE* | 2 |  | rs7016497 | *PTK2* | 8 |  | rs7205435 | *WWOX* | 16 |
| rs6743144 | *PRKCE* | 2 |  | rs10283368 | *PTK2* | 8 |  | rs9929519 | *WWOX* | 16 |
| rs4953260 | *PRKCE* | 2 |  | rs4413752 | *PTK2* | 8 |  | rs17642004 | *WWOX* | 16 |
| rs4953262 | *PRKCE* | 2 |  | rs10089107 | *PTK2* | 8 |  | rs1477413 | *WWOX* | 16 |
| rs3886870 | *PRKCE* | 2 |  | rs11785444 | *PTK2* | 8 |  | rs9922434 | *WWOX* | 16 |
| rs7596891 | *PRKCE* | 2 |  | rs4961287 | *PTK2* | 8 |  | rs9932331 | *WWOX* | 16 |
| rs935672 | *PRKCE* | 2 |  | rs4596627 | *PTK2* | 8 |  | rs7190803 | *WWOX* | 16 |
| rs11125034 | *PRKCE* | 2 |  | rs10109684 | *PTK2* | 8 |  | rs16948473 | *WWOX* | 16 |
| rs7559522 | *PRKCE* | 2 |  | rs10100025 | *PTK2* | 8 |  | rs12921046 | *WWOX* | 16 |
| rs7576304 | *PRKCE* | 2 |  | rs11166995 | *PTK2* | 8 |  | rs2550593 | *WWOX* | 16 |
| rs10179954 | *PRKCE* | 2 |  | rs7839832 | *PTK2* | 8 |  | rs2737305 | *WWOX* | 16 |
| rs4953266 | *PRKCE* | 2 |  | rs11997161 | *PTK2* | 8 |  | rs7195449 | *WWOX* | 16 |
| rs10175198 | *PRKCE* | 2 |  | rs6994744 | *PTK2* | 8 |  | rs17723058 | *WWOX* | 16 |
| rs10175158 | *PRKCE* | 2 |  | rs4961289 | *PTK2* | 8 |  | rs2550598 | *WWOX* | 16 |
| rs935661 | *PRKCE* | 2 |  | rs7831543 | *PTK2* | 8 |  | rs7185036 | *WWOX* | 16 |
| rs10865208 | *PRKCE* | 2 |  | rs4327896 | *PTK2* | 8 |  | rs1110559 | *WWOX* | 16 |
| rs4952779 | *PRKCE* | 2 |  | rs10087782 | *PTK2* | 8 |  | rs9936415 | *WWOX* | 16 |
| rs935656 | *PRKCE* | 2 |  | rs1868280 | *PTK2* | 8 |  | rs9939233 | *WWOX* | 16 |
| rs6732900 | *PRKCE* | 2 |  | rs1868279 | *PTK2* | 8 |  | rs11863800 | *WWOX* | 16 |
| rs6761327 | *PRKCE* | 2 |  | rs11991796 | *PTK2* | 8 |  | rs2293902 | *WWOX* | 16 |
| rs750210 | *PRKCE* | 2 |  | rs4961237 | *PTK2* | 8 |  | rs1123882 | *WWOX* | 16 |
| rs4953268 | *PRKCE* | 2 |  | rs10111852 | *PTK2* | 8 |  | rs6564594 | *WWOX* | 16 |
| rs6761356 | *PRKCE* | 2 |  | rs12544802 | *PTK2* | 8 |  | rs4145519 | *WWOX* | 16 |
| rs4953270 | *PRKCE* | 2 |  | rs13270490 | *PTK2* | 8 |  | rs1124595 | *WWOX* | 16 |
| rs6719779 | *PRKCE* | 2 |  | rs7005909 | *PTK2* | 8 |  | rs3946180 | *WWOX* | 16 |
| rs7561544 | *PRKCE* | 2 |  | rs12544404 | *PTK2* | 8 |  | rs8052915 | *WWOX* | 16 |
| rs6756452 | *PRKCE* | 2 |  | rs306953 | *PTK2* | 8 |  | rs1530 | *WWOX* | 16 |
| rs6760363 | *PRKCE* | 2 |  | rs12545416 | *PTK2* | 8 |  | rs6564596 | *WWOX* | 16 |
| rs7581914 | *PRKCE* | 2 |  | rs7840147 | *PTK2* | 8 |  | rs12445110 | *WWOX* | 16 |
| rs4953274 | *PRKCE* | 2 |  | rs11167020 | *PTK2* | 8 |  | rs7196220 | *WWOX* | 16 |
| rs4953276 | *PRKCE* | 2 |  | rs11167021 | *PTK2* | 8 |  | rs7201888 | *WWOX* | 16 |
| rs1947195 | *PRKCE* | 2 |  | rs10956802 | *WWP1* | 8 |  | rs7199947 | *WWOX* | 16 |
| rs4953277 | *PRKCE* | 2 |  | rs7830128 | *WWP1* | 8 |  | rs12446194 | *WWOX* | 16 |
| rs935653 | *PRKCE* | 2 |  | rs6471314 | *WWP1* | 8 |  | rs2037961 | *WWOX* | 16 |
| rs3911797 | *PRKCE* | 2 |  | rs12155981 | *WWP1* | 8 |  | rs9936829 | *WWOX* | 16 |
| rs10189339 | *PRKCE* | 2 |  | rs10100368 | *WWP1* | 8 |  | rs7184196 | *WWOX* | 16 |
| rs4952787 | *PRKCE* | 2 |  | rs2269337 | *ABL1* | 9 |  | rs6564600 | *WWOX* | 16 |
| rs11125038 | *PRKCE* | 2 |  | rs2583839 | *ABL1* | 9 |  | rs8048851 | *WWOX* | 16 |
| rs11125039 | *PRKCE* | 2 |  | rs4740359 | *ABL1* | 9 |  | rs9941131 | *WWOX* | 16 |
| rs1966813 | *PRKCE* | 2 |  | rs10751508 | *ABL1* | 9 |  | rs6564605 | *WWOX* | 16 |
| rs7585985 | *PRKCE* | 2 |  | rs2791731 | *ABL1* | 9 |  | rs1554983 | *WWOX* | 16 |
| rs2122110 | *PRKCE* | 2 |  | rs4740363 | *ABL1* | 9 |  | rs7185014 | *WWOX* | 16 |
| rs4245804 | *PRKCE* | 2 |  | rs2855169 | *ABL1* | 9 |  | rs13332126 | *WWOX* | 16 |
| rs2345177 | *PRKCE* | 2 |  | rs2583836 | *ABL1* | 9 |  | rs7194700 | *WWOX* | 16 |
| rs2881068 | *PRKCE* | 2 |  | rs4740203 | *ABL1* | 9 |  | rs4888860 | *WWOX* | 16 |
| rs13400225 | *PRKCE* | 2 |  | rs2855171 | *ABL1* | 9 |  | rs7405423 | *WWOX* | 16 |
| rs11125041 | *PRKCE* | 2 |  | rs4990084 | *ABL1* | 9 |  | rs6420411 | *WWOX* | 16 |
| rs872288 | *PRKCE* | 2 |  | rs2251403 | *ABL1* | 9 |  | rs11643930 | *WWOX* | 16 |
| rs4953288 | *PRKCE* | 2 |  | rs2855179 | *ABL1* | 9 |  | rs4145518 | *WWOX* | 16 |
| rs10187908 | *PRKCE* | 2 |  | rs7026988 | *ABL1* | 9 |  | rs1125814 | *WWOX* | 16 |
| rs1868388 | *PRKCE* | 2 |  | rs10901285 | *ABL1* | 9 |  | rs6564609 | *WWOX* | 16 |
| rs747032 | *PRKCE* | 2 |  | rs11244147 | *ABL1* | 9 |  | rs4888867 | *WWOX* | 16 |
| rs13036100 | *PRKCE* | 2 |  | rs10901291 | *ABL1* | 9 |  | rs8054190 | *WWOX* | 16 |
| rs12999695 | *PRKCE* | 2 |  | rs3824400 | *ABL1* | 9 |  | rs2737290 | *WWOX* | 16 |
| rs10206343 | *PRKCE* | 2 |  | rs11244164 | *ABL1* | 9 |  | rs2737289 | *WWOX* | 16 |
| rs7578578 | *PRKCE* | 2 |  | rs3780282 | *ABL1* | 9 |  | rs7197238 | *WWOX* | 16 |
| rs2595191 | *PRKCE* | 2 |  | rs12005009 | *ABL1* | 9 |  | rs2178952 | *WWOX* | 16 |
| rs2711292 | *PRKCE* | 2 |  | rs3808814 | *ABL1* | 9 |  | rs9926713 | *WWOX* | 16 |
| rs2029087 | *PRKCE* | 2 |  | rs7034278 | *ABL1* | 9 |  | rs2737280 | *WWOX* | 16 |
| rs2711299 | *PRKCE* | 2 |  | rs7040543 | *ABL1* | 9 |  | rs10492911 | *WWOX* | 16 |
| rs2711295 | *PRKCE* | 2 |  | rs7865017 | *ABL1* | 9 |  | rs2656630 | *WWOX* | 16 |
| rs13023787 | *PRKCE* | 2 |  | rs7853654 | *ABL1* | 9 |  | rs1394572 | *WWOX* | 16 |
| rs11903566 | *PRKCE* | 2 |  | rs10760165 | *GSN* | 9 |  | rs2656620 | *WWOX* | 16 |
| rs12471357 | *PRKCE* | 2 |  | rs10760167 | *GSN* | 9 |  | rs9923225 | *WWOX* | 16 |
| rs1463162 | *PRKCE* | 2 |  | rs878691 | *GSN* | 9 |  | rs4435265 | *WWOX* | 16 |
| rs4953292 | *PRKCE* | 2 |  | rs7046030 | *GSN* | 9 |  | rs8061768 | *WWOX* | 16 |
| rs2595202 | *PRKCE* | 2 |  | rs1078305 | *GSN* | 9 |  | rs12918342 | *WWOX* | 16 |
| rs2595203 | *PRKCE* | 2 |  | rs10760169 | *GSN* | 9 |  | rs10492909 | *WWOX* | 16 |
| rs6706140 | *PRKCE* | 2 |  | rs7028970 | *GSN* | 9 |  | rs11646646 | *WWOX* | 16 |
| rs2595204 | *PRKCE* | 2 |  | rs306784 | *GSN* | 9 |  | rs2174403 | *WWOX* | 16 |
| rs2595205 | *PRKCE* | 2 |  | rs306761 | *GSN* | 9 |  | rs11150110 | *WWOX* | 16 |
| rs7607360 | *PRKCE* | 2 |  | rs306772 | *GSN* | 9 |  | rs7192392 | *WWOX* | 16 |
| rs11125043 | *PRKCE* | 2 |  | rs7850675 | *JAK2* | 9 |  | rs9939686 | *WWOX* | 16 |
| rs1562651 | *PRKCE* | 2 |  | rs1887426 | *JAK2* | 9 |  | rs11864605 | *WWOX* | 16 |
| rs1562653 | *PRKCE* | 2 |  | rs7870381 | *JAK2* | 9 |  | rs16948787 | *WWOX* | 16 |
| rs6751933 | *PRKCE* | 2 |  | rs7862852 | *JAK2* | 9 |  | rs954811 | *WWOX* | 16 |
| rs753572 | *PRKCE* | 2 |  | rs1327495 | *JAK2* | 9 |  | rs17707630 | *WWOX* | 16 |
| rs12464563 | *PRKCE* | 2 |  | rs1887427 | *JAK2* | 9 |  | rs16948856 | *WWOX* | 16 |
| rs2595211 | *PRKCE* | 2 |  | rs10758669 | *JAK2* | 9 |  | rs8049613 | *WWOX* | 16 |
| rs11677077 | *PRKCE* | 2 |  | rs2274471 | *JAK2* | 9 |  | rs2221434 | *WWOX* | 16 |
| rs6705717 | *PRKCE* | 2 |  | rs7849191 | *JAK2* | 9 |  | rs9923451 | *WWOX* | 16 |
| rs7594827 | *PRKCE* | 2 |  | rs12347727 | *JAK2* | 9 |  | rs2881483 | *WWOX* | 16 |
| rs2245633 | *PRKCE* | 2 |  | rs4372063 | *JAK2* | 9 |  | rs1018159 | *WWOX* | 16 |
| rs2595214 | *PRKCE* | 2 |  | rs7030260 | *JAK2* | 9 |  | rs1510218 | *WWOX* | 16 |
| rs2122112 | *PRKCE* | 2 |  | rs10815144 | *JAK2* | 9 |  | rs2134993 | *WWOX* | 16 |
| rs2711302 | *PRKCE* | 2 |  | rs11998810 | *JAK2* | 9 |  | rs11859772 | *WWOX* | 16 |
| rs4952796 | *PRKCE* | 2 |  | rs7851556 | *JAK2* | 9 |  | rs12924818 | *WWOX* | 16 |
| rs6755308 | *PRKCE* | 2 |  | rs1328917 | *JAK2* | 9 |  | rs4888881 | *WWOX* | 16 |
| rs2595221 | *PRKCE* | 2 |  | rs1536800 | *JAK2* | 9 |  | rs8047671 | *WWOX* | 16 |
| rs13017044 | *PRKCE* | 2 |  | rs10815149 | *JAK2* | 9 |  | rs7203016 | *WWOX* | 16 |
| rs3738900 | *PRKCE* | 2 |  | rs10974947 | *JAK2* | 9 |  | rs12596126 | *WWOX* | 16 |
| rs3820733 | *PRKCE* | 2 |  | rs1410779 | *JAK2* | 9 |  | rs13337252 | *WWOX* | 16 |
| rs3738898 | *PRKCE* | 2 |  | rs2031904 | *JAK2* | 9 |  | rs13333229 | *WWOX* | 16 |
| rs6743504 | *PRKCE* | 2 |  | rs3780373 | *JAK2* | 9 |  | rs6564626 | *WWOX* | 16 |
| rs7349397 | *PRKCE* | 2 |  | rs3780374 | *JAK2* | 9 |  | rs7195069 | *WWOX* | 16 |
| rs6748835 | *PRKCE* | 2 |  | rs3780381 | *JAK2* | 9 |  | rs4888884 | *WWOX* | 16 |
| rs13024806 | *PRKCE* | 2 |  | rs9314668 | *SHC3* | 9 |  | rs4887995 | *WWOX* | 16 |
| rs11903923 | *PRKCE* | 2 |  | rs3750396 | *SHC3* | 9 |  | rs1995549 | *WWOX* | 16 |
| rs1470598 | *PRKCE* | 2 |  | rs9410438 | *SHC3* | 9 |  | rs7198076 | *WWOX* | 16 |
| rs6748375 | *PRKCE* | 2 |  | rs2150611 | *SHC3* | 9 |  | rs11648482 | *WWOX* | 16 |
| rs6751395 | *PRKCE* | 2 |  | rs1125467 | *SHC3* | 9 |  | rs12444278 | *WWOX* | 16 |
| rs13024907 | *PRKCE* | 2 |  | rs944482 | *SHC3* | 9 |  | rs10871358 | *WWOX* | 16 |
| rs6751805 | *PRKCE* | 2 |  | rs1331180 | *SHC3* | 9 |  | rs7189021 | *WWOX* | 16 |
| rs12994911 | *PRKCE* | 2 |  | rs2316280 | *SHC3* | 9 |  | rs8052880 | *WWOX* | 16 |
| rs13430530 | *PRKCE* | 2 |  | rs9410448 | *SHC3* | 9 |  | rs16944152 | *WWOX* | 16 |
| rs6742737 | *PRKCE* | 2 |  | rs4877041 | *SHC3* | 9 |  | rs12716865 | *WWOX* | 16 |
| rs3814371 | *PRKCE* | 2 |  | rs6559335 | *SHC3* | 9 |  | rs1110894 | *WWOX* | 16 |
| rs2084428 | *PRKCE* | 2 |  | rs7021743 | *SHC3* | 9 |  | rs4887997 | *WWOX* | 16 |
| rs12467673 | *PRKCE* | 2 |  | rs7048604 | *SHC3* | 9 |  | rs10492908 | *WWOX* | 16 |
| rs6712557 | *PRKCE* | 2 |  | rs944485 | *SHC3* | 9 |  | rs11150124 | *WWOX* | 16 |
| rs11125048 | *PRKCE* | 2 |  | rs9410455 | *SHC3* | 9 |  | rs8060634 | *WWOX* | 16 |
| rs13432276 | *PRKCE* | 2 |  | rs1547696 | *SHC3* | 9 |  | rs12935510 | *WWOX* | 16 |
| rs17034455 | *PRKCE* | 2 |  | rs7864748 | *SHC3* | 9 |  | rs7186745 | *WWOX* | 16 |
| rs1124787 | *PRKCE* | 2 |  | rs4876968 | *SHC3* | 9 |  | rs11150125 | *WWOX* | 16 |
| rs3768761 | *PRKCE* | 2 |  | rs1556384 | *SHC3* | 9 |  | rs4888900 | *WWOX* | 16 |
| rs10167555 | *PRKCE* | 2 |  | rs4534195 | *SHC3* | 9 |  | rs924870 | *WWOX* | 16 |
| rs12712967 | *PRKCE* | 2 |  | rs10867158 | *SHC3* | 9 |  | rs3751834 | *WWOX* | 16 |
| rs3768757 | *PRKCE* | 2 |  | rs1411835 | *SHC3* | 9 |  | rs4620969 | *WWOX* | 16 |
| rs3754570 | *PRKCE* | 2 |  | rs2873094 | *SHC3* | 9 |  | rs16949152 | *WWOX* | 16 |
| rs10865212 | *PRKCE* | 2 |  | rs4879926 | *TLN1* | 9 |  | rs10492907 | *WWOX* | 16 |
| rs1375056 | *PRKCE* | 2 |  | rs867194 | *TLN1* | 9 |  | rs4888910 | *WWOX* | 16 |
| rs1375055 | *PRKCE* | 2 |  | rs2295795 | *TLN1* | 9 |  | rs4888911 | *WWOX* | 16 |
| rs1349080 | *PRKCE* | 2 |  | rs2295797 | *TLN1* | 9 |  | rs17643319 | *WWOX* | 16 |
| rs3768753 | *PRKCE* | 2 |  | rs3763630 | *TLN1* | 9 |  | rs9941213 | *WWOX* | 16 |
| rs12619351 | *PRKCE* | 2 |  | rs3750434 | *TLN1* | 9 |  | rs12929415 | *WWOX* | 16 |
| rs12616328 | *PRKCE* | 2 |  | rs4114992 | *CAMK2G* | 10 |  | rs9932250 | *WWOX* | 16 |
| rs3768751 | *PRKCE* | 2 |  | rs2254266 | *CAMK2G* | 10 |  | rs2006902 | *WWOX* | 16 |
| rs17737466 | *PRKCE* | 2 |  | rs2242258 | *CAMK2G* | 10 |  | rs11644322 | *WWOX* | 16 |
| rs3768748 | *PRKCE* | 2 |  | rs2675671 | *CAMK2G* | 10 |  | rs2113307 | *WWOX* | 16 |
| rs3754569 | *PRKCE* | 2 |  | rs2675677 | *CAMK2G* | 10 |  | rs2550731 | *WWOX* | 16 |
| rs10495927 | *PRKCE* | 2 |  | rs17741873 | *CAMK2G* | 10 |  | rs12447246 | *WWOX* | 16 |
| rs10495928 | *PRKCE* | 2 |  | rs2698761 | *MAPK8* | 10 |  | rs16949222 | *WWOX* | 16 |
| rs4952801 | *PRKCE* | 2 |  | rs10857561 | *MAPK8* | 10 |  | rs16949240 | *WWOX* | 16 |
| rs3768745 | *PRKCE* | 2 |  | rs7075976 | *MAPK8* | 10 |  | rs2656653 | *WWOX* | 16 |
| rs13406773 | *PRKCE* | 2 |  | rs10508902 | *MAPK8* | 10 |  | rs2550717 | *WWOX* | 16 |
| rs13410378 | *PRKCE* | 2 |  | rs1867584 | *MAPK8* | 10 |  | rs8047597 | *WWOX* | 16 |
| rs3754568 | *PRKCE* | 2 |  | rs7907714 | *NRG3* | 10 |  | rs2550710 | *WWOX* | 16 |
| rs10495929 | *PRKCE* | 2 |  | rs2064588 | *NRG3* | 10 |  | rs2550702 | *WWOX* | 16 |
| rs1020445 | *PRKCE* | 2 |  | rs11191705 | *NRG3* | 10 |  | rs16949286 | *WWOX* | 16 |
| rs10205024 | *PRKCE* | 2 |  | rs6584400 | *NRG3* | 10 |  | rs2656646 | *WWOX* | 16 |
| rs3820729 | *PRKCE* | 2 |  | rs7917933 | *NRG3* | 10 |  | rs2656645 | *WWOX* | 16 |
| rs11125055 | *PRKCE* | 2 |  | rs6584407 | *NRG3* | 10 |  | rs8055815 | *WWOX* | 16 |
| rs951012 | *PRKCE* | 2 |  | rs11191796 | *NRG3* | 10 |  | rs2550692 | *WWOX* | 16 |
| rs281476 | *PRKCE* | 2 |  | rs7075126 | *NRG3* | 10 |  | rs1397158 | *WWOX* | 16 |
| rs281472 | *PRKCE* | 2 |  | rs4933812 | *NRG3* | 10 |  | rs905780 | *WWOX* | 16 |
| rs1530668 | *PRKCE* | 2 |  | rs10883934 | *NRG3* | 10 |  | rs2247465 | *WWOX* | 16 |
| rs2594489 | *PRKCE* | 2 |  | rs10786777 | *NRG3* | 10 |  | rs16949366 | *WWOX* | 16 |
| rs281508 | *PRKCE* | 2 |  | rs4933265 | *NRG3* | 10 |  | rs2656612 | *WWOX* | 16 |
| rs14138 | *PRKCE* | 2 |  | rs10883973 | *NRG3* | 10 |  | rs1110898 | *WWOX* | 16 |
| rs281505 | *PRKCE* | 2 |  | rs7902158 | *NRG3* | 10 |  | rs8046508 | *WWOX* | 16 |
| rs281501 | *PRKCE* | 2 |  | rs7914963 | *NRG3* | 10 |  | rs2656609 | *WWOX* | 16 |
| rs281498 | *PRKCE* | 2 |  | rs10509440 | *NRG3* | 10 |  | rs2656667 | *WWOX* | 16 |
| rs281497 | *PRKCE* | 2 |  | rs910583 | *NRG3* | 10 |  | rs2550685 | *WWOX* | 16 |
| rs281489 | *PRKCE* | 2 |  | rs2224111 | *NRG3* | 10 |  | rs2550679 | *WWOX* | 16 |
| rs882586 | *PRKCE* | 2 |  | rs7069222 | *NRG3* | 10 |  | rs9319534 | *WWOX* | 16 |
| rs963731 | *SOS1* | 2 |  | rs6584455 | *NRG3* | 10 |  | rs2347086 | *WWOX* | 16 |
| rs6544186 | *SOS1* | 2 |  | rs4933818 | *NRG3* | 10 |  | rs17647978 | *WWOX* | 16 |
| rs6713281 | *SOS1* | 2 |  | rs2207781 | *NRG3* | 10 |  | rs9930132 | *WWOX* | 16 |
| rs2888586 | *SOS1* | 2 |  | rs3903435 | *NRG3* | 10 |  | rs12446495 | *WWOX* | 16 |
| rs2123882 | *SOS1* | 2 |  | rs9664256 | *NRG3* | 10 |  | rs12600154 | *WWOX* | 16 |
| rs1454227 | *SOS1* | 2 |  | rs7094054 | *NRG3* | 10 |  | rs12926028 | *WWOX* | 16 |
| rs7583010 | *SOS1* | 2 |  | rs7068239 | *NRG3* | 10 |  | rs17648647 | *WWOX* | 16 |
| rs13019754 | *SOS1* | 2 |  | rs10884056 | *NRG3* | 10 |  | rs6564640 | *WWOX* | 16 |
| rs6758330 | *SOS1* | 2 |  | rs10748871 | *NRG3* | 10 |  | rs7199945 | *WWOX* | 16 |
| rs2060989 | *SOS1* | 2 |  | rs10884065 | *NRG3* | 10 |  | rs4888923 | *WWOX* | 16 |
| rs2168043 | *SOS1* | 2 |  | rs10884066 | *NRG3* | 10 |  | rs4888924 | *WWOX* | 16 |
| rs883844 | *STAT1* | 2 |  | rs9804196 | *NRG3* | 10 |  | rs9939718 | *WWOX* | 16 |
| rs12987796 | *STAT1* | 2 |  | rs11192252 | *NRG3* | 10 |  | rs8048899 | *WWOX* | 16 |
| rs3771300 | *STAT1* | 2 |  | rs2207768 | *NRG3* | 10 |  | rs7193279 | *WWOX* | 16 |
| rs6718902 | *STAT1* | 2 |  | rs2881607 | *NRG3* | 10 |  | rs12716868 | *WWOX* | 16 |
| rs13395505 | *STAT1* | 2 |  | rs12767327 | *NRG3* | 10 |  | rs1424104 | *WWOX* | 16 |
| rs9789700 | *STAT1* | 2 |  | rs7907146 | *NRG3* | 10 |  | rs7206890 | *WWOX* | 16 |
| rs2280232 | *STAT1* | 2 |  | rs7091584 | *NRG3* | 10 |  | rs7192197 | *WWOX* | 16 |
| rs11887698 | *STAT1* | 2 |  | rs7081891 | *NRG3* | 10 |  | rs9940536 | *WWOX* | 16 |
| rs7562024 | *STAT1* | 2 |  | rs12415889 | *NRG3* | 10 |  | rs2005036 | *WWOX* | 16 |
| rs12693591 | *STAT1* | 2 |  | rs2347335 | *NRG3* | 10 |  | rs11647295 | *WWOX* | 16 |
| rs16833157 | *STAT1* | 2 |  | rs7074858 | *NRG3* | 10 |  | rs8045079 | *WWOX* | 16 |
| rs13029247 | *STAT1* | 2 |  | rs11192402 | *NRG3* | 10 |  | rs7498200 | *WWOX* | 16 |
| rs10173099 | *STAT1* | 2 |  | rs4562759 | *NRG3* | 10 |  | rs12599773 | *WWOX* | 16 |
| rs13029532 | *STAT1* | 2 |  | rs11192423 | *NRG3* | 10 |  | rs13331974 | *WWOX* | 16 |
| rs6751855 | *STAT1* | 2 |  | rs1884361 | *NRG3* | 10 |  | rs2042437 | *WWOX* | 16 |
| rs6740131 | *STAT1* | 2 |  | rs926929 | *NRG3* | 10 |  | rs1111230 | *WWOX* | 16 |
| rs3024912 | *STAT1* | 2 |  | rs17099378 | *NRG3* | 10 |  | rs1424112 | *WWOX* | 16 |
| rs3024908 | *STAT1* | 2 |  | rs1024192 | *NRG3* | 10 |  | rs7193539 | *WWOX* | 16 |
| rs3024896 | *STAT1* | 2 |  | rs10490935 | *NRG3* | 10 |  | rs7200634 | *WWOX* | 16 |
| rs925847 | *STAT1* | 2 |  | rs7099976 | *NRG3* | 10 |  | rs8050239 | *WWOX* | 16 |
| rs549386 | *TGFA* | 2 |  | rs1649947 | *NRG3* | 10 |  | rs8056647 | *WWOX* | 16 |
| rs488065 | *TGFA* | 2 |  | rs10884225 | *NRG3* | 10 |  | rs9924268 | *WWOX* | 16 |
| rs10496180 | *TGFA* | 2 |  | rs1649934 | *NRG3* | 10 |  | rs4459555 | *WWOX* | 16 |
| rs10172814 | *TGFA* | 2 |  | rs10490933 | *NRG3* | 10 |  | rs1117007 | *WWOX* | 16 |
| rs930655 | *TGFA* | 2 |  | rs1080293 | *NRG3* | 10 |  | rs11864641 | *WWOX* | 16 |
| rs6749533 | *TGFA* | 2 |  | rs1896509 | *NRG3* | 10 |  | rs6564651 | *WWOX* | 16 |
| rs3771515 | *TGFA* | 2 |  | rs1649967 | *NRG3* | 10 |  | rs12443833 | *WWOX* | 16 |
| rs17005644 | *TGFA* | 2 |  | rs10490932 | *NRG3* | 10 |  | rs8051649 | *WWOX* | 16 |
| rs3771512 | *TGFA* | 2 |  | rs11192702 | *NRG3* | 10 |  | rs11150140 | *WWOX* | 16 |
| rs1448927 | *TGFA* | 2 |  | rs10884254 | *NRG3* | 10 |  | rs17656178 | *WWOX* | 16 |
| rs3755384 | *TGFA* | 2 |  | rs1649949 | *NRG3* | 10 |  | rs1116525 | *WWOX* | 16 |
| rs17005666 | *TGFA* | 2 |  | rs10884269 | *NRG3* | 10 |  | rs2011184 | *WWOX* | 16 |
| rs7579830 | *TGFA* | 2 |  | rs12249088 | *NRG3* | 10 |  | rs3751832 | *WWOX* | 16 |
| rs3771504 | *TGFA* | 2 |  | rs1739778 | *NRG3* | 10 |  | rs1834037 | *WWOX* | 16 |
| rs3911077 | *TGFA* | 2 |  | rs602687 | *NRG3* | 10 |  | rs1424106 | *WWOX* | 16 |
| rs3911080 | *TGFA* | 2 |  | rs1739779 | *NRG3* | 10 |  | rs16949819 | *WWOX* | 16 |
| rs3821262 | *TGFA* | 2 |  | rs932512 | *NRG3* | 10 |  | rs1108663 | *WWOX* | 16 |
| rs3771494 | *TGFA* | 2 |  | rs1739765 | *NRG3* | 10 |  | rs377651 | *WWOX* | 16 |
| rs3755377 | *TGFA* | 2 |  | rs1649943 | *NRG3* | 10 |  | rs415738 | *WWOX* | 16 |
| rs404420 | *TGFA* | 2 |  | rs11192921 | *NRG3* | 10 |  | rs414723 | *WWOX* | 16 |
| rs454305 | *TGFA* | 2 |  | rs1336286 | *NRG3* | 10 |  | rs12445467 | *WWOX* | 16 |
| rs375668 | *TGFA* | 2 |  | rs1649940 | *NRG3* | 10 |  | rs383673 | *WWOX* | 16 |
| rs731461 | *TGFA* | 2 |  | rs1336287 | *NRG3* | 10 |  | rs424074 | *WWOX* | 16 |
| rs426081 | *TGFA* | 2 |  | rs12220854 | *NRG3* | 10 |  | rs59344 | *WWOX* | 16 |
| rs374640 | *TGFA* | 2 |  | rs2152596 | *NRG3* | 10 |  | rs454120 | *WWOX* | 16 |
| rs446086 | *TGFA* | 2 |  | rs11192970 | *NRG3* | 10 |  | rs369487 | *WWOX* | 16 |
| rs11466212 | *TGFA* | 2 |  | rs10786961 | *NRG3* | 10 |  | rs2016545 | *WWOX* | 16 |
| rs7562947 | *TGFA* | 2 |  | rs1764072 | *NRG3* | 10 |  | rs17642520 | *WWOX* | 16 |
| rs428225 | *TGFA* | 2 |  | rs1764073 | *NRG3* | 10 |  | rs17796342 | *WWOX* | 16 |
| rs432203 | *TGFA* | 2 |  | rs10884338 | *NRG3* | 10 |  | rs384228 | *WWOX* | 16 |
| rs3771475 | *TGFA* | 2 |  | rs1764074 | *NRG3* | 10 |  | rs403632 | *WWOX* | 16 |
| rs1523300 | *TGFA* | 2 |  | rs12261149 | *NRG3* | 10 |  | rs450829 | *WWOX* | 16 |
| rs10489985 | *TGFA* | 2 |  | rs1414767 | *NRG3* | 10 |  | rs413263 | *WWOX* | 16 |
| rs4852650 | *TGFA* | 2 |  | rs12255719 | *NRG3* | 10 |  | rs12449081 | *WWOX* | 16 |
| rs2215021 | *TGFA* | 2 |  | rs10884342 | *NRG3* | 10 |  | rs383362 | *WWOX* | 16 |
| rs9857831 | *CBLB* | 3 |  | rs2516327 | *NRG3* | 10 |  | rs12828 | *WWOX* | 16 |
| rs11706098 | *CBLB* | 3 |  | rs2992776 | *NRG3* | 10 |  | rs270421 | *WWOX* | 16 |
| rs10490831 | *CBLB* | 3 |  | rs12414020 | *NRG3* | 10 |  | rs270422 | *WWOX* | 16 |
| rs6764799 | *CBLB* | 3 |  | rs17099627 | *NRG3* | 10 |  | rs270425 | *WWOX* | 16 |
| rs1550712 | *CBLB* | 3 |  | rs11193050 | *NRG3* | 10 |  | rs11556635 | *CHRNE* | 17 |
| rs7624530 | *CBLB* | 3 |  | rs10884361 | *NRG3* | 10 |  | rs2302319 | *CHRNE* | 17 |
| rs16851443 | *CBLB* | 3 |  | rs1649962 | *NRG3* | 10 |  | rs3760490 | *CHRNE* | 17 |
| rs3772536 | *CBLB* | 3 |  | rs1336299 | *NRG3* | 10 |  | rs9905341 | *CHRNE* | 17 |
| rs1546733 | *CBLB* | 3 |  | rs7084937 | *NRG3* | 10 |  | rs8081611 | *CHRNE* | 17 |
| rs2301043 | *CBLB* | 3 |  | rs7915399 | *NRG3* | 10 |  | rs877230 | *CRK* | 17 |
| rs2301042 | *CBLB* | 3 |  | rs7085458 | *NRG3* | 10 |  | rs9897348 | *CRK* | 17 |
| rs726443 | *CBLB* | 3 |  | rs11594444 | *NRG3* | 10 |  | rs1083 | *CRK* | 17 |
| rs2305035 | *CBLB* | 3 |  | rs10884421 | *NRG3* | 10 |  | rs10852857 | *CRK* | 17 |
| rs9288817 | *CBLB* | 3 |  | rs12246150 | *NRG3* | 10 |  | rs6416877 | *CRK* | 17 |
| rs13082334 | *CBLB* | 3 |  | rs4147299 | *NRG3* | 10 |  | rs2302456 | *CRK* | 17 |
| rs17280845 | *CBLB* | 3 |  | rs12254772 | *NRG3* | 10 |  | rs314253 | *DLG4* | 17 |
| rs9880861 | *CBLB* | 3 |  | rs7093376 | *NRG3* | 10 |  | rs17203281 | *DLG4* | 17 |
| rs1550711 | *CBLB* | 3 |  | rs4329625 | *NRG3* | 10 |  | rs3826408 | *DLG4* | 17 |
| rs2301028 | *CBLB* | 3 |  | rs11193401 | *NRG3* | 10 |  | rs390200 | *DLG4* | 17 |
| rs16851563 | *CBLB* | 3 |  | rs10509452 | *NRG3* | 10 |  | rs222853 | *DLG4* | 17 |
| rs1947000 | *CBLB* | 3 |  | rs951204 | *NRG3* | 10 |  | rs2074222 | *DLG4* | 17 |
| rs13060223 | *CBLB* | 3 |  | rs11193507 | *NRG3* | 10 |  | rs222837 | *DLG4* | 17 |
| rs6784447 | *CBLB* | 3 |  | rs12251692 | *NRG3* | 10 |  | rs222851 | *DLG4* | 17 |
| rs6780432 | *CBLB* | 3 |  | rs11193663 | *NRG3* | 10 |  | rs222852 | *DLG4* | 17 |
| rs10460808 | *CBLB* | 3 |  | rs4379775 | *NRG3* | 10 |  | rs2941503 | *ERBB2* | 17 |
| rs13062734 | *CBLB* | 3 |  | rs12265675 | *NRG3* | 10 |  | rs1565922 | *ERBB2* | 17 |
| rs769315 | *CBLB* | 3 |  | rs3904725 | *NRG3* | 10 |  | rs12150298 | *ERBB2* | 17 |
| rs2120105 | *CBLB* | 3 |  | rs4143817 | *NRG3* | 10 |  | rs4252627 | *ERBB2* | 17 |
| rs16851630 | *CBLB* | 3 |  | rs7077048 | *NRG3* | 10 |  | rs7219 | *GRB2* | 17 |
| rs7631771 | *CBLB* | 3 |  | rs7087918 | *NRG3* | 10 |  | rs4542691 | *GRB2* | 17 |
| rs1867194 | *CBLB* | 3 |  | rs1317093 | *NRG3* | 10 |  | rs16967789 | *GRB2* | 17 |
| rs13084271 | *CBLB* | 3 |  | rs1937957 | *NRG3* | 10 |  | rs4789172 | *GRB2* | 17 |
| rs6782206 | *CBLB* | 3 |  | rs11193960 | *NRG3* | 10 |  | rs12950752 | *GRB2* | 17 |
| rs7639888 | *CBLB* | 3 |  | rs7075400 | *NRG3* | 10 |  | rs9899687 | *GRB2* | 17 |
| rs2028597 | *CBLB* | 3 |  | rs2022854 | *NRG3* | 10 |  | rs4789182 | *GRB2* | 17 |
| rs6767627 | *CBLB* | 3 |  | rs4933836 | *NRG3* | 10 |  | rs930297 | *GRB2* | 17 |
| rs4894956 | *CBLB* | 3 |  | rs1317008 | *NRG3* | 10 |  | rs7223674 | *GRB2* | 17 |
| rs9841500 | *CBLB* | 3 |  | rs1937984 | *NRG3* | 10 |  | rs9913538 | *GRB2* | 17 |
| rs1910499 | *CBLB* | 3 |  | rs7089201 | *NRG3* | 10 |  | rs9896052 | *GRB2* | 17 |
| rs3957515 | *CBLB* | 3 |  | rs7069578 | *NRG3* | 10 |  | rs8067785 | *MAP2K4* | 17 |
| rs3732360 | *GSK3B* | 3 |  | rs7088585 | *NRG3* | 10 |  | rs7216812 | *MAP2K4* | 17 |
| rs2873950 | *GSK3B* | 3 |  | rs10884697 | *NRG3* | 10 |  | rs17541807 | *MAP2K4* | 17 |
| rs3108749 | *GSK3B* | 3 |  | rs7909484 | *NRG3* | 10 |  | rs12603036 | *MAP2K4* | 17 |
| rs16830594 | *GSK3B* | 3 |  | rs7902584 | *NRG3* | 10 |  | rs2159140 | *MAP2K4* | 17 |
| rs13320980 | *GSK3B* | 3 |  | rs7074029 | *NRG3* | 10 |  | rs12051769 | *MAP2K4* | 17 |
| rs4340737 | *GSK3B* | 3 |  | rs17100087 | *NRG3* | 10 |  | rs11654465 | *MAP2K4* | 17 |
| rs4072520 | *GSK3B* | 3 |  | rs1937968 | *NRG3* | 10 |  | rs9788973 | *MAP2K4* | 17 |
| rs17810235 | *GSK3B* | 3 |  | rs1937969 | *NRG3* | 10 |  | rs8082104 | *MAP2K4* | 17 |
| rs6770314 | *GSK3B* | 3 |  | rs1937970 | *NRG3* | 10 |  | rs8065164 | *MAP2K4* | 17 |
| rs12630592 | *GSK3B* | 3 |  | rs10748994 | *NRG3* | 10 |  | rs1870583 | *MAP2K4* | 17 |
| rs968824 | *GSK3B* | 3 |  | rs4933837 | *NRG3* | 10 |  | rs8078439 | *MAP2K4* | 17 |
| rs2199503 | *GSK3B* | 3 |  | rs10509454 | *NRG3* | 10 |  | rs1870584 | *MAP2K4* | 17 |
| rs11925868 | *GSK3B* | 3 |  | rs670482 | *NRG3* | 10 |  | rs9912036 | *MAP2K4* | 17 |
| rs16830730 | *GSK3B* | 3 |  | rs594612 | *NRG3* | 10 |  | rs7224074 | *MAP2K4* | 17 |
| rs13874 | *LRIG1* | 3 |  | rs11194839 | *NRG3* | 10 |  | rs3744328 | *NCOR1* | 17 |
| rs13071458 | *LRIG1* | 3 |  | rs999889 | *NRG3* | 10 |  | rs8067494 | *NCOR1* | 17 |
| rs7627907 | *LRIG1* | 3 |  | rs664183 | *NRG3* | 10 |  | rs3785628 | *NCOR1* | 17 |
| rs1046844 | *LRIG1* | 3 |  | rs617738 | *NRG3* | 10 |  | rs3760295 | *NCOR1* | 17 |
| rs2242285 | *LRIG1* | 3 |  | rs648958 | *NRG3* | 10 |  | rs2285580 | *NCOR1* | 17 |
| rs9868503 | *LRIG1* | 3 |  | rs11195038 | *NRG3* | 10 |  | rs1989856 | *NCOR1* | 17 |
| rs332370 | *LRIG1* | 3 |  | rs2062020 | *NRG3* | 10 |  | rs7220699 | *NCOR1* | 17 |
| rs2306272 | *LRIG1* | 3 |  | rs11195073 | *NRG3* | 10 |  | rs178831 | *NCOR1* | 17 |
| rs332367 | *LRIG1* | 3 |  | rs12356244 | *NRG3* | 10 |  | rs178810 | *NCOR1* | 17 |
| rs1283532 | *LRIG1* | 3 |  | rs660464 | *NRG3* | 10 |  | rs178798 | *NCOR1* | 17 |
| rs12633819 | *LRIG1* | 3 |  | rs10884989 | *NRG3* | 10 |  | rs178791 | *NCOR1* | 17 |
| rs12494912 | *LRIG1* | 3 |  | rs11195188 | *NRG3* | 10 |  | rs442502 | *PIK3R5* | 17 |
| rs12629792 | *LRIG1* | 3 |  | rs639839 | *NRG3* | 10 |  | rs383123 | *PIK3R5* | 17 |
| rs11925105 | *LRIG1* | 3 |  | rs2820112 | *NRG3* | 10 |  | rs373495 | *PIK3R5* | 17 |
| rs4109626 | *LRIG1* | 3 |  | rs652183 | *NRG3* | 10 |  | rs411268 | *PIK3R5* | 17 |
| rs13073932 | *LRIG1* | 3 |  | rs2247247 | *NRG3* | 10 |  | rs427554 | *PIK3R5* | 17 |
| rs12487264 | *LRIG1* | 3 |  | rs688413 | *NRG3* | 10 |  | rs726679 | *PIK3R5* | 17 |
| rs783511 | *LRIG1* | 3 |  | rs661469 | *NRG3* | 10 |  | rs181536 | *PIK3R5* | 17 |
| rs10049330 | *LRIG1* | 3 |  | rs673049 | *NRG3* | 10 |  | rs9895992 | *PIK3R5* | 17 |
| rs3845895 | *LRIG1* | 3 |  | rs10885058 | *NRG3* | 10 |  | rs12453818 | *PIK3R5* | 17 |
| rs6419763 | *LRIG1* | 3 |  | rs12415032 | *NRG3* | 10 |  | rs4791769 | *PIK3R5* | 17 |
| rs9849546 | *LRIG1* | 3 |  | rs1832586 | *NRG3* | 10 |  | rs11655080 | *PRKCA* | 17 |
| rs3796150 | *LRIG1* | 3 |  | rs10749058 | *NRG3* | 10 |  | rs11658474 | *PRKCA* | 17 |
| rs11706832 | *LRIG1* | 3 |  | rs2255228 | *NRG3* | 10 |  | rs8081614 | *PRKCA* | 17 |
| rs13325263 | *LRIG1* | 3 |  | rs1339797 | *NRG3* | 10 |  | rs9897002 | *PRKCA* | 17 |
| rs3845899 | *LRIG1* | 3 |  | rs17100444 | *NRG3* | 10 |  | rs12948922 | *PRKCA* | 17 |
| rs6549120 | *LRIG1* | 3 |  | rs4532983 | *NRG3* | 10 |  | rs11867898 | *PRKCA* | 17 |
| rs10510956 | *LRIG1* | 3 |  | rs12416296 | *NRG3* | 10 |  | rs12150089 | *PRKCA* | 17 |
| rs9846493 | *LRIG1* | 3 |  | rs4586096 | *NRG3* | 10 |  | rs9892651 | *PRKCA* | 17 |
| rs3845906 | *LRIG1* | 3 |  | rs3924461 | *NRG3* | 10 |  | rs16959046 | *PRKCA* | 17 |
| rs12637858 | *LRIG1* | 3 |  | rs2494026 | *NRG3* | 10 |  | rs9901804 | *PRKCA* | 17 |
| rs6782090 | *LRIG1* | 3 |  | rs2246633 | *NRG3* | 10 |  | rs4433842 | *PRKCA* | 17 |
| rs11709533 | *LRIG1* | 3 |  | rs7068082 | *NRG3* | 10 |  | rs12450534 | *PRKCA* | 17 |
| rs7646811 | *LRIG1* | 3 |  | rs1572334 | *NRG3* | 10 |  | rs8078231 | *PRKCA* | 17 |
| rs6789596 | *LRIG1* | 3 |  | rs1572333 | *NRG3* | 10 |  | rs11654719 | *PRKCA* | 17 |
| rs11707571 | *LRIG1* | 3 |  | rs1889706 | *NRG3* | 10 |  | rs9914703 | *PRKCA* | 17 |
| rs1052618 | *NCK1* | 3 |  | rs1889709 | *NRG3* | 10 |  | rs4411531 | *PRKCA* | 17 |
| rs7648198 | *NCK1* | 3 |  | rs915349 | *NRG3* | 10 |  | rs9892886 | *PRKCA* | 17 |
| rs835636 | *NCK1* | 3 |  | rs2249075 | *NRG3* | 10 |  | rs4536508 | *PRKCA* | 17 |
| rs835634 | *NCK1* | 3 |  | rs2050458 | *NRG3* | 10 |  | rs17634425 | *PRKCA* | 17 |
| rs2049233 | *PAK2* | 3 |  | rs10509459 | *NRG3* | 10 |  | rs6504424 | *PRKCA* | 17 |
| rs1463628 | *PAK2* | 3 |  | rs2820096 | *NRG3* | 10 |  | rs8080771 | *PRKCA* | 17 |
| rs1403101 | *PAK2* | 3 |  | rs2820100 | *NRG3* | 10 |  | rs4290512 | *PRKCA* | 17 |
| rs9872035 | *PAK2* | 3 |  | rs2644206 | *NRG3* | 10 |  | rs4561502 | *PRKCA* | 17 |
| rs2089979 | *PAK2* | 3 |  | rs17682831 | *NRG3* | 10 |  | rs4417581 | *PRKCA* | 17 |
| rs9325377 | *PAK2* | 3 |  | rs7903899 | *NRG3* | 10 |  | rs16959216 | *PRKCA* | 17 |
| rs7646247 | *PAK2* | 3 |  | rs571797 | *NRG3* | 10 |  | rs11652956 | *PRKCA* | 17 |
| rs6583176 | *PAK2* | 3 |  | rs514237 | *NRG3* | 10 |  | rs4790911 | *PRKCA* | 17 |
| rs10446497 | *PAK2* | 3 |  | rs342393 | *NRG3* | 10 |  | rs7221968 | *PRKCA* | 17 |
| rs6583177 | *PAK2* | 3 |  | rs2644212 | *NRG3* | 10 |  | rs16959238 | *PRKCA* | 17 |
| rs6807293 | *PIK3CA* | 3 |  | rs1495862 | *NRG3* | 10 |  | rs9913908 | *PRKCA* | 17 |
| rs2699905 | *PIK3CA* | 3 |  | rs12783350 | *NRG3* | 10 |  | rs973753 | *PRKCA* | 17 |
| rs7641889 | *PIK3CA* | 3 |  | rs11195879 | *NRG3* | 10 |  | rs7405806 | *PRKCA* | 17 |
| rs6443624 | *PIK3CA* | 3 |  | rs7477796 | *NRG3* | 10 |  | rs11079657 | *PRKCA* | 17 |
| rs7646409 | *PIK3CA* | 3 |  | rs1333211 | *NRG3* | 10 |  | rs228883 | *PRKCA* | 17 |
| rs2677760 | *PIK3CA* | 3 |  | rs11195974 | *NRG3* | 10 |  | rs7207499 | *PRKCA* | 17 |
| rs13082485 | *PIK3CA* | 3 |  | rs7919853 | *NRG3* | 10 |  | rs3848426 | *PRKCA* | 17 |
| rs7614305 | *PIK3CA* | 3 |  | rs1333214 | *NRG3* | 10 |  | rs8074294 | *PRKCA* | 17 |
| rs1607237 | *PIK3CA* | 3 |  | rs2483307 | *NRG3* | 10 |  | rs4622543 | *PRKCA* | 17 |
| rs9878820 | *PIK3CB* | 3 |  | rs342368 | *NRG3* | 10 |  | rs956952 | *PRKCA* | 17 |
| rs531577 | *PIK3CB* | 3 |  | rs715687 | *NRG3* | 10 |  | rs9896483 | *PRKCA* | 17 |
| rs361068 | *PIK3CB* | 3 |  | rs11196077 | *NRG3* | 10 |  | rs2138004 | *PRKCA* | 17 |
| rs7615501 | *PRKCD* | 3 |  | rs1333217 | *NRG3* | 10 |  | rs1806448 | *PRKCA* | 17 |
| rs6807359 | *PRKCD* | 3 |  | rs193178 | *NRG3* | 10 |  | rs6504441 | *PRKCA* | 17 |
| rs6777221 | *PRKCD* | 3 |  | rs7069209 | *NRG3* | 10 |  | rs10512512 | *PRKCA* | 17 |
| rs13085294 | *PRKCD* | 3 |  | rs342375 | *NRG3* | 10 |  | rs9303510 | *PRKCA* | 17 |
| rs1483185 | *PRKCD* | 3 |  | rs490164 | *NRG3* | 10 |  | rs16959569 | *PRKCA* | 17 |
| rs3773732 | *PRKCD* | 3 |  | rs2348553 | *NRG3* | 10 |  | rs11656279 | *PRKCA* | 17 |
| rs3773729 | *PRKCD* | 3 |  | rs342379 | *NRG3* | 10 |  | rs7220127 | *PRKCA* | 17 |
| rs6778964 | *PRKCD* | 3 |  | rs12415782 | *NRG3* | 10 |  | rs741141 | *PRKCA* | 17 |
| rs17052826 | *PRKCD* | 3 |  | rs342386 | *NRG3* | 10 |  | rs1003599 | *PRKCA* | 17 |
| rs2306572 | *PRKCD* | 3 |  | rs560083 | *NRG3* | 10 |  | rs1860984 | *PRKCA* | 17 |
| rs729396 | *PRKCD* | 3 |  | rs498979 | *NRG3* | 10 |  | rs11871468 | *PRKCA* | 17 |
| rs12490645 | *PRKCD* | 3 |  | rs17100953 | *NRG3* | 10 |  | rs12452826 | *PRKCA* | 17 |
| rs11919522 | *PRKCD* | 3 |  | rs512064 | *NRG3* | 10 |  | rs12938937 | *PRKCA* | 17 |
| rs713178 | *RAF1* | 3 |  | rs540697 | *NRG3* | 10 |  | rs9904266 | *PRKCA* | 17 |
| rs9855183 | *RAF1* | 3 |  | rs11196301 | *NRG3* | 10 |  | rs9905351 | *PRKCA* | 17 |
| rs15997 | *RAF1* | 3 |  | rs2132377 | *NRG3* | 10 |  | rs11869197 | *PRKCA* | 17 |
| rs5746255 | *RAF1* | 3 |  | rs2475793 | *NRG3* | 10 |  | rs8069696 | *PRKCA* | 17 |
| rs7956 | *RAF1* | 3 |  | rs580333 | *NRG3* | 10 |  | rs887797 | *PRKCA* | 17 |
| rs1532533 | *RAF1* | 3 |  | rs544711 | *NRG3* | 10 |  | rs9646423 | *PRKCA* | 17 |
| rs1532534 | *RAF1* | 3 |  | rs489466 | *NRG3* | 10 |  | rs8080721 | *PRKCA* | 17 |
| rs9809501 | *RAF1* | 3 |  | rs4326733 | *NRG3* | 10 |  | rs9896134 | *PRKCA* | 17 |
| rs4234512 | *RAF1* | 3 |  | rs1412919 | *NRG3* | 10 |  | rs17759410 | *PRKCA* | 17 |
| rs7643321 | *RAF1* | 3 |  | rs1333208 | *NRG3* | 10 |  | rs9898223 | *PRKCA* | 17 |
| rs6792773 | *RAF1* | 3 |  | rs7079963 | *NRG3* | 10 |  | rs9908814 | *PRKCA* | 17 |
| rs11128607 | *RAF1* | 3 |  | rs11819224 | *NRG3* | 10 |  | rs17686425 | *PRKCA* | 17 |
| rs9817675 | *RAF1* | 3 |  | rs580293 | *NRG3* | 10 |  | rs16959714 | *PRKCA* | 17 |
| rs6442322 | *RAF1* | 3 |  | rs580298 | *NRG3* | 10 |  | rs759115 | *PRKCA* | 17 |
| rs904453 | *RAF1* | 3 |  | rs492203 | *NRG3* | 10 |  | rs2024321 | *PRKCA* | 17 |
| rs7617041 | *RAF1* | 3 |  | rs1576987 | *NRG3* | 10 |  | rs10491204 | *PRKCA* | 17 |
| rs6442323 | *RAF1* | 3 |  | rs12416404 | *NRG3* | 10 |  | rs877447 | *PRKCA* | 17 |
| rs1691274 | *AREG* | 4 |  | rs1475706 | *NRG3* | 10 |  | rs1006841 | *PRKCA* | 17 |
| rs11725706 | *AREG* | 4 |  | rs2026495 | *NRG3* | 10 |  | rs9907959 | *PRKCA* | 17 |
| rs12503079 | *BTC* | 4 |  | rs12771439 | *NRG3* | 10 |  | rs9889698 | *PRKCA* | 17 |
| rs4345250 | *BTC* | 4 |  | rs2348754 | *NRG3* | 10 |  | rs1860985 | *PRKCA* | 17 |
| rs7667014 | *BTC* | 4 |  | rs17101139 | *NRG3* | 10 |  | rs2362707 | *PRKCA* | 17 |
| rs4859417 | *BTC* | 4 |  | rs4362091 | *NRG3* | 10 |  | rs7207345 | *PRKCA* | 17 |
| rs4352548 | *BTC* | 4 |  | rs7092994 | *NRG3* | 10 |  | rs957944 | *PRKCA* | 17 |
| rs6843005 | *BTC* | 4 |  | rs3818306 | *NRG3* | 10 |  | rs957946 | *PRKCA* | 17 |
| rs6532431 | *BTC* | 4 |  | rs1923547 | *NRG3* | 10 |  | rs17688881 | *PRKCA* | 17 |
| rs4076318 | *BTC* | 4 |  | rs12644 | *PPP3CB* | 10 |  | rs16959970 | *PRKCA* | 17 |
| rs10518128 | *BTC* | 4 |  | rs3763679 | *PPP3CB* | 10 |  | rs16960009 | *PRKCA* | 17 |
| rs1349634 | *BTC* | 4 |  | rs3793663 | *PPP3CB* | 10 |  | rs1010546 | *PRKCA* | 17 |
| rs1349632 | *BTC* | 4 |  | rs10887758 | *PTEN* | 10 |  | rs1010544 | *PRKCA* | 17 |
| rs16999874 | *BTC* | 4 |  | rs1234212 | *PTEN* | 10 |  | rs16960050 | *PRKCA* | 17 |
| rs12643280 | *BTC* | 4 |  | rs1234221 | *PTEN* | 10 |  | rs16960070 | *PRKCA* | 17 |
| rs1377028 | *BTC* | 4 |  | rs1234220 | *PTEN* | 10 |  | rs7217618 | *PRKCA* | 17 |
| rs3733619 | *CAMK2D* | 4 |  | rs2299939 | *PTEN* | 10 |  | rs10491200 | *PRKCA* | 17 |
| rs17046072 | *CAMK2D* | 4 |  | rs2248293 | *PTEN* | 10 |  | rs9894916 | *PRKCA* | 17 |
| rs1525000 | *CAMK2D* | 4 |  | rs11202607 | *PTEN* | 10 |  | rs8081512 | *PRKCA* | 17 |
| rs2285703 | *CAMK2D* | 4 |  | rs478839 | *PTEN* | 10 |  | rs8069926 | *PRKCA* | 17 |
| rs6835747 | *CAMK2D* | 4 |  | rs10509532 | *PTEN* | 10 |  | rs16960110 | *PRKCA* | 17 |
| rs916874 | *CAMK2D* | 4 |  | rs915987 | *BAD* | 11 |  | rs16960114 | *PRKCA* | 17 |
| rs6842886 | *CAMK2D* | 4 |  | rs660442 | *BAD* | 11 |  | rs7219495 | *PRKCA* | 17 |
| rs4834340 | *CAMK2D* | 4 |  | rs477895 | *BAD* | 11 |  | rs3889392 | *PRKCA* | 17 |
| rs4833430 | *CAMK2D* | 4 |  | rs2286614 | *BAD* | 11 |  | rs11079667 | *PRKCA* | 17 |
| rs2158196 | *CAMK2D* | 4 |  | rs1815811 | *CBL* | 11 |  | rs8067877 | *PRKCA* | 17 |
| rs4834348 | *CAMK2D* | 4 |  | rs12417928 | *CBL* | 11 |  | rs6504452 | *PRKCA* | 17 |
| rs11938486 | *CAMK2D* | 4 |  | rs11217191 | *CBL* | 11 |  | rs7215445 | *PRKCA* | 17 |
| rs4240286 | *CAMK2D* | 4 |  | rs7946919 | *CBL* | 11 |  | rs6504453 | *PRKCA* | 17 |
| rs757175 | *CAMK2D* | 4 |  | rs2510152 | *CBL* | 11 |  | rs4303580 | *PRKCA* | 17 |
| rs2285704 | *CAMK2D* | 4 |  | rs2510139 | *CBL* | 11 |  | rs7224633 | *PRKCA* | 17 |
| rs2051785 | *CAMK2D* | 4 |  | rs1047417 | *CBL* | 11 |  | rs4644888 | *PRKCA* | 17 |
| rs17630328 | *CAMK2D* | 4 |  | rs7914 | *CBL* | 11 |  | rs9893560 | *PRKCA* | 17 |
| rs10009286 | *CAMK2D* | 4 |  | rs2249466 | *CBL* | 11 |  | rs12232511 | *PRKCA* | 17 |
| rs6533700 | *CAMK2D* | 4 |  | rs4963136 | *HRAS* | 11 |  | rs4381631 | *PRKCA* | 17 |
| rs7665102 | *CAMK2D* | 4 |  | rs7396875 | *HRAS* | 11 |  | rs4790904 | *PRKCA* | 17 |
| rs17531701 | *CAMK2D* | 4 |  | rs4944158 | *PAK1* | 11 |  | rs3889237 | *PRKCA* | 17 |
| rs7681248 | *CAMK2D* | 4 |  | rs1377470 | *PAK1* | 11 |  | rs6504458 | *PRKCA* | 17 |
| rs10025791 | *CAMK2D* | 4 |  | rs2602474 | *PAK1* | 11 |  | rs9890506 | *PRKCA* | 17 |
| rs10033037 | *CAMK2D* | 4 |  | rs2729760 | *PAK1* | 11 |  | rs16960228 | *PRKCA* | 17 |
| rs12500935 | *CAMK2D* | 4 |  | rs2729747 | *PAK1* | 11 |  | rs8074995 | *PRKCA* | 17 |
| rs1047187 | *CAMK2D* | 4 |  | rs2729749 | *PAK1* | 11 |  | rs4791036 | *PRKCA* | 17 |
| rs916913 | *CAMK2D* | 4 |  | rs495889 | *PAK1* | 11 |  | rs7342847 | *PRKCA* | 17 |
| rs7681664 | *CAMK2D* | 4 |  | rs2725830 | *PAK1* | 11 |  | rs8464 | *PRKCA* | 17 |
| rs2189368 | *CAMK2D* | 4 |  | rs2852394 | *PAK1* | 11 |  | rs12603061 | *PRKCA* | 17 |
| rs17630766 | *CAMK2D* | 4 |  | rs3019251 | *PAK1* | 11 |  | rs4791032 | *PRKCA* | 17 |
| rs10488959 | *CAMK2D* | 4 |  | rs3019238 | *PAK1* | 11 |  | rs1292053 | *RPS6KB1* | 17 |
| rs12512712 | *CAMK2D* | 4 |  | rs1790733 | *RPS6KB2* | 11 |  | rs8071475 | *RPS6KB1* | 17 |
| rs11935698 | *CAMK2D* | 4 |  | rs1626067 | *RPS6KB2* | 11 |  | rs1292035 | *RPS6KB1* | 17 |
| rs6845568 | *CAMK2D* | 4 |  | rs1638588 | *RPS6KB2* | 11 |  | rs1292034 | *RPS6KB1* | 17 |
| rs9307395 | *CAMK2D* | 4 |  | rs2302264 | *RPS6KB2* | 11 |  | rs180535 | *RPS6KB1* | 17 |
| rs13107662 | *CAMK2D* | 4 |  | rs10895252 | *YAP1* | 11 |  | rs7217337 | *RPS6KB1* | 17 |
| rs2107172 | *CAMK2D* | 4 |  | rs10750630 | *YAP1* | 11 |  | rs180515 | *RPS6KB1* | 17 |
| rs6533710 | *CAMK2D* | 4 |  | rs1426398 | *YAP1* | 11 |  | rs12601982 | *STAT3* | 17 |
| rs1011973 | *CAMK2D* | 4 |  | rs1942691 | *YAP1* | 11 |  | rs8074524 | *STAT3* | 17 |
| rs1029471 | *CAMK2D* | 4 |  | rs12420608 | *YAP1* | 11 |  | rs8069645 | *STAT3* | 17 |
| rs7670019 | *CAMK2D* | 4 |  | rs7928547 | *YAP1* | 11 |  | rs744166 | *STAT3* | 17 |
| rs7660775 | *CAMK2D* | 4 |  | rs11225148 | *YAP1* | 11 |  | rs17405722 | *STAT3* | 17 |
| rs764830 | *CAMK2D* | 4 |  | rs7108698 | *YAP1* | 11 |  | rs4796649 | *STAT3* | 17 |
| rs987694 | *CAMK2D* | 4 |  | rs1426394 | *YAP1* | 11 |  | rs2883456 | *STAT3* | 17 |
| rs2040742 | *CAMK2D* | 4 |  | rs1942683 | *YAP1* | 11 |  | rs9252 | *STAT3* | 17 |
| rs9993234 | *CAMK2D* | 4 |  | rs17097475 | *YAP1* | 11 |  | rs8064638 | *STAT5A* | 17 |
| rs11098200 | *CAMK2D* | 4 |  | rs7106107 | *YAP1* | 11 |  | rs12601982 | *STAT5A* | 17 |
| rs1859229 | *CAMK2D* | 4 |  | rs11225161 | *YAP1* | 11 |  | rs8074524 | *STAT5A* | 17 |
| rs10488961 | *CAMK2D* | 4 |  | rs1894116 | *YAP1* | 11 |  | rs9900213 | *STAT5B* | 17 |
| rs3822295 | *CAMK2D* | 4 |  | rs7107909 | *YAP1* | 11 |  | rs6503691 | *STAT5B* | 17 |
| rs10018022 | *CAMK2D* | 4 |  | rs7110355 | *YAP1* | 11 |  | rs8082391 | *STAT5B* | 17 |
| rs11935493 | *CAMK2D* | 4 |  | rs11225170 | *YAP1* | 11 |  | rs8064638 | *STAT5B* | 17 |
| rs1557815 | *CAMK2D* | 4 |  | rs2282652 | *YAP1* | 11 |  | rs4802070 | *AKT2* | 19 |
| rs1476569 | *CAMK2D* | 4 |  | rs11055024 | *CDKN1B* | 12 |  | rs2304185 | *AKT2* | 19 |
| rs718768 | *EGF* | 4 |  | rs10845613 | *CDKN1B* | 12 |  | rs874269 | *AKT2* | 19 |
| rs17238095 | *EGF* | 4 |  | rs2417223 | *CDKN1B* | 12 |  | rs892119 | *AKT2* | 19 |
| rs2250724 | *EGF* | 4 |  | rs3759216 | *CDKN1B* | 12 |  | rs3730050 | *AKT2* | 19 |
| rs4698800 | *EGF* | 4 |  | rs3759217 | *CDKN1B* | 12 |  | rs2965121 | *CBLC* | 19 |
| rs3796947 | *EGF* | 4 |  | rs34330 | *CDKN1B* | 12 |  | rs10419669 | *CBLC* | 19 |
| rs3796944 | *EGF* | 4 |  | rs2066827 | *CDKN1B* | 12 |  | rs350818 | *MAP2K2* | 19 |
| rs1024599 | *EGF* | 4 |  | rs34322 | *CDKN1B* | 12 |  | rs350886 | *MAP2K2* | 19 |
| rs2067004 | *EGF* | 4 |  | rs10845619 | *CDKN1B* | 12 |  | rs350916 | *MAP2K2* | 19 |
| rs9992755 | *EGF* | 4 |  | rs705708 | *ERBB3* | 12 |  | rs350912 | *MAP2K2* | 19 |
| rs10011112 | *EGF* | 4 |  | rs10783779 | *ERBB3* | 12 |  | rs350897 | *MAP2K2* | 19 |
| rs11568975 | *EGF* | 4 |  | rs2160517 | *GRIN2B* | 12 |  | rs350896 | *MAP2K2* | 19 |
| rs2255355 | *EGF* | 4 |  | rs2193149 | *GRIN2B* | 12 |  | rs4525614 | *MAP2K2* | 19 |
| rs9991367 | *EGF* | 4 |  | rs966664 | *GRIN2B* | 12 |  | rs12459940 | *MAP2K2* | 19 |
| rs11568994 | *EGF* | 4 |  | rs1805494 | *GRIN2B* | 12 |  | rs350887 | *MAP2K2* | 19 |
| rs2237051 | *EGF* | 4 |  | rs3026173 | *GRIN2B* | 12 |  | rs164632 | *MAP2K2* | 19 |
| rs6825106 | *EGF* | 4 |  | rs1805502 | *GRIN2B* | 12 |  | rs350825 | *MAP2K2* | 19 |
| rs2298999 | *EGF* | 4 |  | rs1806201 | *GRIN2B* | 12 |  | rs350824 | *MAP2K2* | 19 |
| rs2299001 | *EGF* | 4 |  | rs1806202 | *GRIN2B* | 12 |  | rs483808 | *MAP2K7* | 19 |
| rs3822286 | *EGF* | 4 |  | rs4764011 | *GRIN2B* | 12 |  | rs3745383 | *MAP2K7* | 19 |
| rs3733625 | *EGF* | 4 |  | rs2270359 | *GRIN2B* | 12 |  | rs4804833 | *MAP2K7* | 19 |
| rs4698804 | *EGF* | 4 |  | rs2072539 | *GRIN2B* | 12 |  | rs3745386 | *MAP2K7* | 19 |
| rs10938121 | *EREG* | 4 |  | rs1806194 | *GRIN2B* | 12 |  | rs1672661 | *PAK4* | 19 |
| rs1812149 | *EREG* | 4 |  | rs1806213 | *GRIN2B* | 12 |  | rs692364 | *PAK4* | 19 |
| rs7673295 | *EREG* | 4 |  | rs11055528 | *GRIN2B* | 12 |  | rs8106725 | *PAK4* | 19 |
| rs7674887 | *EREG* | 4 |  | rs10772693 | *GRIN2B* | 12 |  | rs12609418 | *PAK4* | 19 |
| rs1350666 | *EREG* | 4 |  | rs17220306 | *GRIN2B* | 12 |  | rs692191 | *PAK4* | 19 |
| rs968061 | *EREG* | 4 |  | rs2193147 | *GRIN2B* | 12 |  | rs11669124 | *PAK4* | 19 |
| rs3806794 | *EREG* | 4 |  | rs3026168 | *GRIN2B* | 12 |  | rs17722300 | *PAK4* | 19 |
| rs1460009 | *EREG* | 4 |  | rs4763354 | *GRIN2B* | 12 |  | rs691577 | *PAK4* | 19 |
| rs1460013 | *EREG* | 4 |  | rs11055530 | *GRIN2B* | 12 |  | rs7257109 | *PAK4* | 19 |
| rs6816797 | *EREG* | 4 |  | rs7970177 | *GRIN2B* | 12 |  | rs1529712 | *PAK4* | 19 |
| rs10002688 | *EREG* | 4 |  | rs1805490 | *GRIN2B* | 12 |  | rs9676717 | *PAK4* | 19 |
| rs300905 | *GAB1* | 4 |  | rs1805474 | *GRIN2B* | 12 |  | rs885683 | *PIK3R2* | 19 |
| rs300890 | *GAB1* | 4 |  | rs888150 | *GRIN2B* | 12 |  | rs3746188 | *PIK3R2* | 19 |
| rs13146448 | *GAB1* | 4 |  | rs1805510 | *GRIN2B* | 12 |  | rs8108738 | *PIK3R2* | 19 |
| rs300916 | *GAB1* | 4 |  | rs2268097 | *GRIN2B* | 12 |  | rs1566028 | *PIK3R2* | 19 |
| rs300913 | *GAB1* | 4 |  | rs10845809 | *GRIN2B* | 12 |  | rs1011320 | *PIK3R2* | 19 |
| rs2055730 | *GAB1* | 4 |  | rs1805543 | *GRIN2B* | 12 |  | rs273266 | *PIK3R2* | 19 |
| rs3805251 | *GAB1* | 4 |  | rs2160519 | *GRIN2B* | 12 |  | rs7125 | *PIK3R2* | 19 |
| rs1397528 | *GAB1* | 4 |  | rs1805545 | *GRIN2B* | 12 |  | rs4807492 | *PIP5K1C* | 19 |
| rs10519691 | *GAB1* | 4 |  | rs12229800 | *GRIN2B* | 12 |  | rs2271875 | *PIP5K1C* | 19 |
| rs4690785 | *GAB1* | 4 |  | rs2300238 | *GRIN2B* | 12 |  | rs4807493 | *PIP5K1C* | 19 |
| rs3805236 | *GAB1* | 4 |  | rs980365 | *GRIN2B* | 12 |  | rs3746124 | *PIP5K1C* | 19 |
| rs13128753 | *GAB1* | 4 |  | rs2268102 | *GRIN2B* | 12 |  | rs2270083 | *PIP5K1C* | 19 |
| rs7696619 | *GAB1* | 4 |  | rs2284406 | *GRIN2B* | 12 |  | rs757454 | *PIP5K1C* | 19 |
| rs1397529 | *GAB1* | 4 |  | rs1008619 | *GRIN2B* | 12 |  | rs8109485 | *PIP5K1C* | 19 |
| rs11947756 | *GAB1* | 4 |  | rs2268105 | *GRIN2B* | 12 |  | rs11672559 | *PIP5K1C* | 19 |
| rs2575676 | *MAPK10* | 4 |  | rs2193151 | *GRIN2B* | 12 |  | rs740873 | *PIP5K1C* | 19 |
| rs7438872 | *MAPK10* | 4 |  | rs10492132 | *GRIN2B* | 12 |  | rs8100676 | *PRKACA* | 19 |
| rs4640664 | *MAPK10* | 4 |  | rs11831620 | *GRIN2B* | 12 |  | rs3786661 | *PRKACA* | 19 |
| rs17011297 | *MAPK10* | 4 |  | rs4764026 | *GRIN2B* | 12 |  | rs10421422 | *PRKACA* | 19 |
| rs2589523 | *MAPK10* | 4 |  | rs2268109 | *GRIN2B* | 12 |  | rs307941 | *PRKCG* | 19 |
| rs2575675 | *MAPK10* | 4 |  | rs2300244 | *GRIN2B* | 12 |  | rs454006 | *PRKCG* | 19 |
| rs7699978 | *MAPK10* | 4 |  | rs2284408 | *GRIN2B* | 12 |  | rs3745405 | *PRKCG* | 19 |
| rs2589506 | *MAPK10* | 4 |  | rs918065 | *GRIN2B* | 12 |  | rs753719 | *SHC2* | 19 |
| rs3775167 | *MAPK10* | 4 |  | rs11055584 | *GRIN2B* | 12 |  | rs15971 | *SHC2* | 19 |
| rs2043649 | *MAPK10* | 4 |  | rs11055586 | *GRIN2B* | 12 |  | rs1046822 | *SHC2* | 19 |
| rs2282598 | *MAPK10* | 4 |  | rs11055587 | *GRIN2B* | 12 |  | rs10426188 | *SHC2* | 19 |
| rs3775173 | *MAPK10* | 4 |  | rs1158541 | *GRIN2B* | 12 |  | rs10408164 | *SHC2* | 19 |
| rs1436522 | *MAPK10* | 4 |  | rs2192975 | *GRIN2B* | 12 |  | rs8112380 | *SHC2* | 19 |
| rs6815306 | *MAPK10* | 4 |  | rs2268110 | *GRIN2B* | 12 |  | rs10413199 | *SHC2* | 19 |
| rs3775180 | *MAPK10* | 4 |  | rs7297761 | *GRIN2B* | 12 |  | rs4919871 | *SHC2* | 19 |
| rs3775182 | *MAPK10* | 4 |  | rs7979850 | *GRIN2B* | 12 |  | rs740871 | *SHC2* | 19 |
| rs3775183 | *MAPK10* | 4 |  | rs1012586 | *GRIN2B* | 12 |  | rs4919890 | *SHC2* | 19 |
| rs1469869 | *MAPK10* | 4 |  | rs11836523 | *GRIN2B* | 12 |  | rs2392794 | *SHC2* | 19 |
| rs9995879 | *MAPK10* | 4 |  | rs12809496 | *GRIN2B* | 12 |  | rs10485505 | *ITCH* | 20 |
| rs1898248 | *MAPK10* | 4 |  | rs7968071 | *GRIN2B* | 12 |  | rs6120650 | *ITCH* | 20 |
| rs12644947 | *MAPK10* | 4 |  | rs10845827 | *GRIN2B* | 12 |  | rs2072952 | *PAK7* | 20 |
| rs12640395 | *MAPK10* | 4 |  | rs17220663 | *GRIN2B* | 12 |  | rs2206386 | *PAK7* | 20 |
| rs11943376 | *MAPK10* | 4 |  | rs2300250 | *GRIN2B* | 12 |  | rs6039502 | *PAK7* | 20 |
| rs5006575 | *MAPK10* | 4 |  | rs2300252 | *GRIN2B* | 12 |  | rs10485733 | *PAK7* | 20 |
| rs4403040 | *MAPK10* | 4 |  | rs2284411 | *GRIN2B* | 12 |  | rs6133723 | *PAK7* | 20 |
| rs12647506 | *MAPK10* | 4 |  | rs2300257 | *GRIN2B* | 12 |  | rs2026339 | *PAK7* | 20 |
| rs4352468 | *MAPK10* | 4 |  | rs2268118 | *GRIN2B* | 12 |  | rs9967942 | *PAK7* | 20 |
| rs6854208 | *MAPK10* | 4 |  | rs11055603 | *GRIN2B* | 12 |  | rs6039512 | *PAK7* | 20 |
| rs6822478 | *MAPK10* | 4 |  | rs2110984 | *GRIN2B* | 12 |  | rs6140971 | *PAK7* | 20 |
| rs1460761 | *MAPK10* | 4 |  | rs11612284 | *GRIN2B* | 12 |  | rs6056732 | *PAK7* | 20 |
| rs2199309 | *MAPK10* | 4 |  | rs2268120 | *GRIN2B* | 12 |  | rs6056733 | *PAK7* | 20 |
| rs7668374 | *MAPK10* | 4 |  | rs2216128 | *GRIN2B* | 12 |  | rs6056734 | *PAK7* | 20 |
| rs1599313 | *MAPK10* | 4 |  | rs2192973 | *GRIN2B* | 12 |  | rs6056740 | *PAK7* | 20 |
| rs12650052 | *MAPK10* | 4 |  | rs2268122 | *GRIN2B* | 12 |  | rs6140981 | *PAK7* | 20 |
| rs1460769 | *MAPK10* | 4 |  | rs7307315 | *GRIN2B* | 12 |  | rs928366 | *PAK7* | 20 |
| rs11725943 | *MAPK10* | 4 |  | rs11055608 | *GRIN2B* | 12 |  | rs2225471 | *PAK7* | 20 |
| rs9307016 | *MAPK10* | 4 |  | rs11055612 | *GRIN2B* | 12 |  | rs6056750 | *PAK7* | 20 |
| rs1460756 | *MAPK10* | 4 |  | rs10845840 | *GRIN2B* | 12 |  | rs6133731 | *PAK7* | 20 |
| rs6844363 | *MAPK10* | 4 |  | rs220549 | *GRIN2B* | 12 |  | rs6056755 | *PAK7* | 20 |
| rs17011645 | *MAPK10* | 4 |  | rs11055616 | *GRIN2B* | 12 |  | rs6039524 | *PAK7* | 20 |
| rs6855169 | *MAPK10* | 4 |  | rs7301500 | *GRIN2B* | 12 |  | rs219855 | *PAK7* | 20 |
| rs12640392 | *MAPK10* | 4 |  | rs7301754 | *GRIN2B* | 12 |  | rs219861 | *PAK7* | 20 |
| rs7655217 | *MAPK10* | 4 |  | rs2284418 | *GRIN2B* | 12 |  | rs219862 | *PAK7* | 20 |
| rs4693763 | *MAPK10* | 4 |  | rs7974275 | *GRIN2B* | 12 |  | rs219870 | *PAK7* | 20 |
| rs10213444 | *MAPK10* | 4 |  | rs2300266 | *GRIN2B* | 12 |  | rs6056766 | *PAK7* | 20 |
| rs6531915 | *MAPK10* | 4 |  | rs2300267 | *GRIN2B* | 12 |  | rs6086964 | *PAK7* | 20 |
| rs2290882 | *MAPK10* | 4 |  | rs11055625 | *GRIN2B* | 12 |  | rs16996186 | *PAK7* | 20 |
| rs4693142 | *MAPK10* | 4 |  | rs12303001 | *GRIN2B* | 12 |  | rs12624866 | *PAK7* | 20 |
| rs7677400 | *MAPK10* | 4 |  | rs2300272 | *GRIN2B* | 12 |  | rs6056773 | *PAK7* | 20 |
| rs13112340 | *MAPK10* | 4 |  | rs220573 | *GRIN2B* | 12 |  | rs6516479 | *PAK7* | 20 |
| rs4608765 | *MAPK10* | 4 |  | rs220575 | *GRIN2B* | 12 |  | rs10485734 | *PAK7* | 20 |
| rs6531925 | *MAPK10* | 4 |  | rs2160730 | *GRIN2B* | 12 |  | rs6086972 | *PAK7* | 20 |
| rs2869433 | *MAPK10* | 4 |  | rs220583 | *GRIN2B* | 12 |  | rs6039533 | *PAK7* | 20 |
| rs10008238 | *MAPK10* | 4 |  | rs2111512 | *GRIN2B* | 12 |  | rs4816158 | *PAK7* | 20 |
| rs2904096 | *MAPK10* | 4 |  | rs2284422 | *GRIN2B* | 12 |  | rs6039540 | *PAK7* | 20 |
| rs12508801 | *MAPK10* | 4 |  | rs220590 | *GRIN2B* | 12 |  | rs6118681 | *PAK7* | 20 |
| rs4235080 | *MAPK10* | 4 |  | rs220593 | *GRIN2B* | 12 |  | rs17408919 | *PAK7* | 20 |
| rs6823664 | *MAPK10* | 4 |  | rs220597 | *GRIN2B* | 12 |  | rs2423395 | *PAK7* | 20 |
| rs12507758 | *MAPK10* | 4 |  | rs220599 | *GRIN2B* | 12 |  | rs6133735 | *PAK7* | 20 |
| rs4488910 | *MAPK10* | 4 |  | rs2160732 | *GRIN2B* | 12 |  | rs6039542 | *PAK7* | 20 |
| rs7658485 | *MAPK10* | 4 |  | rs2160734 | *GRIN2B* | 12 |  | rs2423401 | *PAK7* | 20 |
| rs2240795 | *CAMK2A* | 5 |  | rs11612353 | *GRIN2B* | 12 |  | rs6118687 | *PAK7* | 20 |
| rs4705431 | *CAMK2A* | 5 |  | rs2284424 | *GRIN2B* | 12 |  | rs2254000 | *PAK7* | 20 |
| rs887346 | *CAMK2A* | 5 |  | rs2284425 | *GRIN2B* | 12 |  | rs2254003 | *PAK7* | 20 |
| rs980272 | *CAMK2A* | 5 |  | rs2300273 | *GRIN2B* | 12 |  | rs2423412 | *PAK7* | 20 |
| rs2241694 | *CAMK2A* | 5 |  | rs12305339 | *GRIN2B* | 12 |  | rs2423413 | *PAK7* | 20 |
| rs2241695 | *CAMK2A* | 5 |  | rs1861786 | *GRIN2B* | 12 |  | rs6039545 | *PAK7* | 20 |
| rs6885505 | *CAMK2A* | 5 |  | rs1861787 | *GRIN2B* | 12 |  | rs6077585 | *PAK7* | 20 |
| rs2053053 | *CAMK2A* | 5 |  | rs7297313 | *GRIN2B* | 12 |  | rs6141008 | *PAK7* | 20 |
| rs919741 | *CAMK2A* | 5 |  | rs2284428 | *GRIN2B* | 12 |  | rs2050104 | *PAK7* | 20 |
| rs3822607 | *CAMK2A* | 5 |  | rs10845844 | *GRIN2B* | 12 |  | rs8184671 | *PAK7* | 20 |
| rs17111079 | *CAMK2A* | 5 |  | rs11055651 | *GRIN2B* | 12 |  | rs2050105 | *PAK7* | 20 |
| rs6869180 | *CAMK2A* | 5 |  | rs11835020 | *GRIN2B* | 12 |  | rs8120907 | *PAK7* | 20 |
| rs3797617 | *CAMK2A* | 5 |  | rs10845847 | *GRIN2B* | 12 |  | rs2223565 | *PAK7* | 20 |
| rs3776825 | *CAMK2A* | 5 |  | rs11055657 | *GRIN2B* | 12 |  | rs6056812 | *PAK7* | 20 |
| rs4958456 | *CAMK2A* | 5 |  | rs10845852 | *GRIN2B* | 12 |  | rs6108331 | *PAK7* | 20 |
| rs3756578 | *CAMK2A* | 5 |  | rs10845853 | *GRIN2B* | 12 |  | rs6086989 | *PAK7* | 20 |
| rs7711562 | *CAMK2A* | 5 |  | rs7134097 | *GRIN2B* | 12 |  | rs2423431 | *PAK7* | 20 |
| rs3756577 | *CAMK2A* | 5 |  | rs10845856 | *GRIN2B* | 12 |  | rs6086998 | *PAK7* | 20 |
| rs7701427 | *CAMK2A* | 5 |  | rs12579598 | *GRIN2B* | 12 |  | rs2423438 | *PAK7* | 20 |
| rs10463293 | *CAMK2A* | 5 |  | rs10492141 | *GRIN2B* | 12 |  | rs6141018 | *PAK7* | 20 |
| rs4958445 | *CAMK2A* | 5 |  | rs10492142 | *GRIN2B* | 12 |  | rs6039561 | *PAK7* | 20 |
| rs4958902 | *CAMK2A* | 5 |  | rs7314376 | *GRIN2B* | 12 |  | rs2423441 | *PAK7* | 20 |
| rs874083 | *CAMK2A* | 5 |  | rs11055671 | *GRIN2B* | 12 |  | rs2423443 | *PAK7* | 20 |
| rs1897559 | *CAMK2A* | 5 |  | rs10160840 | *GRIN2B* | 12 |  | rs7274475 | *PAK7* | 20 |
| rs10515639 | *CAMK2A* | 5 |  | rs4764039 | *GRIN2B* | 12 |  | rs2423456 | *PAK7* | 20 |
| rs1432833 | *CAMK2A* | 5 |  | rs7297101 | *GRIN2B* | 12 |  | rs7274775 | *PAK7* | 20 |
| rs919740 | *CAMK2A* | 5 |  | rs2216344 | *GRIN2B* | 12 |  | rs4813896 | *PAK7* | 20 |
| rs873592 | *CAMK2A* | 5 |  | rs2098469 | *GRIN2B* | 12 |  | rs1033470 | *PAK7* | 20 |
| rs4286653 | *CAMK2A* | 5 |  | rs10459061 | *GRIN2B* | 12 |  | rs4813897 | *PAK7* | 20 |
| rs891944 | *CAMK2A* | 5 |  | rs219872 | *GRIN2B* | 12 |  | rs6141026 | *PAK7* | 20 |
| rs891943 | *CAMK2A* | 5 |  | rs918168 | *GRIN2B* | 12 |  | rs13045381 | *PAK7* | 20 |
| rs17118812 | *HBEGF* | 5 |  | rs219876 | *GRIN2B* | 12 |  | rs6108334 | *PAK7* | 20 |
| rs7268 | *HBEGF* | 5 |  | rs219904 | *GRIN2B* | 12 |  | rs6133746 | *PAK7* | 20 |
| rs13385 | *HBEGF* | 5 |  | rs172677 | *GRIN2B* | 12 |  | rs2423462 | *PAK7* | 20 |
| rs2237077 | *HBEGF* | 5 |  | rs219922 | *GRIN2B* | 12 |  | rs13045260 | *PAK7* | 20 |
| rs4150196 | *HBEGF* | 5 |  | rs1592669 | *GRIN2B* | 12 |  | rs1569625 | *PAK7* | 20 |
| rs1862176 | *HBEGF* | 5 |  | rs766831 | *GRIN2B* | 12 |  | rs2423467 | *PAK7* | 20 |
| rs6879217 | *HBEGF* | 5 |  | rs219931 | *GRIN2B* | 12 |  | rs10470072 | *PAK7* | 20 |
| rs717097 | *HBEGF* | 5 |  | rs4432098 | *GRIN2B* | 12 |  | rs926502 | *PAK7* | 20 |
| rs6860077 | *HBEGF* | 5 |  | rs12829455 | *GRIN2B* | 12 |  | rs4566434 | *PAK7* | 20 |
| rs3111519 | *MAPK9* | 5 |  | rs4764043 | *GRIN2B* | 12 |  | rs6118724 | *PAK7* | 20 |
| rs13435 | *MAPK9* | 5 |  | rs12820037 | *GRIN2B* | 12 |  | rs2327225 | *PAK7* | 20 |
| rs6894217 | *MAPK9* | 5 |  | rs11055697 | *GRIN2B* | 12 |  | rs6141031 | *PAK7* | 20 |
| rs4601008 | *MAPK9* | 5 |  | rs7298664 | *GRIN2B* | 12 |  | rs6039571 | *PAK7* | 20 |
| rs4362908 | *MAPK9* | 5 |  | rs10505778 | *GRIN2B* | 12 |  | rs6056855 | *PAK7* | 20 |
| rs13190345 | *MAPK9* | 5 |  | rs1421106 | *GRIN2B* | 12 |  | rs6108337 | *PAK7* | 20 |
| rs11249687 | *MAPK9* | 5 |  | rs1421108 | *GRIN2B* | 12 |  | rs6516498 | *PAK7* | 20 |
| rs17080136 | *MAPK9* | 5 |  | rs3764030 | *GRIN2B* | 12 |  | rs6039576 | *PAK7* | 20 |
| rs6868333 | *MAPK9* | 5 |  | rs11047887 | *KRAS* | 12 |  | rs2223569 | *PAK7* | 20 |
| rs7730091 | *MAPK9* | 5 |  | rs9266 | *KRAS* | 12 |  | rs6056871 | *PAK7* | 20 |
| rs2112593 | *MAPK9* | 5 |  | rs12579073 | *KRAS* | 12 |  | rs6056873 | *PAK7* | 20 |
| rs730688 | *MAPK9* | 5 |  | rs10842513 | *KRAS* | 12 |  | rs994487 | *PAK7* | 20 |
| rs7702792 | *MAPK9* | 5 |  | rs11047912 | *KRAS* | 12 |  | rs6056875 | *PAK7* | 20 |
| rs3812066 | *MAPK9* | 5 |  | rs17388587 | *KRAS* | 12 |  | rs6141035 | *PAK7* | 20 |
| rs3812067 | *MAPK9* | 5 |  | rs6487464 | *KRAS* | 12 |  | rs10485735 | *PAK7* | 20 |
| rs745749 | *MAPK9* | 5 |  | rs10842514 | *KRAS* | 12 |  | rs2206481 | *PAK7* | 20 |
| rs3749791 | *MAPK9* | 5 |  | rs10842515 | *KRAS* | 12 |  | rs6141038 | *PAK7* | 20 |
| rs17286536 | *NRG2* | 5 |  | rs12813551 | *KRAS* | 12 |  | rs6056890 | *PAK7* | 20 |
| rs17118468 | *NRG2* | 5 |  | rs2970532 | *KRAS* | 12 |  | rs6056891 | *PAK7* | 20 |
| rs2302984 | *NRG2* | 5 |  | rs17389103 | *KRAS* | 12 |  | rs2050108 | *PAK7* | 20 |
| rs4912894 | *NRG2* | 5 |  | rs11047919 | *KRAS* | 12 |  | rs6087032 | *PAK7* | 20 |
| rs3756675 | *NRG2* | 5 |  | rs4368021 | *KRAS* | 12 |  | rs6118746 | *PAK7* | 20 |
| rs1800954 | *NRG2* | 5 |  | rs12320328 | *KRAS* | 12 |  | rs6516503 | *PAK7* | 20 |
| rs6580288 | *NRG2* | 5 |  | rs6487465 | *KRAS* | 12 |  | rs6056909 | *PAK7* | 20 |
| rs3777102 | *NRG2* | 5 |  | rs1144943 | *MDM2* | 12 |  | rs221015 | *PAK7* | 20 |
| rs1422186 | *NRG2* | 5 |  | rs1470383 | *MDM2* | 12 |  | rs221013 | *PAK7* | 20 |
| rs10515509 | *NRG2* | 5 |  | rs769412 | *MDM2* | 12 |  | rs6056922 | *PAK7* | 20 |
| rs4912936 | *NRG2* | 5 |  | rs2301723 | *PTPN11* | 12 |  | rs761226 | *PAK7* | 20 |
| rs6895139 | *NRG2* | 5 |  | rs17822304 | *PTPN11* | 12 |  | rs12479522 | *PAK7* | 20 |
| rs2060296 | *NRG2* | 5 |  | rs12423190 | *PTPN11* | 12 |  | rs6129760 | *PLCG1* | 20 |
| rs264336 | *NRG2* | 5 |  | rs11066322 | *PTPN11* | 12 |  | rs12624863 | *PLCG1* | 20 |
| rs9686629 | *NRG2* | 5 |  | rs2239167 | *PTPN6* | 12 |  | rs753381 | *PLCG1* | 20 |
| rs7736276 | *NRG2* | 5 |  | rs1044189 | *PTPN6* | 12 |  | rs2235360 | *PLCG1* | 20 |
| rs197197 | *NRG2* | 5 |  | rs744167 | *PTPN6* | 12 |  | rs2235366 | *PLCG1* | 20 |
| rs11746363 | *NRG2* | 5 |  | rs2695783 | *RNF41* | 12 |  | rs718630 | *PTPN1* | 20 |
| rs264347 | *NRG2* | 5 |  | rs7962107 | *RNF41* | 12 |  | rs6020563 | *PTPN1* | 20 |
| rs155348 | *NRG2* | 5 |  | rs2498804 | *AKT1* | 14 |  | rs6020566 | *PTPN1* | 20 |
| rs266022 | *NRG2* | 5 |  | rs2494738 | *AKT1* | 14 |  | rs932420 | *PTPN1* | 20 |
| rs6891114 | *NRG2* | 5 |  | rs4983387 | *AKT1* | 14 |  | rs6067474 | *PTPN1* | 20 |
| rs265150 | *NRG2* | 5 |  | rs4983559 | *AKT1* | 14 |  | rs2426157 | *PTPN1* | 20 |
| rs7703099 | *NRG2* | 5 |  | rs17183482 | *FOS* | 14 |  | rs3787335 | *PTPN1* | 20 |
| rs265158 | *NRG2* | 5 |  | rs8004059 | *FOS* | 14 |  | rs2143511 | *PTPN1* | 20 |
| rs645850 | *NRG2* | 5 |  | rs7101 | *FOS* | 14 |  | rs6063533 | *PTPN1* | 20 |
| rs2115048 | *NRG2* | 5 |  | rs1063169 | *FOS* | 14 |  | rs941798 | *PTPN1* | 20 |
| rs265148 | *NRG2* | 5 |  | rs7154366 | *FOS* | 14 |  | rs6512654 | *PTPN1* | 20 |
| rs6580353 | *NRG2* | 5 |  | rs1009392 | *FOS* | 14 |  | rs3787341 | *PTPN1* | 20 |
| rs2916092 | *NRG2* | 5 |  | rs3736807 | *HSP90AA1* | 14 |  | rs11698821 | *PTPN1* | 20 |
| rs155939 | *NRG2* | 5 |  | rs11621560 | *HSP90AA1* | 14 |  | rs6020608 | *PTPN1* | 20 |
| rs6889324 | *NRG2* | 5 |  | rs7160651 | *HSP90AA1* | 14 |  | rs754118 | *PTPN1* | 20 |
| rs40419 | *PIK3R1* | 5 |  | rs10873531 | *HSP90AA1* | 14 |  | rs718050 | *PTPN1* | 20 |
| rs706713 | *PIK3R1* | 5 |  | rs7157967 | *HSP90AA1* | 14 |  | rs1060402 | *PTPN1* | 20 |
| rs13173003 | *PIK3R1* | 5 |  | rs1190596 | *HSP90AA1* | 14 |  | rs6094373 | *SRC* | 20 |
| rs7713645 | *PIK3R1* | 5 |  | rs1190592 | *HSP90AA1* | 14 |  | rs747182 | *SRC* | 20 |
| rs7709243 | *PIK3R1* | 5 |  | rs8014112 | *NFATC4* | 14 |  | rs6018027 | *SRC* | 20 |
| rs12652661 | *PIK3R1* | 5 |  | rs7148384 | *NFATC4* | 14 |  | rs6094509 | *SRC* | 20 |
| rs251406 | *PIK3R1* | 5 |  | rs1950500 | *NFATC4* | 14 |  | rs12329503 | *SRC* | 20 |
| rs173702 | *PIK3R1* | 5 |  | rs2228233 | *NFATC4* | 14 |  | rs6018199 | *SRC* | 20 |
| rs251404 | *PIK3R1* | 5 |  | rs11628929 | *NFATC4* | 14 |  | rs6018257 | *SRC* | 20 |
| rs4122269 | *PIK3R1* | 5 |  | rs8008583 | *NFATC4* | 14 |  | rs11905013 | *SRC* | 20 |
| rs40318 | *PIK3R1* | 5 |  | rs6573766 | *NFATC4* | 14 |  | rs4810624 | *SRC* | 20 |
| rs1823023 | *PIK3R1* | 5 |  | rs2227276 | *SOS2* | 14 |  | rs178084 | *CRKL* | 22 |
| rs173703 | *PIK3R1* | 5 |  | rs10151119 | *SOS2* | 14 |  | rs3819652 | *CRKL* | 22 |
| rs706716 | *PIK3R1* | 5 |  | rs2024809 | *SOS2* | 14 |  | rs13057428 | *CRKL* | 22 |
| rs6893676 | *PIK3R1* | 5 |  | rs2229869 | *SOS2* | 14 |  | rs5752284 | *CRKL* | 22 |
| rs34303 | *PIK3R1* | 5 |  | rs3742467 | *SOS2* | 14 |  | rs2266953 | *CRKL* | 22 |
| rs863818 | *PIK3R1* | 5 |  | rs8017367 | *SOS2* | 14 |  | rs2018682 | *CRKL* | 22 |
| rs16897561 | *PIK3R1* | 5 |  | rs7140473 | *SOS2* | 14 |  | rs9607241 | *MAPK1* | 22 |
| rs34309 | *PIK3R1* | 5 |  | rs4898652 | *SOS2* | 14 |  | rs2298432 | *MAPK1* | 22 |
| rs10515074 | *PIK3R1* | 5 |  | rs2144575 | *SOS2* | 14 |  | rs743409 | *MAPK1* | 22 |
| rs2302975 | *PIK3R1* | 5 |  | rs743076 | *SOS2* | 14 |  | rs2283792 | *MAPK1* | 22 |
| rs3730082 | *PIK3R1* | 5 |  | rs4776783 | *MAP2K1* | 15 |  | rs7290469 | *MAPK1* | 22 |
| rs6876003 | *PIK3R1* | 5 |  | rs1549854 | *MAP2K1* | 15 |  | rs1892846 | *MAPK1* | 22 |
| rs3815701 | *PIK3R1* | 5 |  | rs1432442 | *MAP2K1* | 15 |  | rs8141815 | *MAPK1* | 22 |
| rs34306 | *PIK3R1* | 5 |  | rs1432441 | *MAP2K1* | 15 |  | rs9610417 | *MAPK1* | 22 |
| rs1550805 | *PIK3R1* | 5 |  | rs12050732 | *MAP2K1* | 15 |  | rs8136867 | *MAPK1* | 22 |
| rs895304 | *PIK3R1* | 5 |  | rs4255740 | *MAP2K1* | 15 |  | rs16987839 | *NF2* | 22 |
| rs10941232 | *PRLR* | 5 |  | rs933996 | *NEDD4* | 15 |  | rs5763373 | *NF2* | 22 |
| rs249522 | *PRLR* | 5 |  | rs11071224 | *NEDD4* | 15 |  | rs2530664 | *NF2* | 22 |
| rs401694 | *PRLR* | 5 |  | rs8034917 | *NEDD4* | 15 |  | rs6006220 | *NF2* | 22 |
| rs37389 | *PRLR* | 5 |  | rs4520787 | *NEDD4* | 15 |  | rs2857639 | *NF2* | 22 |
| rs249537 | *PRLR* | 5 |  | rs2288344 | *NEDD4* | 15 |  | rs2857642 | *NF2* | 22 |
| rs7734558 | *PRLR* | 5 |  | rs2288345 | *NEDD4* | 15 |  | rs2527335 | *NF2* | 22 |
| rs7705243 | *PRLR* | 5 |  | rs2438129 | *NEDD4* | 15 |  | rs2009354 | *NF2* | 22 |
| rs2914108 | *PRLR* | 5 |  | rs2288346 | *NEDD4* | 15 |  | rs737787 | *NF2* | 22 |
| rs2962089 | *PRLR* | 5 |  | rs2438128 | *NEDD4* | 15 |  | rs2857651 | *NF2* | 22 |
| rs12109986 | *PRLR* | 5 |  | rs1912407 | *NEDD4* | 15 |  | rs2530678 | *NF2* | 22 |
| rs6897987 | *PRLR* | 5 |  | rs17238489 | *NEDD4* | 15 |  | rs756053 | *NF2* | 22 |
| rs6880595 | *PRLR* | 5 |  | rs9972348 | *NEDD4* | 15 |  | rs2267151 | *NF2* | 22 |
| rs6451185 | *PRLR* | 5 |  | rs8027843 | *NEDD4* | 15 |  | rs1009147 | *NF2* | 22 |
| rs4703503 | *PRLR* | 5 |  | rs17819282 | *NEDD4* | 15 |  | rs1009148 | *NF2* | 22 |
| rs16872491 | *PRLR* | 5 |  | rs10518827 | *NEDD4* | 15 |  | rs2530682 | *NF2* | 22 |
| rs1039427 | *PRLR* | 5 |  | rs10518828 | *NEDD4* | 15 |  | rs5906428 | *ARAF* | X |
| rs7729932 | *PRLR* | 5 |  | rs12593446 | *NEDD4* | 15 |  | rs2283736 | *ARAF* | X |
| rs11952661 | *PRLR* | 5 |  | rs7174459 | *NEDD4* | 15 |  | rs2071777 | *ARAF* | X |
| rs6895193 | *PRLR* | 5 |  | rs17819300 | *NEDD4* | 15 |  | rs723556 | *ARAF* | X |
| rs7700286 | *PRLR* | 5 |  | rs2271289 | *NEDD4* | 15 |  | rs2765814 | *ELK1* | X |
| rs13436213 | *PRLR* | 5 |  | rs2175104 | *NEDD4* | 15 |  |  |  |  |

**^a^** SNPs were ranked by chromosomes and location within each chromosome.
