# Supplementary material for: Genetic Variants in Epidermal Growth Factor Receptor Pathway Genes and Risk of Esophageal Squamous Cell Carcinoma and Gastric Cancer in a Chinese Population
Source: PLoS One. 2013 Jul 18;8(7):e68999. doi: 10.1371/journal.pone.0068999 (PMC3715462; doi:10.1371/journal.pone.0068999)
Supplement: Table S3 — Characteristics of the study participants. (DOCX) [file pone.0068999.s003.docx]

**Table S3. Characteristics of the study participants ^a^**

|  | **Controls** | **ESCC**  **cases** | **GC cases** | | |
| --- | --- | --- | --- | --- | --- |
|  |  |  | **Total** | **Cardia** | **Noncardia** |
| **Total** | 2111 | 1942 | 1758 | 1126 | 632 |
| Age, mean (SD), year | 56.0 (9.5) | 56.0 (8.6) | 56.3 (9.5) | 57.2 (9.0) | 54.6 (10.1) |
| Female (yes, %) | 677 (32.1) | 778 (40.1) | 416 (23.7) | 242 (21.5) | 174 (27.5) |
| Smoking (ever, %) | 1223 (57.9) | 1008 (51.9) | 1136 (64.6) | 738 (65.5) | 398 (63.0) |
| Alcohol intake (ever, %) | 303 (14.4) | 315 (16.2) | 339 (19.3) | 214 (19.0) | 125 (19.8) |
| Family history of UGI cancer (yes, %) | 478 (22.6) | 541 (27.9) | 427 (24.3) | 297 (26.4) | 130 (20.6) |
| **Shanxi UGI Cancer Genetics Project** | 1660 | 1421 | 1395 | 864 | 531 |
| Age, mean (SD), year | 57.8 (9.2) | 58.1 (8.1) | 57.8 (9.4) | 59.3 (8.4) | 55.4 (10.4) |
| Female (yes, %) | 434 (26.1) | 923 (35.1) | 264 (18.9) | 133 (15.4) | 131 (24.7) |
| Smoking (ever, %) | 1079 (65.0) | 861 (60.6) | 990 (71.0) | 628 (72.7) | 362 (68.2) |
| Alcohol intake (ever, %) | 290 (17.5) | 309 (21.8) | 329 (23.6) | 206 (23.8) | 123 (23.2) |
| Family history of UGI cancer (yes, %) | 338 (20.4) | 343 (24.1) | 298 (21.4) | 204 (23.6) | 94 (17.7) |
| **Linxian Nutrition Intervention Trials** | 451 | 521 | 363 | 262 | 101 |
| Age, mean (SD), year | 49.5 (7.4) | 50.0 (6.9) | 50.4 (6.9) | 50.3 (7.1) | 50.6 (6.6) |
| Female (yes, %) | 243 (53.9) | 280 (53.7) | 152 (41.9) | 109 (41.6) | 43 (42.6) |
| Smoking (ever, %) | 144 (31.9) | 147 (28.2) | 146 (40.2) | 110 (42.0) | 36 (35.6) |
| Alcohol intake (ever, %) | 13 (2.9) | 6 (1.15) | 10 (2.8) | 8 (3.05) | 2 (1.98) |
| Family history of UGI cancer (yes, %) | 140 (31.0) | 198 (38.0) | 129 (35.5) | 93 (35.5) | 36 (35.6) |

**^a^** Participants were enrolled from the Shanxi Upper Gastrointestinal Cancer Genetics Project (Shanxi) and Linxian Nutrition Intervention Trials (NIT).
